# Contribution of NtZIP1-Like to the Regulation of Zn Homeostasis

**DOI:** 10.3389/fpls.2018.00185

**Published:** 2018-02-16

**Authors:** Anna Papierniak, Katarzyna Kozak, Maria Kendziorek, Anna Barabasz, Małgorzata Palusińska, Jerzy Tiuryn, Bohdan Paterczyk, Lorraine E. Williams, Danuta M. Antosiewicz

**Affiliations:** ^1^Institute of Experimental Plant Biology and Biotechnology, Faculty of Biology, University of Warsaw, Warsaw, Poland; ^2^Faculty of Mathematics, Informatics, and Mechanics, University of Warsaw, Warsaw, Poland; ^3^Laboratory of Electron and Confocal Microscopy, Faculty of Biology, University of Warsaw, Warsaw, Poland; ^4^Biological Sciences, University of Southampton, Southampton, United Kingdom

**Keywords:** zinc, tobacco, ZIP, NtZIP1-like, yeast complementation

## Abstract

Tobacco has frequently been suggested as a candidate plant species for use in phytoremediation of metal contaminated soil but knowledge on the regulation of its metal-homeostasis is still in the infancy. To identify new tobacco metal transport genes that are involved in Zn homeostasis a bioinformatics study using the tobacco genome information together with expression analysis was performed. Ten new tobacco metal transport genes from the ZIP, NRAMP, MTP, and MRP/ABCC families were identified with expression levels in leaves that were modified by exposure to Zn excess. Following exposure to high Zn there was upregulation of *NtZIP11-like*, *NtNRAMP3*, three isoforms of *NtMTP2*, three MRP/ABCC genes (*NtMRP5-like*, *NtMRP10-like*, and *NtMRP14 like*) and downregulation of *NtZIP1-like* and *NtZIP4.* This suggests their involvement in several processes governing the response to Zn-related stress and in the efficiency of Zn accumulation (uptake, sequestration, and redistribution). Further detailed analysis of NtZIP1-like provided evidence that it is localized at the plasma membrane and is involved in Zn but not Fe and Cd transport. *NtZIP1-like* is expressed in the roots and shoots, and is regulated developmentally and in a tissue-specific manner. It is highly upregulated by Zn deficiency in the leaves and the root basal region but not in the root apical zone (region of maturation and absorption containing root hairs). Thus NtZIP1-like is unlikely to be responsible for Zn uptake by the root apical region but rather in the uptake by root cells within the already mature basal zone. It is downregulated by Zn excess suggesting it is involved in a mechanism to protect the root and leaf cells from accumulating excess Zn.

## Introduction

Tobacco (*Nicotiana tabacum* L cv. Xanthi) has frequently been considered for phytoremediation purposes because of its high biomass and ability to take up and accumulate in leaves high amounts of metals, including zinc (Zn) (Vangronsveld et al., 2009; Herzig et al., 2014; Vera-Estrella et al., 2017). To improve its capacity to take up and store metals in shoots, it has been transformed with a number of metal homeostasis genes, but with limited success (Gisbert et al., 2003; Martínez et al., 2006; Gorinova et al., 2007; Wojas et al., 2008, 2009; Korenkov et al., 2009; Siemianowski et al., 2011; Barabasz et al., 2013; Wang et al., 2015). Recently, it was shown that when expressing metal transporters to engineer new metal-related traits, a major part of the resulting phenotype was due to the modulation of endogenous gene expression (Barabasz et al., 2016; Kendziorek et al., 2016). Therefore, a greater understanding of Zn-homeostasis mechanisms is required to successfully genetically modify the efficiency of Zn accumulation in shoots. Maintaining high Zn in the above ground organs depends on three major processes operating efficiently: Zn uptake from the soil, root-to-shoot translocation and storage in leaves without detrimental toxic effects.

Zn uptake is thought to be mediated primarily by ZIP (ZRT∖IRT related Protein) metal transporters. In *Arabidopsis thaliana* AtZIP2, AtIRT1 and AtIRT3 residing in the plasma membrane have been identified as key players in Zn acquisition by roots (Vert et al., 2002; Lin et al., 2009; Palmer and Guerinot, 2009; Milner et al., 2013). The root-to-shoot translocation of Zn (and other metals) depends on two main factors: the ability to store the metal in the roots; and the efficiency of its loading into xylem vessels. It has been shown that HMAs (Heavy-Metal ATPases) which belong to the P_1B_-ATPase family (Williams et al., 2000; Williams and Mills, 2005) are involved in both processes. HMA3, identified in *A. thaliana* and rice, localized in the tonoplast of root cortical cells, limits translocation of Cd from the roots to the shoots by sequestrating the metal into the root vacuoles. There is a suggestion that it could also transport Zn into the vacuoles and control the amount of Zn available for xylem loading and thus the efficiency of its translocation to the shoot (Morel et al., 2009; Ueno et al., 2010; Miyadate et al., 2011). The efficiency of the next step in Zn translocation to shoots - loading of a metal into the xylem vessels, is under the control of two genes with overlapping function, *HMA2* and *HMA4* (Hussain et al., 2004; Verret et al., 2004; Wong and Cobbett, 2009). Encoded proteins are localized in the roots at the plasma membrane of xylem parenchyma cells where they are responsible for Zn (and also Cd) efflux to the xylem. Decreased translocation of Zn to shoots in the *athma2athma4* double mutant led to severe Zn deficiency (Hussain et al., 2004; Wong and Cobbett, 2009; Mills et al., 2012).

Zn transported to the shoots is stored primarily in the mesophyll cells of leaves. Its level of accumulation depends on the ability of the mesophyll cells to store the metal without toxicity. This complex process involves efficient metal import and its loading into the vacuoles, but also regulated redistribution from this compartment. Currently we are far from having a clear picture of all the elements involved. The potential players include members of several transport families. The *ZIP* genes play a diverse roles, and those present in the plasma membrane are responsible for Zn uptake, while others localized in the tonoplast could contribute to control of Zn release from vacuoles (Guerinot, 2000; Milner et al., 2013; Ricachenevsky et al., 2015). Accumulation of metal/s in the vacuoles also depends on the NRAMP (Natural Resistance-Associated Macrophage Protein) family. Members of this family transport Fe and Mn, while Cd, Zn or Ni can also serve as substrates for some (Nevo and Nelson, 2006; Ricachenevsky et al., 2015). AtNRAMP1 is a plasma membrane Mn uptake system in roots of *A*. *thaliana*; Cailliatte et al. (2010), while NRAMP3 and NRAMP4 are involved in metal release (Mn and Fe) from vacuoles in leaves and seeds (Lanquar et al., 2005, 2010). High expression of *NRAMP3* and *NRAMP4* genes was noted in the leaves of Zn/Cd hyperaccumulating *A. halleri* (Weber et al., 2004) and *Thlaspi caerulescens*. Both *TcNRAMP3* and *TcNRAMP4* were implicated in metal hypertolerance, but the precise role is yet to be determined (Oomen et al., 2009). Loading of metals into vacuoles is provided by the members of the MTP (Metal Tolerance Proteins) family. Residing in the tonoplast, they are involved in sequestration primarily Zn in the vacuoles, but other metals such as Fe, Mn, Cd, Ni or Co can also be substrates for some family members (Gustin et al., 2011; Menguer et al., 2013; Ricachenevsky et al., 2013; Farthing et al., 2017). However, some MTPs are localized in the plasma membrane, and they remove cations from the cytoplasm to the cell wall (Menguer et al., 2013; Migocka et al., 2015). In the leaves, a key protein for Zn sequestration and detoxification is the vacuolar protein MTP1. AtMTP1 from *A thaliana* contributes to Zn accumulation in leaves and to basal Zn tolerance by sequestering Zn in vacuoles (Kobae et al., 2004; Desbrosses-Fonrouge et al., 2005; Ricachenevsky et al., 2013). MTP1 also has a function in Zn accumulation in shoots of Zn hyperaccumulators such as *A. halleri* (Dräger et al., 2004) or *Thlaspi goesingense* (Gustin et al., 2009).

Despite a broad interest in the use of tobacco to remove metals from contaminated soil, knowledge of the metal homeostatic processes in this species is still in its infancy. Only a few metal transport genes have been cloned and characterized so far. *NtPDR3* (pleiotropic drug resistance) from *Nicotiana tabacum* was shown to be highly expressed under Fe-deficiency conditions suggesting its involvement in iron homeostasis (Ducos et al., 2005). MTP family members involved in Zn and Co metabolism were cloned from *Nicotiana tabacum* (*NtMTP1a, NtMTP1b*) and *Nicotiana glauca* (*NgMTP1*) (Shingu et al., 2005). Also, two orthologs of the *Arabidopsis thaliana HMA2* and *HMA4* were identified in tobacco, *NtHMAα* and *NtHMAβ.* Similar to *Arabidopsis* genes, *NtHMAα* and *NtHMAβ* are responsible for Zn and Cd root-to-shoot translocation (Hermand et al., 2014; Liedschulte et al., 2017). Furthermore, studies performed on tobacco BY-2 cells identified two genes encoding Fe uptake proteins; NtNRAMP3 and NtZIP1 (Sano et al., 2012). A second ZIP family member from tobacco, NtIRT1, was also shown to transport Fe, and its expression depended on the level of Fe and Cd in the medium (Yoshihara et al., 2006; Hodoshima et al., 2007).

To learn more about the molecular mechanism regulating Zn accumulation in tobacco leaves, the aim of this study was to identify the members of the following key metal transport families that could be involved in regulating Zn levels in the leaf blades: ZIP, NRAMP, and MTP. Moreover, taking into account very limited knowledge on the possible contribution of MRPs (multidrug resistance-associated proteins) family members to detoxification of metals, they were also included. MRP/ABCC (Klein et al., 2006; Verrier et al., 2008) transporters are a ubiquitous subfamily of ABC (ATP Binding Casette) transporters which catalyze the export of substrates out of the cytosol in an ATP-dependent manner. Their involvement in Zn and Cd hypertolerance in *N. caerulescens* was shown by Halimaa et al. (2014) and also in the detoxification of Cd (Bovet et al., 2003, 2005; Wojas et al., 2007; Gaillard et al., 2008).

The major focus in this study was on proteins mediating Zn import into the tobacco leaf cells from the ZIP family. They were identified and initially characterized in several organisms, for example in *Arabidopsis* (15 ZIPs; Grotz and Guerinot, 2006), rice (16 ZIPs; Chen et al., 2008), bean (23 ZIPs; Astudillo et al., 2013) and more recently, wheat (Evens et al., 2017). In addition to Zn, ZIPs mediate transport of Mn, Fe, Ni, or Cu. Detailed analysis of the role of ZIP genes is still lacking for many of those identified. Their function has been anticipated primarily based on metal-specific (Zn, Fe, Mn, Cd, and Cu) and concentration-dependent (deficit/sufficient/excess) regulation of ZIP expression in organs. (Bashir et al., 2012; Sinclair and Krämer, 2012; Milner et al., 2013; Evens et al., 2017; Nazri et al., 2017).

Here, bioinformatics analysis of tobacco genome data was performed to identify sequences homologous to chosen *Arabidopsis thaliana* metal transport genes, and subsequent expression analysis led to the identification of the *NtZIP1-like*. It was cloned and characterized indicating its specific function in the regulation of Zn homeostasis in tobacco leaves.

## Materials and Methods

### Plant Material and Growth Conditions

All experiments were performed on tobacco plants (*Nicotiana tabacum* var. Xanthi). Surface sterilized seeds (8% sodium hypochloride w/v for 2 min) were germinated on Petri dishes positioned vertically containing quarter-strength Knop’s medium, 2% sucrose (w/v) and 1% agar (w/v) (Barabasz et al., 2013). Three weeks following germination, seedlings were transferred to hydroponic conditions. They were cultivated in 2-L pots (5 plants per pot) on aerated quarter-strength Knop’s medium for 2 weeks to allow them to adjust to hydroponic conditions. The nutrient solution was renewed every 3–4 days (unless indicated otherwise). Five-week-old plants (3 weeks on plates and 2 weeks on hydroponics) were further used for experiments. They were exposed to chosen Zn (as ZnSO_4_) concentrations added to quarter-strength Knop’s medium. Details are given in the subsections 2.3 and 2.9 below. At the end of each experiment, the plant samples were collected always at the same time of the day (between 10–12 AM). The quarter-strength Knop’s medium (containing 0.5 μM Zn) was used as a reference (control) medium in parallel to applied Zn treatments.

Plants were cultivated in a growth chamber at temperature 23/16°C day/night, 40–50% humidity, 16 h photoperiod, and quantum flux density [photosynthetically active radiation (PAR)] 250 mmol m^-2^ s^-1^, fluorescent Flora tubes.

### Database Search for Putative Tobacco Metal Transport Family Members

The goal was to identify potential tobacco metal transporters involved in the accumulation of Zn in leaves. There are two sources of tobacco sequences to be used for gene mining. First, the complete genomic tobacco sequence has recently been made available to the public in GenBank with accession code AWOK00000000 (Sierro et al., 2013, 2014). Second, there is the NCBI database which provides already annotated genes from a range of species including tobacco. In tobacco, only several metal transporters have been already identified, cloned and characterized, some more were annotated and their sequences could be found in the NCBI database. Thus, NCBI database likely does not contain all tobacco genes. Therefore, the search for putative tobacco Zn transporters was performed with the use of both AWOK and NCBI databases. Tobacco metal transporters were identified based on homology to the previously annotated sequences of *Arabidopsis thaliana* genes belonging to the following major metal transport families: (i) ZIPs: ZRT, IRT-like proteins; (ii) NRAMPs: natural resistance-associated macrophage proteins; (iii) MTP: metal tolerance proteins; (iv) MRP/ABCC: multidrug resistance proteins.

The genome of *Nicotiana tabacum*, Basma Xanthi has been downloaded from http://www.ncbi.nlm.nih.gov/Traces/wgs/?\&val=AWOK01 (BX), and a search for tobacco sequences homologous to sequences of metal transporters gene from *A. thaliana* was performed. For this we used program BLASTn (NCBI Resource Coordinators, 2016) which was run using an amino acid sequence from *Arabidopsis* against the Basma Xanthi genome. We retained alignments with *e*-value not exceeding 1*e*-05. Next we used AAT package (Huang et al., 1997) for analyzing and annotating large genomic sequences containing introns. The predicted exons were further filtered in order to avoid spurious predictions (minimal length at least 10 amino acids, plus a threshold on confidence levels for both boundaries that were returned by AAT package).

In parallel, the NCBI database was used for BLASTn searches of *Nicotiana* sequences with homology to the already annotated *A. thaliana* sequences of metal transporters. FGENESH and FGENESH+ tools (Softberry, Mount Kisco, NY, United States^1^) were used to identify the untranslated regions (UTRs), exons, and introns within the scaffold containing sequences of chosen tobacco genes, and to predict putative proteins encoded by these genes. Protein sequence alignments were performed using ClustalW and the phylogenetic trees were constructed with MEGA7.0 software (Tamura et al., 2013) using the maximum likelihood method with 1000 bootstrap replicates. The prediction of membrane-spanning regions and orientation was performed using Phobius software (Käll et al., 2004).

### Identification of Metal Transport Genes Differentially Regulated in Leaves by Exposure to Zn Excess

The 5-week old tobacco plants (obtained as described in the section “Plant Material and Growth Conditions”) were grown for the next 4 days in the control medium, then they were exposed to 200 μM Zn (added to the quarter-strength Knop’s medium) for up to 3 days. Quarter-strength Knop’s medium (contains 0.5 μM Zn) served as a reference condition. On the 1st, 2nd, and 3rd day of the Zn treatment blades from the 2nd leaf (counting from the base of a plant) were collected. Leaves were cut out from each plant, petioles and the major midribs were excised, and the fragments of the blades were immediately frozen in liquid nitrogen. Three independent biological replicate experiments were performed. For each repetition, the leaf blade fragments were collected from a total of 40 plants.

Quantitative Real-Time PCR (RT-qPCR) was used to determine which putative metal transport genes out of those identified by bioinformatics analysis (see section “Database Search for Putative Tobacco Metal Transport Family Members”) are differentially regulated in the leaf blades by 200 μM Zn (as compared with the control conditions). Specific primers were designed for the sequences of identified metal transport genes from the *ZIP, NRAMP, MTP*, and *MRP/ABCC* families identified in the tobacco genome databases (Supplementary Table S1).

### Cloning of *NtZIP1-Like* and Bioinformatic Analysis

The whole sequence of *ZIP1-like* was determined by 5′- and 3′rapid amplification of cDNA ends (RACE) using SMARTer RACE 5′/3′ Kit (Clontech Laboratories, Inc. and A Takara Bio Company, Mountain View, CA, United States) according to the manufacturer’s manual. Briefly, the partial sequence of *ZIP1-like* (previously identified in the tobacco genome database at AWOK01S302253.1) was used to design gene-specific primers (GSPs) for the 5′- and 3′-RACE reactions [2253-GSP1-1-UPM (5′) 2253-GSP2-1-UPM (3′)] (Supplementary Table S1). Amplification of the 5′- and 3′-end was performed in 50 μl reactions with the use of the Phusion HF polymerase (Thermo Scientific). The PCR product of an expected size was electrophoresed on an 1% agarose/EtBr gel and excised DNA fragment was cleaned with the Macherey-Nagel PCR clean-up Gel extraction (Germany, VWR MANB740609.50) according to the manufacturer’s instruction. It was cloned into the pRACE vector (provided with the SMARTer RACE kit) and subsequently the reaction mixture was used to transform *Escherichia coli* Stellar Competent Cells. The plasmids were isolated from individual colonies, and the presence of the expected insert was confirmed by PCR screening (starters M13/For and M13/Rev), then by sequencing (Genomed, Poland). Nucleic and amino acid sequence alignments between obtained sequence and sequence predicted by Fgenesh program was performed using ClustalW.

The full length *NtZIP1-like* cDNA sequence was amplified by PCR (Supplementary Table S1), subcloned to pENTR^TM^/D-TOPO^®^ and used for *E. coli* One Shot^TM^ TOP10 (Invitrogen) transformation. The insert was sequenced to confirm the correct sequence. The sequence of the NtZIP1-like cDNA was deposited to the NCBI database (2015) under the accession number XM_016652513.

### RNA Extraction

Total RNA was extracted from samples stored in -80°C with the use of an RNeasy Plant Kit (Syngen, #SY341010) according to the manufacturer’s recommendations, followed by DNase I digestion (Qiagen, #79254). The samples of RNA were quantified at 260 nm using a Nanodrop spectrophotometer ND 100 (Nanodrop, Wilmington, DE, United States). RNA concentration and purity was determined before and after DNA digestion using a NanoDrop spectrophotometer ND-1000 (Nanodrop, Wilmington, DE, United States) and the 260/280-nm ratio showed expected values between 1.8 and 2.0. The RNA integrity of samples was also confirmed by electrophoresis in agarose gel.

### Quantitative Real-Time PCR

The cDNA used as a template for the RT-qPCR reaction was synthesized using RevertAid^TM^ First Strand cDNA Synthesis Kits (Fermentas) in a 20 μl reaction volume containing 1–3 μg of aRNA and oligo d(T)18 primers following the manufacturer’s protocol. The RT-qPCR reaction was performed according to procedures described in Kendziorek et al. (2016) with minor modifications. It was performed in a Roche mastercycler (LightCycler^®^480 System, Roche) using Light Cycler480 SYBR Green (Master 0488735001) according to the manufacturer’s recommendations. The primers (Supplementary Table S1) were designed using IDT OligoAnalyzer 3.1^2^ and OligoCalc: Oligonucleotide Properties Calculator^3^. The tobacco *NtPP2A* (*protein phosphatase 2A;* AJ007496) gene was used as the reference gene/internal control and was amplified in parallel with the target gene allowing gene expression normalization and providing quantification. Their stability in the plant samples collected for expression analysis was measured and shown in Supplementary Figure S1. Expression analysis was performed with at least three independent biological replicates. For each sample, reactions were set up in triplicate and means were calculated. Quantification of the relative transcript levels was performed using the comparative dCt (threshold cycle) method. Validation experiments were performed to test the efficiency of the target amplification and the efficiency of the reference amplification. The general quality assessment of the qPCR results was based on the amplification and melting curve profile of the samples in relation to the assay controls (non-template controls).

### Functional Analysis of NtZIP1-Like in *Saccharomyces cerevisiae* Strains

The full cDNA of NtZIP1-like was amplified using Phusion polymerase with the primers introducing *XbaI* and *BamHI* restriction sites for amplification (Supplementary Table S1). Obtained sequence was restriction ligated into the pUG35 yeast expression vector (kindly provided by Dr. M. Migocka, The University of Wrocław). The open reading frame (ORF) of NtZIP1-like was inserted in frame C-terminal to the ORF of EGFP (construct pUG35-*NtZIP1-like-EGFP*) and with the STOP codon (construct pUG35-*NtZIP1-like*), and fused with the methionine-repressible MET25 promoter (Kurat et al., 2006; Petschnigg et al., 2009). The same cloning strategy has been used to fuse the EGFP coding region to the N-terminal end of the *NtZIP1-like* in the pUG36 vector (construct pUG36-*EGFP-NtZIP1-like*). The resulting constructs and empty vectors were transformed to yeasts using the lithium acetate method (Gietz and Schiestl, 2007).

The yeast strains used in this study were DY1457 (MATa, ade1 can1 his3 leu2 trp1 ura3), the mutant ZHY3 - Δ*zrt1/zrt2* (DY1457 + zrt1::LEU2, zrt2::HIS3) defective in high and low affinity zinc uptake, and Δ*fet3fet4* (MATa trp1 ura3 Dfet3::LEU2 Dfet4::HIS3), defective in high and low affinity iron uptake system. Yeast strains were grown on liquid synthetic complete medium (SC-URA-MET/Glu) of the following composition: yeast nitrogen base supplemented with amino acids (without uracil and methionine), 2% (w/v) glucose, pH 5.3 (containing 0.2 mM Zn) overnight at 30°C with shaking. On the next day the OD_600_ was measured, adjusted to OD_600_ of approximately 0.2 and yeasts were grown for another 2–5 h. The OD_600_ was measured again, adjusted to OD = 0.3, series of dilutions were made (1.0, 0.1, 0.01, 0.001, and 0.0001) and 3 μl aliquots of each yeast culture were spotted onto plates containing (SC-URA-MET/Glu) medium solidified with 2% (w/v) agar supplemented with components depending on needs.

To determine whether Zn is a substrate for the NtZIP1-like, the Δ*zrt1/zrt2* yeast strain with the expression of pUG35, pUG35-*NtZIP1-like*, pUG35-*NtZIP1-like-EGFP*, pUG36 or pUG36-*EGFP-NtZIP1-like* and WT (DY1457) with the expression of pUG35 or pUG36 (empty vector) were grown on liquid SC-URA-MET/Glu medium (containing 0.2 mM Zn) and spotted onto the agar-solidified SC-URA-MET/Glu medium containing series of EGTA (ethylene glycol-bis(β-aminoethyl ether)-N,N,N′,N′-tetraacetic acid) concentrations: 2.5, 5.0. 7.5, and 10.0 mM. Yeast growth was monitored for the next 5 days.

To examine if Cd is a substrate for the NtZIP1-like, yeast WT (DY1457) was transformed with the pUG35, pUG35-*ZIP1-like* and pUG35-*ZIP1-like-EGFP*, and spotted onto the agar-solidified SC-URA -MET/Glu medium containing range of Cd (as CdCl_2_) concentrations (5, 10, 20, 50, and 75 μM). The sensitivity to cadmium was monitored.

To determine whether Fe is a substrate for the NtZIP1-like, complementation of the growth defect of Δ*fet3fet4* mutant line by expression of *NtZIP1-like* (the same constructs as above were used for expression) was tested on plates containing agar-solidified SC-URA-MET/Glu control medium. Moreover, modification of the sensitivity to high Fe due to expression of *NtZIP1-like* was examined on medium supplemented with 50 or 100 μM FeCl_3_.

### Subcellular Localization of NtZIP1-Like Protein

The entire cDNA sequence of the ORF of *NtZIP1-like* were obtained using Phusion polymerase and primers introducing CACC at the 5′ end of the amplicon (underlined): forward 5′ CACCATGAATAACCACAATGTCCAAGT 3′ and reverse 5′-AGCCCATTTAGCCATCACAGA -3′. The CACC overhand in the forward primer is required for directional cloning in the pENTR/D TOPO^®^ vector (add provider). Following amplification, the cDNA was ligated into a Gateway entry vector pENTR/D-TOPO (Invitrogen). Fusion proteins with GFP were produced by the recombination (LR reaction) of entry vectors pENTR/D-TOPO-*NtZIP1-like* with destination vector pMDC43 (N-terminal GFP) (Curtis and Grossniklaus, 2003) using the Gateway system (Invitrogen).

Resulting construct pMDC43-*GFP*-*ZIP1-like* was sequenced (Genomed, Poland), then used for determination of the subcellular localization of the NtZIP1-like in tobacco cells. The pMDC43-*GFP-ZIP1-like* fusion protein was transiently expressed in tobacco leaves as described by Siemianowski et al. (2013). Leaves of 6-week-old WT tobacco grown on control medium were infiltrated with *Agrobacterium tumefaciens* carrying the pMDC43-*GFP*-*ZIP1-like* construct. Three days from the infiltration, leaves were analyzed using a Nikon A1 confocal laser scanning microscope (Melville, NY, United States). GFP signals were detected by excitation with the 488 nm line of the argon laser and emission was recorded between 500 and 560 nm. To confirm plasma membrane localization of NtZIP1-like, cell walls at the plasma membrane border of examined tobacco epidermal cells were visualized by staining with the 50 μM water solution propidum iodide (20 min), a membrane-impermeant red fluorescent dye (Suh et al., 2007; McFarlane et al., 2010). Imaging was detected by 543 nm excitation and 617 nm emission. In parallel, chlorophyll autofluorescence was monitored using a HeNe (543 nm) laser for excitation.

### Hydroponic Experiments

#### Developmental Regulation of NtZIP1-Like Expression

To study the organ-specific expression of *NtZIP1-like* which depends on a developmental stage of the vegetative phase of growth the 3-week old tobacco plants were transferred from the agar plates to the control liquid medium (see section “Plant Material and Growth Conditions”) and cultivated for up to 6 weeks. For the first 3 weeks on hydroponics the nutrient solution was changed every 3–4 days (plants were grown in 2-L plastic pots, five plants per pot). Next they were transferred to 1.2-L pots (two plants per pot) for the consecutive 3 weeks, and the medium was changed every 2nd day. The plant samples were collected at three stages of vegetative development: (Stage 1) small seedlings (3 weeks at the plates and 1 week on hydroponics); (Stage 2) young plants with rosette leaves (3 weeks on plates and 3 weeks on hydroponics); (Stage 3) adult plants with formed stem (3 weeks on plates and 6 weeks on hydroponics). At the Stage 1 and Stage 2 all leaf blades and all roots were collected separately. At the Stage 3 the following organs were collected: (i) *from the aerial part of each plant* – (a) two young leaves counting from the top (length of the blade of the smallest one was 0.5 cm); (b) two oldest leaves (counting from the base); (c) stem -3 cm of the middle part; (ii) *roots*- two segments of the roots which grew out directly from the hypocotyl (adventitious roots were not included into analysis): (a) apical segment: 3–4 cm measured from the tip of the root; (b) basal segment: 3–4 cm measured from the base of the root. Plant samples were immediately frozen in the liquid nitrogen and stored in -80°C until expression analysis. Three independent biological replicate experiments were performed. For each repetition samples were collected from a total of 30 plants (for Stage 1), 15 plants (for Stage 2) and 10 plants (for Stage 3).

#### Regulation of the Expression of NtZIP1-Like by Zinc

To determine if the expression of *NtZIP1-like* depends on Zn availability, the 5-week old plants (see section “Plant material and growth conditions”) were grown for the next 4 days in the control medium, then they were subjected to the following treatments: (i) to Zn deficit (Zn was omitted from the medium) for 4 days; (ii) to Zn deficit for 4 days followed by re-supply with control conditions for 2 days; (iii) to Zn excess (50 μM Zn present in the control medium) for 1 day; (iv) control medium in parallel to all treatments. At the end of each experiment plant material was collected, frozen in liquid nitrogen, and stored in -80°C for expression analysis. The following organs were collected: (i) the blades of the 2nd and 3rd leaf (counting from the base) without petioles and the major midribs; (ii) two sectors of the roots which grew out directly from the hypocotyl (adventitious roots were not included into analysis): (a) 3–4 cm of the apical region; (b) 3–4 cm of the basal region. Three independent biological replicate experiments were performed. Samples were collected from a total of 10 plants for each repetition.

### Statistical Analysis

All presented data are from one experiment that is representative of three to four independent replicate experiments. Statistical significance was evaluated at the 0.05 probability level using Student’s *t*-test.

## Results

### Bioinformatic Analysis of Transporter Families in Tobacco

*Arabidopsis thaliana* cDNAs from major metal transport families were used as the query sequences to identify genes encoding Zn transporters in tobacco. By screening the tobacco genome scaffolds, sequences of tobacco genes that significantly matched with the query cDNAs (query coverage > 80%) were selected. The following protein families from *A. thaliana* were included in the search: (i) ZIPs: ZRT, IRT-like proteins; (ii) NRAMPs: natural resistance-associated macrophage proteins; (iii) MTPs: metal tolerance proteins; (iv) MRP/ABCC: multidrug resistance proteins. For each gene from *A. thaliana* used as a query, several tobacco homologous sequences were identified on different scaffolds. These sequences were screened for exon orientation, start and end positions, and confidence scores for the boundaries (Supplementary Table S2). Further the selected tobacco scaffolds were screened to identify full putative genomic sequences of *NtZIP*, *NtNRAMP*, *NtMTP*, and *NtMRP* (*ABCC*) genes, including transcription start sites, exons, introns, and polyadenylation sites using the FGENESH tool (Salamov and Solovyev, 2000), whereas Phobius system based on a hidden Markov model (HMM) approach, was applied to predict membrane topology of NtZIP1-like protein. The list of genomic sequences comprises twenty-one newly identified tobacco putative metal transporters. Their names were given according to the NCBI terminology of the genes from *A. thaliana*, which were used as a query (Supplementary Figure S2). Identified sequences (Supplementary Figure S2) were used to design primer pairs (Supplementary Table S1) for determination of their transcript level in the leaf blades of tobacco plants exposed to high Zn.

### Response of Genes from the ZIP, NRAMP, MTP, and MRP/ABCC Families in Tobacco Leaves to High Zn

**FIGURE 1:**
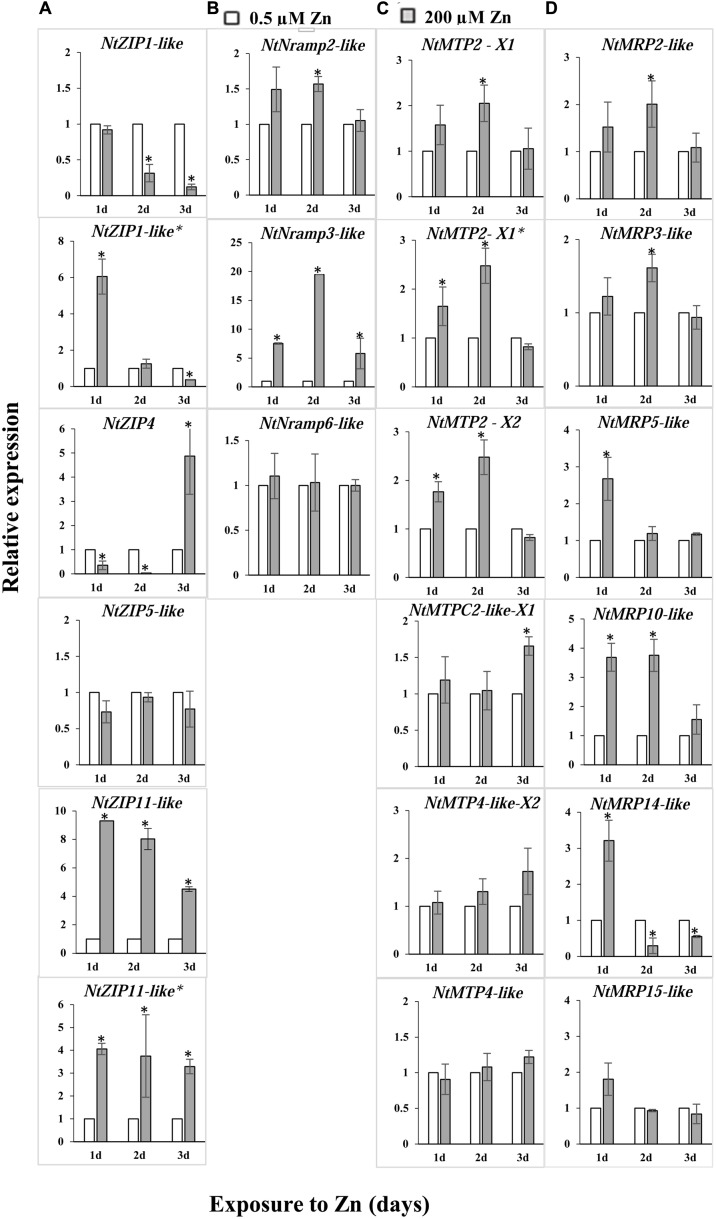
Expression of metal transport genes in the leaves of *Nicotiana tabacum* plants grown under control conditions (white bars) and at 200 μM Zn (gray bars). RT-qPCR was used to determine transcript levels. Genes from four metal transport families were included in the analysis: *NtZIPs* **(A)**, *NtNRAMPs* **(B)**, *NtMTPs* **(C)**, and *NtMRPs/NtABCCs* **(D)**. The 5.5-week-old plants were grown for up to 3 days in the presence of 200 μM Zn added to the control medium (0.5 μM Zn), and in parallel in the control medium. The average transcript levels are presented for three technical replicates. Gene expression was normalized to the *PP2A* level. Expression under control conditions was set to 1 as the frame of reference within each experiment. Values correspond to means ± SD (*n* = 3); those significantly different from the control (Student’s *t*-test) are indicated by an asterisk (*P* ≤ 0.05).

To determine which of the identified metal transporters could be potentially involved in the regulation of Zn in tobacco leaves, their expression in the blades of plants grown in the presence of 200 μM Zn for up to 3 days was compared to the control conditions (**Figure 1**). From the ZIP family, three genes were identified with a several-fold difference in the transcript level between the Zn-exposed plants relative to those grown at the control medium. The most significant change was noted for *NtZIP1-like* and *NtZIP4* (downregulation) and *NtZIP11* (upregulation). Within the *NtNRAMP*s elevated expression was detected for a putative transporter *NtNRAMP3-like*. Moreover, modified expression was noted for three isoforms of *NtMTP2*. Out of identified six putative *MRP/ABCC* transporters which were subjected to analysis, the expression of three of them (*NtMRP10-like* and at a lower level *NtMRP5-like* and *NtMRP14-like)* were modified by high Zn.

### Phylogenetic Relationship of *NtZIP1-Like* from Tobacco

In this study, the focus was on finding genes potentially involved in the accumulation of Zn in tobacco leaves, which respond to high Zn. The ZIP family proteins are considered as major Zn uptake transporters (Ricachenevsky et al., 2015). Based on downregulation of *NtZIP1-like* by high Zn in leaves (**Figure 1**), the assumption was made that it plays a role in Zn influx into the cytosol. Therefore, the *NtZIP1-like* was chosen for cloning and characterization.

**FIGURE 2:**
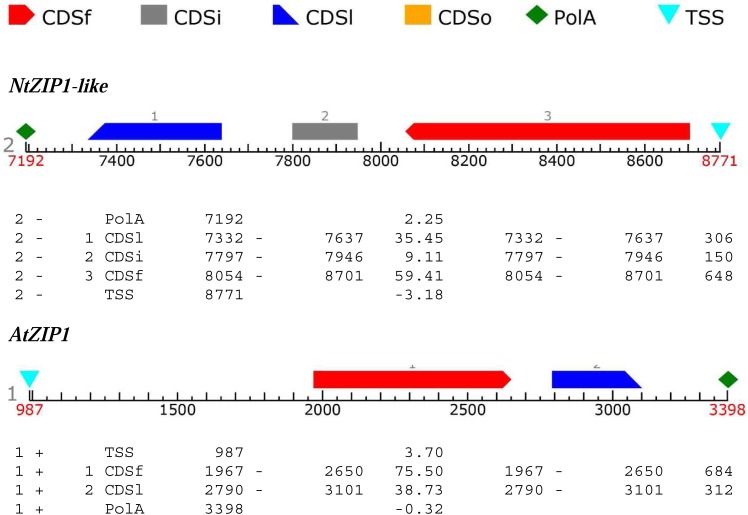
Prediction of exons and introns organization in *NtZIP1-like* and *AtZIP1*. FGENESH tools were used to identify ORF sequence with Start Side of Transcription (TSS), sizes/length/position of introns and exons in the regions of the CoDing Sequences (CDS; CDSf- First CoDing Sequence, CDSi- Inner CoDing Sequence, CDSl- Last CoDing Sequence) and poliA region (polA).

**FIGURE 3:**
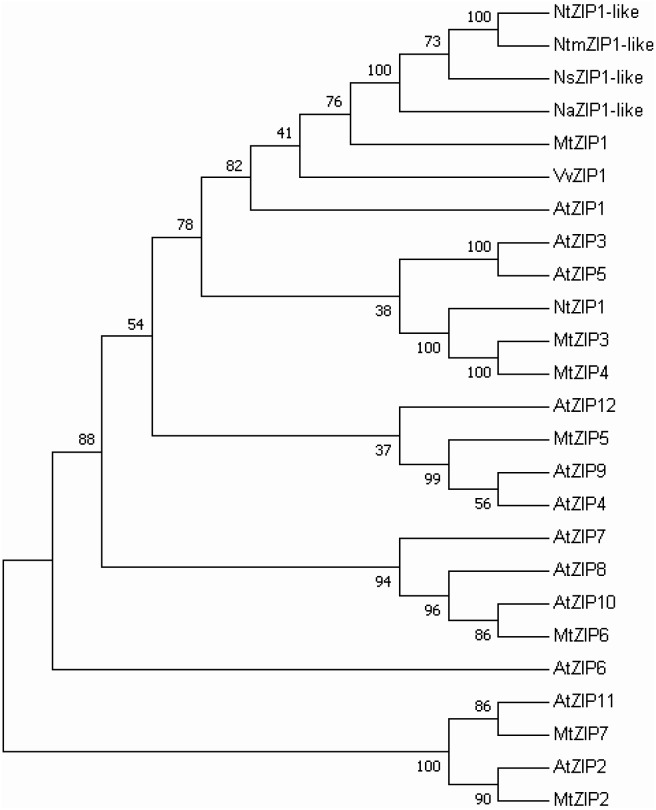
Phylogenetic analysis of ZIP1 transporters from selected species. The unrooted tree was constructed based on amino acid sequences identified in the Aramemnon (*Arabidopsis thaliana*) and NCBI database (*Nicotiana* species, *Medicago truncatula*, *Vitis vinifera*), using the MEGA 7.0 software. The lengths of branches are proportional to the degree of divergence. Numbers in the figure represent bootstrap values (1000 replicates). The accession numbers are as follows: *Arabidopsis thaliana*, AtZIP1 - At3g12750.1, AtZIP2 - At5g59520.1, AtZIP3 - At2g32270.1, AtZIP4 - At1g10970.1, AtZIP5 - At1g05300.1, AtZIP6 - At2g30080.1, AtZIP7 - At2g04032.1, AtZIP8 - At5g45105.1, AtZIP9 - At4g33020.1, AtZIP10 - At1g31260.1, AtZIP11 - At1g55910.1, AtZIP12 - At5g62160.1; *Nicotiana attenuata*, NaZIP1-like XP_019256554; *Nicotiana tabacum*, NtZIP1-like XP_016507999, NtZIP1- NP_001312674; *Nicotiana sylvestris*: NsZIP1-like XP_009772024; *Nicotiana tomentosiformis*, NtomZIP1-like XP_009608181; *Medicago truncatula*: MtZIP1 - AAR08412.1, MtZIP2 - AAG09635, MtZIP3 - AY339055, MtZIP4 - AY339056, MtZIP5 - AY339057, MtZIP6 - AY339058, MtZIP7 - AY339059; *Vitis vinifera*, VvZIP1 - XP_002264603.2.

**FIGURE 4:**
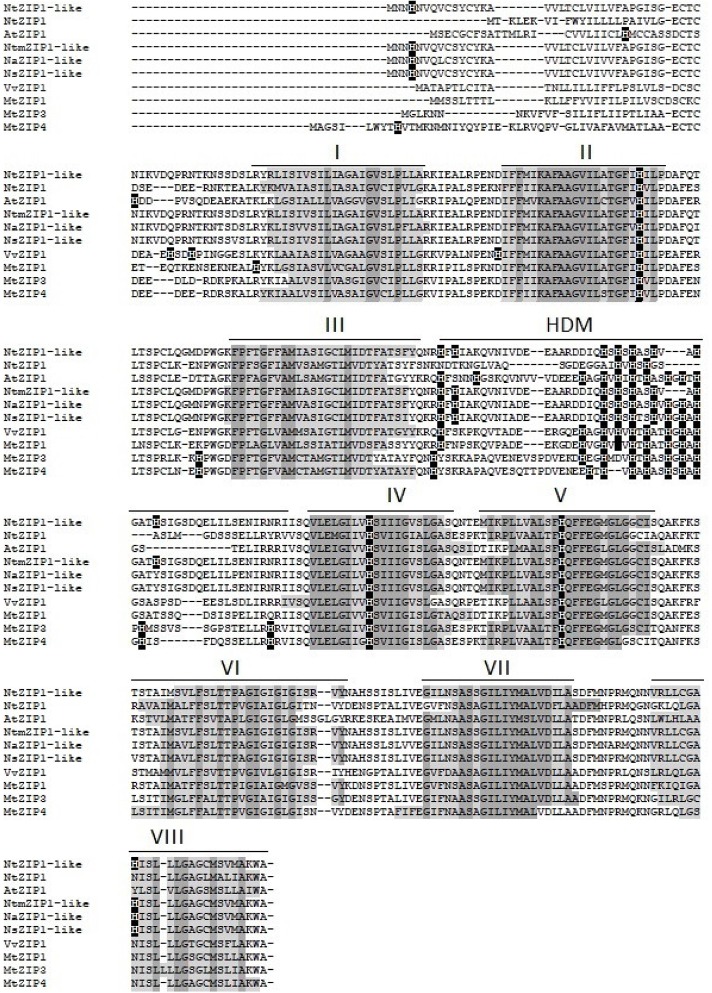
Amino acid alignment of predicted ZIP proteins from different species. Sequences were aligned using ClustalW. The prediction of membrane-spanning regions was performed using Phobius software and indicated as lines above the sequences, and numbered I–VIII, respectively. The identical amino acids are indicated with dark gray. HDM indicates the histidine rich domain within the variable cytosolic region. Dashes indicate gaps.

The ORF of the new tobacco ZIP family member – *NtZIP1-like*, consists of 1104 bp (**Table 1**) with 3 exons (**Figure 2**), and according to the prediction made by the program Fgenesh encodes 367 amino acids. To define the evolutionary relationship between ZIP1-like and the ZIP1 proteins from other organisms, as well as the other ZIPs, a phylogenetic tree was constructed (**Figure 3**). It included ZIP proteins from three species of tobacco (NtmZIP1-like, NsZIP1-like and NaZIP1-like), from *A. thaliana*, *M. truncatula*, and *V. vinifera*. It shows that NtZIP1-like is most closely related to ZIP1 proteins from other organisms including tobacco (NtmZIP1-like, NsZIP1-like and NaZIP1-like), *Medicago truncatula* (MtZIP1), *Vitis vinifera* (VvZIP1), and *A. thaliana* (AtZIP1). Within all ZIP1 sequences under comparison, NtZIP1 (Sano et al., 2012) formed a distinct clade with MtZIP3 and MtZIP4 from *M. truncatula*. The alignment of protein sequences defined at the phylogenetic tree as the closest homologs showed that the structure of NtZIP1-like is in agreement with the structure of other ZIP family members (Grotz and Guerinot, 2006). It contains eight transmembrane domains (TMs), a longer N-terminal region, a very short C tail, and a cytosolic variable region between TM domains III and IV (**Figure 4**). Histidine residues in the TMs II, IV, and V are highly conserved throughout the entire ZIP family. Our sequence analysis shows that NtZIP1-like exhibits high amino acid sequence similarity with AtZIP1 and other known ZIP family members within these three mentioned TM domains (**Figure 4**). Among them, amino acid sequence conservation within the signature region in the fourth TM domain was found. It contains consensus sequences (including a fully conserved histidine residue). On the other hand, a potential metal-binding motif containing multiple histidine residues present in the variable region between TM III and IV, differs between examined proteins primarily in the number of his residues and their localization. In this region, eight histidine residues were found in NtZIP1-like compared to nine present for example in AtZIP1, and only three in NtZIP1 (**Figure 4**). The NtZIP1 protein (Sano et al., 2012) formed also a separate clade containing AtZIP3 and MtZIP3, MtZIP4 and AtZIP5.

**Table 1:** Sequence identity between the NtZIP1-like and ZIP1 predicted proteins from selected species (sequences were chosen based on phylogenetic tree given in **Figure 3**).

Name of gene	Accession no.	No. of amino acid	Identity of *NtZIP1-like* (%) (aa)	Length of gene (bp)	Identity of *NtZIP1-like* (%) (bp)
*NtZIP1-like*	XM_016652513	367	–	1104	–
*AtZIP1*	At3g12750	355	56	1068	58
*NtmZIP1-like*	XP_009608181	367	100	1104	100
*NaZIP1-like*	XM_019401009	370	94	1113	96
*NsZIP1-like*	XM_009773722	370	95	1113	97
*MtZIP1*	AAR08412	358	59	1077	67
*VvZIP1*	XP_002264603	360	57	1083	66
*NtZIP1*	NM_001325745	339	54	1020	62

The NtZIP1-like shares 56 and 54% identity at the amino acid level with AtZIP1 and NtZIP1, respectively, whereas 58 and 62% at the nucleotide level. The highest homology was found between the NtZIP1-like and other three tested tobacco ZIP1 proteins such as NtnZIP1-like (100%), NaZIP1-like (94 and 96%) and NsZIP1-like (95 and 97%, respectively (**Table 1**).

### NtZIP1-Like Localizes to the Plasma Membrane

To gain insight into the functioning of NtZIP1-like, its subcellular localization was determined by transient expression of the NtZIP1-like protein fused to the N terminus of green fluorescent protein (GFP) under the control of the cauliflower mosaic virus (CaMV) 35S promoter in tobacco leaves.

**FIGURE 5:**
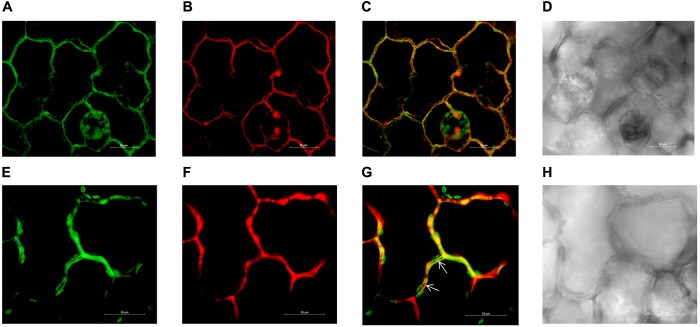
Plasma membrane localization of the NtZIP1-like-GFP fusion protein transiently expressed in tobacco leaf epidermis. Confocal images of two areas of *NtZIP1-like-GFP* expressing epidermal cells **(A–D; E–H)**. Sections were labeled with propidium iodide. GFP fluorescence concentrated to the plasma membrane **(A,E)**; propidium iodide staining red of the same cells follows their contours **(B,F)**; overlapped GFP and propidium iodide signal **(C,G)**; bright field **(D,H)**. Locally, signal from the cell wall (stained red) clearly visible between adjacent GFP-labeled plasma membranes (arrows).

Three days after the infiltration of the leaves with *Agrobacterium* expressing pMDC43-*GFP*-*ZIP1-like*, the GFP signal (green fluorescence) was detected in tobacco epidermal cells along the cell walls indicating localization of NtZIP1-like protein at the plasma membrane (**Figure 5**). Cell walls were stained with propidium iodide and the green fluorescence (**Figure 5A**) coincided with the red signal, derived from propidium iodide (**Figures 5B,C**). The co-localization of the GFP-derived signal and propidium iodide staining of the cell walls indicates localization of GFP-fused NtZIP1-like protein at the plasma membrane (Pighin et al., 2004; Lee et al., 2010; Siemianowski et al., 2013). Moreover, at higher magnification the signal from the cell wall (red) and from the GFP-labeled plasma membrane (green) were separated (indicated by arrows; **Figures 5E–G**). Altogether, results support the conclusion that NtZIP1-like is a plasma membrane protein.

### Yeast Complementation Supports a Role for NtZIP1-Like as a Zn Transporter

**FIGURE 6:**
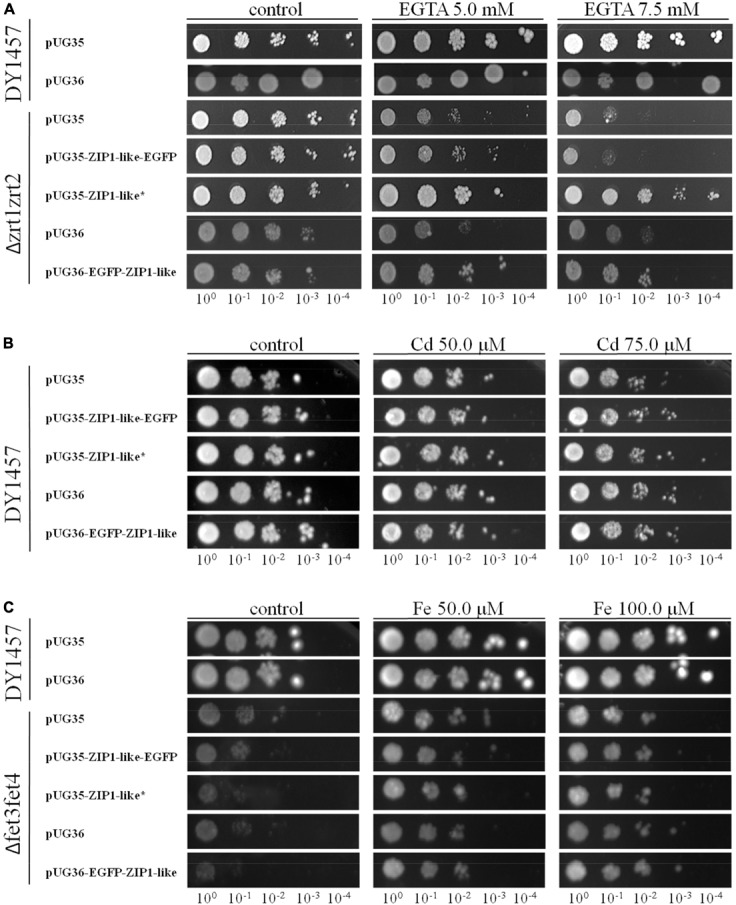
Complementation by *NtZIP1-like* cDNA of yeast mutants defective in metal uptake on selective media. Yeast cells: DY1457, Δ*zrt1zrt2* (defective in Zn uptake), Δ*fet3fet4* (defective in Fe uptake) were transformed with empty vectors pUG35/36 as a control, or with vectors carrying tobacco gene *NtZIP1-like* with (^∗^) or without a stop codon. Yeast cultures were adjusted to an OD600 of 0.2, and 3 μl of serial dilutions (from left to right in each panel) was spotted on SC-URA-MET medium supplemented with EGTA **(A)**, CdCl_2_ **(B)** or FeCl_3_ **(C)** or 0.2 μM Zn (control). The plates were incubated for 3–6 d at 30°C. The images are representative of three independent experiments.

Yeast functional complementation was used to determine the capacity of NtZIP1-like to transport Zn. The yeast *zrt1zrt2* double mutant (ZHY3) defective in high and low affinity Zn uptake was used (Eide, 2003). The expression of full-length cDNA of *NtZIP1*-*like* gene fused with the *eGFP* at its C-terminal end (construct pUG35EGFP-*NtZIP1*-*like-EGFP*), as well as at its N-terminal end (construct pUG36-*EGFP-NtZIP1-like*) did not complement the growth defect of the Δ*zrt1zrt2* yeast mutant (**Figure 6A**). In contrast, the expression of the construct pUG35-*NtZIP1-like* (with the STOP codon) fully restored growth under Zn-limited conditions (**Figure 6A**). These result indicates that NtZIP1-like is a plasma membrane protein mediating Zn uptake. The lack of rescue by constructs containing *EGFP* both at the C- and N-terminal end suggests that the presence of the eGFP protein makes the NtZIP1-like protein dysfunctional.

Some ZIP proteins mediate transport of Cd (Ramesh et al., 2003; Nakanishi et al., 2006; Stephens et al., 2011). To determine if NtZIP1-like is a Cd uptake protein, the wild-type yeast line DY1457 was transformed with pUG35-*NtZIP1-like* (with the STOP codon), and pUG35-*NtZIP1*-*like-EGFP*. If the NtZIP1-like is involved in Cd influx, the yeast transformants should be more sensitive to this metal. As shown in **Figure 6B** the growth of the wild-type transformed with the empty vector or with both tested constructs was limited by a range of Cd present in the medium to the same extent indicating no Cd transport capacity by NtZIP1-like.

Finally, to study the capacity of the NtZIP1-like to transport Fe, a yeast mutant Δ*fet3fet4* defective in both high- and low affinity Fe uptake systems was transformed with the *NtZIP1-like* cDNA to examine if it complements the defect in Fe transport. As shown in **Figure 6C**, expression of *NtZIP1-like* in the Δ*fet3fet4* did not restore the growth of mutants at control conditions, and it did not modify the sensitivity of yeast to Fe excess. To conclude, the results indicate that the NtZIP1-like does not transport Fe.

### Developmental Regulation of *NtZIP1*-*Like*

**FIGURE 7:**
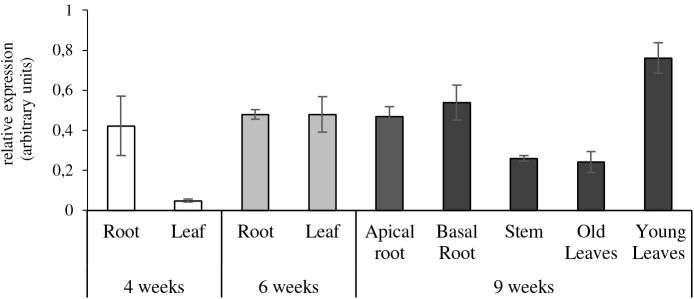
Organ- and developmental-regulation expression of *NtZIP1-like* in *N. tabacum*. Analysis was performed by RT-qPCR. Plants were grown at control conditions. Transcript levels were monitored in 4-week-old plants (whole roots and leaves); 6-week-old plants (whole roots and leaves), and 9-week-old plants (apical and basal segments of roots, stems, young leaves, old leaves). The level of *NtZIP1-like* transcript was normalized to *PP2A* expression level.

Our study showed that *NtZIP1-like* is expressed both in the roots, leaves and stems, but the level depends on the developmental stage (**Figure 7**). The transcript level in the leaves was very low in young 4-week old plants compared to a 6-fold increase in 6-week old tobacco. In the adult 9-week old plants its expression in young leaves was 3-times higher than in the old ones. *NtZIP1-like* was expressed at a moderate level in stems. In the roots of young 4-week old plants the transcript level was very low, and a 6-fold increase was detected in 6-week old tobacco. It remained at that level in adult 9-week old plants and did not differ in the apical and basal part of the root.

### Expression of *NtZIP1*-Like Is Zn Regulated

**FIGURE 8:**
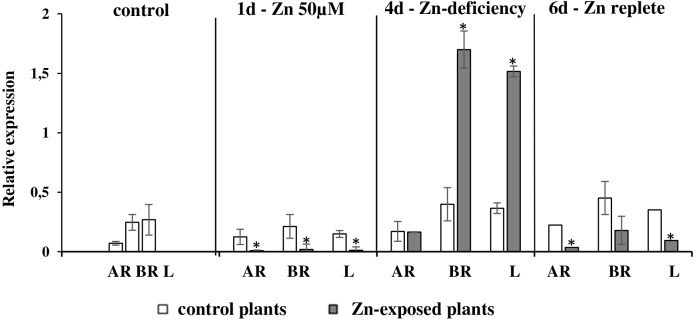
Expression pattern of *NtZIP1-like* in *N. tabacum* under various Zn conditions. Plants were grown in standard nutrient solution (control) and then transferred into modified control media: supplemented with 50 μM Zn for 1 day (1d); without Zn for 4 days (4d - Zn deficiency); plants grown at Zn-deficiency conditions for 4 days were transferred to the control medium for 2 days (6d - Zn replete). RT-qPCR analyses was performed on cDNA prepared from leaves (L), apical part of roots (AR) and basal part of roots (BR) of *N. tabacum*. Gene expression was normalized to the *PP2A* level. Values correspond to means ± SD (*n* = 3); those significantly different are indicated by an asterisk (*P* ≤ 0.05).

It has been shown that Zn is a substrate for NtZIP1-like. To know more about the possible physiological role of NtZIP1-like in tobacco, its expression was analyzed in the roots, leaves and stems of plants exposed to Zn excess (50 μM for 1 day), and to Zn-deficiency (no Zn for 4 days) subsequently followed by a replete conditions (4-day Zn deficit followed by 2 days of control conditions). In agreement with downregulation in the leaf blades by 200 μM Zn (**Figure 1**), downregulation by 1-day exposure to 50 μM Zn was detected in leaves, and in the roots (both in the apical and basal segment) (**Figure 8**). Interestingly, its expression was highly upregulated by Zn-deficiency in the leaves and in the basal segment of the roots. No response to low Zn in the medium was noted in the apical part of the root considered as primarily responsible for Zn uptake (**Figure 8**).

## Discussion

Although tobacco, as a plant with a high capacity to accumulate large amount of metals (including Zn and Cd) in leaves, is used for phytoremediation of metal contaminated soil (Herzig et al., 2003, 2014; Lugon-Moulin et al., 2004; Dguimi et al., 2009; Vangronsveld et al., 2009), metal transporters involved in uptake and storage of metals in leaf tissues remain unknown. Here, based on bioinformatics searches for tobacco metal transporter sequences (Supplementary Figure S2 and Supplementary Table S2), and subsequent analysis of the regulation of the candidate genes by Zn excess (200 μM Zn) in leaves, ten genes (out of twenty-one tested) with significantly modified expression were identified (**Figure 1**). They represent putative metal transport genes that likely contribute to the storage of Zn excess in tobacco leaves, and include transporters involved in sequestration, redistribution and uptake of metals.

In sequestration of Zn in tobacco leaves exposed to 200 μM Zn three isoforms of *NtMTP2* may play a role. Elevated expression of two isoforms of *NtMTP2*-*X1* and *NtMTP2*-*X2* (**Figure 1C**) suggests a likely involvement in loading of Zn into vacuoles, which are the major storage compartments within cells. The MTP2 proteins are not fully characterized so far in plants. It is known that the MTP2 belongs to the Group 1 of MTP vacuolar Zn transporters. Phylogenetic analysis showed that MTP1, MTP2, and MTP3 originate from a common MTP1/2/3 ancestors (Gustin et al., 2011).

The concentration of a metal in vacuoles depends not only on efficient loading, but also on the rate of mobilizing vacuolar pool back to the cytosol, which, among others, is under control of NRAMP proteins. In tobacco leaves expression of *NtNRAMP3-like* was significantly induced by high Zn supply (**Figure 1B**). Oomen et al. (2009) showed that *TcNRAMP3/4* (and to a lesser extent *AtNRAMP3/4*) expression is regulated by Zn supply (low-efficient-excess), though the pattern of the regulation has not been fully established. However, until now Zn has not been shown to be a substrate for NRAMP3. The AtNRAMP3 from *A. thaliana* and TcNRAMP3 from *T. caerulescens* mediate efflux of Fe, Cd, and Mn from vacuoles to the cytoplasm (Thomine et al., 2003; Oomen et al., 2009). Ability to transport not only Fe, Mn, Cd but also Zn was shown for AtNRAMP4 and TcNRAMP4 only (Oomen et al., 2009). Thus, future studies will show whether the NtNRAMP3-like is localized to the tonoplast (like AtNRAMP3 or TcNRAMP3) or to the plasma membrane (like e.g., OsNRAMP3; Yamaji et al., 2013), what the substrates are, and what its role in the accumulation of high amounts of Zn in tobacco leaves.

The next identified new putative metal transporters regulated upon high Zn concentration in tobacco leaves are from the MRP/ABCC family. The major changes were found for the *NtMRP10-like* and *NtMRP14-like*, whereas to a lesser extent for *NtMRP2-like, NtMRP3-like and NtMRP5-like* (**Figure 1D**). The MRP/ABCC proteins carry various xenobiotics including metal complexes. Until now, there are only a few studies on plants indicating involvement of MRP/ABCC proteins in the transport of metals as conjugates to various substrates (Klein et al., 2006). Heterologous expression of *AtMRP7* in tobacco has suggested a role in Cd transport into the root vacuoles (Wojas et al., 2009). AtABCC1 and AtABCC2 were shown to be targeted to the tonoplast and mediated the vacuolar sequestration of phytochelatin (PC) complexes with Cd(II) and Hg(II) (Park et al., 2012). The MRP/ABCC genes have been shown to be regulated by metals. For example, the expression of *AtMRP3* is induced by Cd, Ni, As, Co, and Pb, but not Zn or Fe (Bovet et al., 2003; Zientara et al., 2009). Upregulation by Cd was also confirmed for *AtMRP6* (Gaillard et al., 2008) and *AtMRP7* (Bovet et al., 2003), and by high Zn for *TcMRP10* in the roots and shoots of Zn hyperaccumulator *T. caerulescens* (Hassinen et al., 2007). Identification in tobacco leaves of such Zn-responsive MRP/ABCC genes is important for future study on the regulation of Zn homeostasis upon treatment with high Zn.

The emphasis in this study was to shed more light on the regulation of Zn acquisition by cells in the leaves. The ZIP proteins constitute a major Zn uptake system (Sinclair and Krämer, 2012). Here, the *NtZIP1-like* was cloned and characterized to better understand its function in tobacco.

*NtZIP1-like* contains an ORF of 1104 bp, encoding a predicted protein of 367 amino acids (**Table 1**). Phylogenetic analysis of the ZIP family proteins shows that the NtZIP1-like forms a distinct clade with other ZIP1 proteins from three tobacco species (NatmZIP1-like, NsZIP1-like, and NaZIP1-like), *A. thaliana*, *M. truncatula* and *V. vinifera* (**Figure 3**). Sequence comparisons (**Figure 4**) showed that the deduced NtZIP1-like protein shares all the basic characteristic features of members of the ZIP family of metal transporters. It has eight TM domains, a long N-terminal end, a very short C tail, and a cytoplasmic variable region between TM III and IV (**Figure 4**). The variable region contains a histidine rich domain (HRD) with the motif (HX)_n_ (*n* = 2, 3, and 4) which has been proposed as a metal binding site. The characteristic feature of ZIP proteins is the presence of a signature motif within the TM IV, and highly conserved histidine residues in TM domains II, IV, and V (Eide et al., 1996; Eng et al., 1998; Grotz et al., 1998; Guerinot, 2000; Rogers et al., 2000; Gaither and Eide, 2001). They all are present in the NtZIP1-like (**Figure 4**).

Analysis showed that two tobacco ZIP1 proteins – newly cloned NtZIP1-like and NtZIP1 (Sano et al., 2012), do not cluster together (**Figure 3**). They share 54% identity at the amino acid level (**Table 1**). Comparison of NtZIP1-like and NtZIP1 amino acids sequences (**Figure 4**) showed that an important difference between them lies within a variable cytoplasmic HRD region between the TM III and IV. Although this region displays low sequence conservation among ZIP metal transporters, most of them contain the motif (HX)_n_ (*n* = 2, 3, and 4), which has been proposed as a metal binding site (Eide et al., 1996; Eng et al., 1998; Grotz et al., 1998). Only three (HX) repetitions were detected in NtZIP1, whereas eight in the newly cloned NtZIP1-like. To compare, other ZIP1 proteins contain eight or nine (HX) repetitions. The exact function of the loop between TM III-IV is yet to be determined, however, a study by Nishida et al. (2008) on the TjZNT1 ZIP transporter from *Thlaspi japonica* showed that deletion of a part of the HRD region containing his residues localized closer to the TM IV (HRD, position 207–217 aa) abolished Zn transport ability. Hence a difference in the structure at the amino acid level might contribute to a different substrate specificity. NtZIP1 and NtZIP1-like do seem to have different substrate specificities. The expression of *NtZIP1* in yeast significantly enhanced Fe accumulation suggesting Fe uptake activity (Sano et al., 2012). In contrast, the *NtZIP1-like* failed to alter the Fe-limited growth defect of *fet3fet4* yeast mutant indicating it may not transport Fe (**Figure 6C**). NtZIP1-like is also unlikely to mediate Cd uptake since its expression in WT yeast did not modify the sensitivity to Cd (**Figure 6B**). Functional complementation of the *zrt1zrt2* mutant, defective in Zn uptake supports its potential role as a Zn transporter (**Figure 6A**). NtZIP1-like localizes to the plasma membrane when transiently expressed in tobacco (**Figure 5**). Therefore, our results indicate that NtZIP1-like is a tobacco ZIP1 uptake protein for Zn, but not for Cd or Fe. In general, an ability of ZIP1 proteins to transport Fe was shown for PtZIP1 (Fu et al., 2017). More Fe transporters were identified among other ZIP proteins for example ZmZIP2-8, OsZIP5 and OsZIP8 (Li et al., 2013), MtZIP3, 5, 6 (López-Millán et al., 2004), PtZIP7 (Fu et al., 2017) and NtZIP1 (Sano et al., 2012).

Expression of the *NtZIP1-like* was detected in all plant organs, which suggests rather a universal role in maintaining Zn homeostasis (**Figure 7**). Its role seems to be more pronounced at later developmental stages. The transcript level is greater in older plants, especially in younger leaves (as compared with the older ones) suggesting a contribution to supplying cells in developing organs with Zn. Analysis of the regulation of the *NtZIP1-like* expression by Zn availability showed upregulation by Zn-deficiency in the roots and leaves (**Figure 8**), which was similar to *AtZIP1* and *OsZIP1* (Ramesh et al., 2003; Milner et al., 2013). Interestingly, in the roots upregulation of *NtZIP1-like* was limited to the basal segment of the root only, and was not detected in the apical part. It is known that the young, apical segment of the root is responsible for acquisition of nutrients, however, not much is known about the role of the older, basal region. Our studies clearly indicate that *NtZIP1-like* is a Zn-deficiency inducible Zn uptake transporter in leaves and in roots (though in roots the induction takes place only in the basal part; **Figure 8**). Further research is needed to demonstrate the *NtZIP1-like* tissue-specific expression and regulation, as it is not clear if it is involved in Zn acquisition from the medium, or in internal uptake. Distinct regulation of ZIP genes in a different root sectors has been shown also in rice (Ishimaru et al., 2005). Expression of *OsZIP4* was detected in the meristematic region of the Zn-deficient roots. Similarly, *AtZIP2p::GUS* expression analysis revealed higher induction in the younger region of the roots grown under nutrient-replete conditions, as compared to a lower induction nearer the mature part at the root-shoot junction (Weber et al., 2004). In general, it is known that upregulation upon Zn deficiency conditions and downregulation in replete medium is ascribed to genes involved in the acquisition of micronutrients (Sinclair and Krämer, 2012), and these two features are characteristic for *NtZIP1-like* (**Figure 8**).

It is known that in the leaves of tobacco plants exposed to Zn excess, the metal is not distributed equally throughout the mesophyll cells. Instead, high concentrations were found in clusters of adjacent cells (Zn-accumulating cells) in contrast to its low level in neighboring non-accumulating ones (Siemianowski et al., 2013). Distinct expression patterns of Zn transport genes must underlie such different Zn uptake and accumulation capacity. Knowing this, we searched for genes differentially regulated in the leaves by high Zn. The *NtZIP1-like* was identified initially as downregulated by 200 μM Zn (**Figure 1**), and confirmed later as downregulated by 50 μM Zn (**Figure 8**). We hypothesize that the downregulation observed in leaves upon Zn excess could be a part of the molecular mechanism occurring in the low Zn-accumulating cells that prevents them from excessive uptake of Zn. Further comparative studies on the regulation of *NtZIP1-like* expression in leaves at low and high Zn at the cellular and tissue level are necessary in the future to investigate this.

## Conclusion

The bioinformatics analysis using information from the tobacco genome and the detailed expression study has led to the identification of ten new tobacco putative transporters involved in the regulation of Zn accumulation in tobacco leaves. They belong to different major families of metal transporters (ZIP, NRAMP, MTP, and MRP/ABCC), and undergo differential regulation in the leaves of tobacco plants exposed to 200 μM Zn. The upregulation of *NtZIP11-like*, *NtNRAMP3*, three isoforms of *NtMTP2*, three MRP/ABCC genes such as *NtMRP10-like*, *NtMRP5-like* and *NtMRP14-like*, and downregulation of *NtZIP1-like* and *NtZIP4*, indicate their contribution to a range of processes underlying uptake, sequestration and redistribution of metals in the cells and tissues. These data provide an important input for further research on metal homeostasis mechanisms in tobacco, the species used for phytoremediation of metal contaminated soil.

The detailed study on the newly cloned *NtZIP1-like* showed that the encoded protein is localized to the plasma membrane and mediates uptake of Zn, but not Fe or Cd. It is expressed in the roots and leaves – but the level of the transcript depends on the developmental stage. It is also regulated by the availability of Zn, being highly up-regulated by Zn-deficiency specifically in the leaves and in the basal part of the root but not in the apical zone. We have shown previously that tobacco mesophyll cells have a distinct capacity to store Zn in the “Zn-accumulating cells” which are next to non-accumulating ones in the leaf blade (Siemianowski et al., 2013). Our detailed studies on the *NtZIP1-like* suggest that it might be a candidate gene involved in the restriction of Zn uptake by the mesophyll cells with low capacity to accumulate Zn.

## Author Contributions

AP carried out all experiments. KK was involved in yeast study, cloning and expression analysis. MK was involved in cloning, expression analysis and hydroponic experiments. AB contributed to expression analysis. MP was involved in bioinformatics analysis and hydroponic experiments. JT performed bioinformatics analysis. BP contributed to confocal analysis. LW supervised yeast complementation assays. DA designed the study concept, coordinated the research and supervised experiments, performed data analysis, and wrote the manuscript. All authors read and approved the final manuscript.

## Conflict of Interest Statement

The authors declare that the research was conducted in the absence of any commercial or financial relationships that could be construed as a potential conflict of interest.

## Footnotes

1 http://www.softberry.com

2 http://eu.idtdna.com/calc/analyzer

3 http://www.basic.northwestern.edu/biotools/oligocalc.html
