# Supplementary material for: Contribution of NtZIP1-Like to the Regulation of Zn Homeostasis
Source: Front Plant Sci. 2018 Feb 16;9:185. doi: 10.3389/fpls.2018.00185 (PMC5820362; doi:10.3389/fpls.2018.00185)
Supplement: Supplementary file 1 [file Table_1.PDF]

Supplementary Table S1: The list of tobacco genes retrieved from assembly of the *Nicotiana tabacum* genome, and primer sequences used for expression analysis, Race PCR reactions and cloning. The names of genes were designated according to NCBI terminology. Their sequences are given in Supplementary Figure S1.

**Gene name**	**Accession no. of scaffold**	**Gene ID in GenBank**	**Primer FOR**		**Primer REV**		
			**Name**	**Sequence**	**Name**	**Sequence**	
**EXPRESSION ANALYSIS**							**Primers efficiency**
PP2A	-	AJ007496	PP2A_*for*	GCACATTCATTCAGTTTGAACC	PP2A_*rev*	GTAGCATATAAAGCAGTCAGC	1.940
NtMTP2-X1	AWOK01S500825	XM_016593653	Nt0825_for	AGA CGG CGA GAG GAT TTT CC	Nt0825_rev	ACG ACA ACA ATG CCA CTC CA	1.756
*NtMTP2-X1**	AWOK01S048943	XM_016603324	Nt8943_for	TCGGAGATTCGCATTTTCA	Nt8943_rev	GCC TCG AAA AAC CGA TGA CG	1.997
NtMTPC2-like-X1	AWOK01S314055	XR_001649367	Nt4055_for	AGAATGCTGAAGTCTTATGTTTGGGACT	Nt4055_rev	CAC GAG ATG AAA CCT GTC T	1.968
NtMTP2-X2	AWOK01S036376	XM_016593654	Nt6376_for	GAT GTT ATT GTT GAG GTT GAC CC	Nt6376_rev	TCT TGG GGG CCA CAC ATT G	1.933
NtMTP4-like-X2	AWOK01S262320	XM_016623041	Nt2320_for	CGA CGT TGG ATT CAT TAC TTG ATC	Nt2320_rev	CAC AGC CTG CAC TAA GAC TTG	1.971
NtMTP4-like	AWOK01S035670	XM_016626487	Nt5670_for	ATG AAC TTT TCT GGT TGT ATG CTA	Nt5670_rev	TCG ATC CAC CAG TAG AAC TTA T	1.902
NtNramp2-like	AWOK01S295926	XM_016621575	Nt5926_for	TTC CAC TAT GGC ATT ATC TTT CGC C	Nt5926_rev	TGC ACC AAA GCA GAG TAT AGG A	1.855
NtNramp3-like	AWOK01S026429	XM_009618066	Nt6429_for	AGT TCA TAT CAT CGG AGT CG	Nt6429_rev	TGA ACA AGT AGC CCA ATA GCC G	1.880
NtNramp6-like	AWOK01S066652	XM_016619903	Nt6652_for	GGG GTG GTT ACA GGA AGC AT	Nt6652_rev	ACC ACA CCA TAT TGG TAT TCT GA	1.964
NtZIP1-like	AWOK01S302253	XM_016652513	Nt2253_for	TGC TGC TGG TGT CAT TCT AG	Nt2253_rev	GCT GGC ATG ACT ATG ACT GTG	1.949
*NtZIP1-like**	AWOK01S276597	XM_016577112	Nt6597_for	GC ACT TCT AAG GAT GAT ACC T	Nt6597_rev	CTC CAC AAG CAA TTC CCA T	1.902
NtZIP4	AWOK01S102052	XM_016586154	Nt2052_for	TCT GTT TCC AAT ATT ACC TGC	Nt2052_rev	TTC TTG CCA ACT AAC GGG	1.882
NtZIP5-like	AWOK01S346858	XM_016648479	Nt6858_for	AGG TTA CAA TGA GCA AAG CTG	Nt6858_rev	AAC AAG TGA TAC ATT GGC AAG	1.702
NtZIP11-like	AWOK01S219909	XM_016644574	Nt9909_for	CTG ACA CAG ATT CCG ACT CA	Nt9909_rev	CAC AAT CAG CCA ACA TAG TAA GC	1.930
*NtZIP11-like**	AWOK01S001304	XM_016650095	Nt1304_for	CAA TGG GAT TGT CAC ACA AG	Nt1304_rev	GCT TGG CGA TAG CAT CC	1.834
NtMRP2-like	AWOK01S069080	XM_009799201	Nt6861_for	CAG AAG GTC CCG CCA TAA TTG	Nt6861_rev	CTG CCA ACT ACT CCG ACT TTG	1.790
NtMRP3-like	AWOK01S246455	XR_001973770	Nt6455_for	GCT GGC TTA GCT GTT ACA TAT GG	Nt6455_rev	GGA GGT TCA CTT GGA AGA GC	1.782
NtMRP5-like	AWOK01S453903	XM_016603310	Nt3903_for	GAG TTG CAG GAA GAT GCC AC	Nt3903_rev	CTA CCC TTA GTT GTA TGC CTG C	1.859
NtMRP10-like	AWOK01S246861	XM_016648918	Nt9080_for	AGG TGG AAC ATA ATT GGC TAT GG	Nt9080_rev	GGC CAA TGC AGA TAA GAT TCG	1.550
NtMRP14-like	AWOK01S250584	XM_016641186	Nt0584_for	GTG GAG CCA CTG CTT ATG TTG	Nt0584_rev	GCT GAG GTT GAT TCC TCG TTC	1.727
NtMRP15-like	AWOK01S055307	XM_016590897	Nt5307_for	CAT CGG CAT GTC CCA CAG	Nt5307_rev	CAA ATG ATG CCC GCA CTA GC	1.639
NtZIP1-like	*AWOK01S302253*	*XM_016652513*	***RACE PCR Reaction***				
			Nt2253race5	*GATTACGCCAAGCTT AGG CAT GAA CCC TTG GGG GAA ATT CC*	*Nt2253race3*	*GATTACGCCAAGCTT GAC CTT GGC TAT GTG AAA GTG CCT G*	
			AMPLIFICATION of full NtZIP1-like sequence for yeast experiment				
			Nt2253For35	*TCTAGA ATGAATAACCACAATGTC*	*Nt2253rev35*	*GGATCC AGC CCA TTT AGC CAT CAC*	
			Nt2253For36	*TCTAGA AAT AAC CAC AAT GTC CAA*	*Nt2253rev36*	*GGATCC TCA AGC CCA TTT AGC CAT CAC*	
			AMPLIFICATION of full NtZIP1-like sequence for pENTR/D-TOPO cloning				
			2253pEntSTART	CACC ATG AAT AAC CAC AAT GTC CAA GT	2253pEntSTOP	TCA AGC CCA TTT AGC CAT CAC AGA C	
