# Supplementary material for: Contribution of NtZIP1-Like to the Regulation of Zn Homeostasis
Source: Front Plant Sci. 2018 Feb 16;9:185. doi: 10.3389/fpls.2018.00185 (PMC5820362; doi:10.3389/fpls.2018.00185)

Supplementary Figure S1

RNA transcription levels of *PP2A* gene, presented as Cp mean values in diffrent samples: (A) in whole roots, whole leaves, stems, apical and basal segments of roots, young leaves and old leaves from plants grown at control conditions for 4 weeks, 6 weeks and 9 weeks; (B) under various Zn conditions; plants were grown in standard nutrient solution (control) and then transferred into modified control media: supplemented with 50 µM Zn for 1 day (1d); without Zn for 4 days (4d - Zn deficiency); plants grown at Zn-deficiency conditions for four days were transferred to the control medium for two days (6d - Zn replete).

(A)

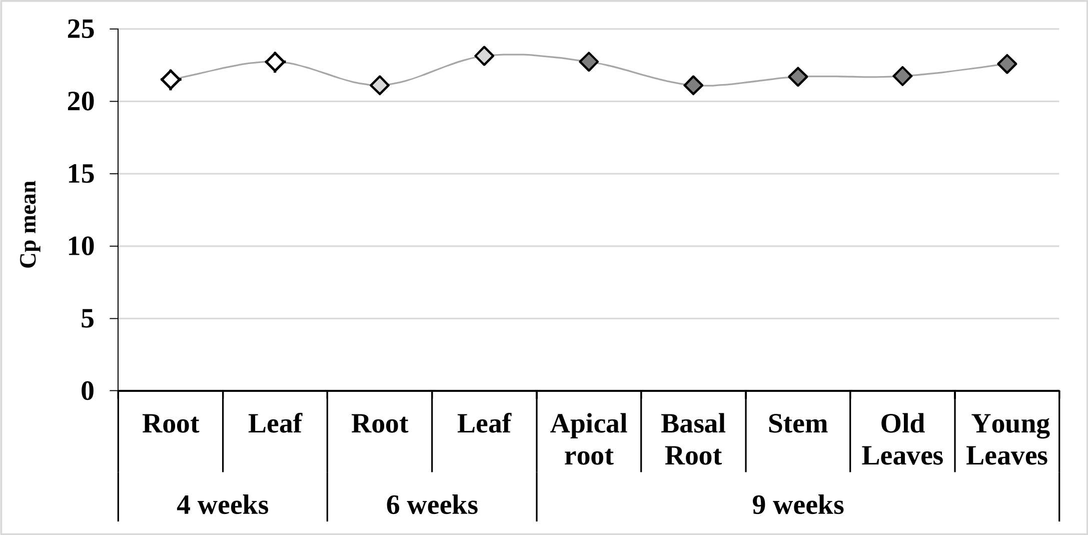

(B)

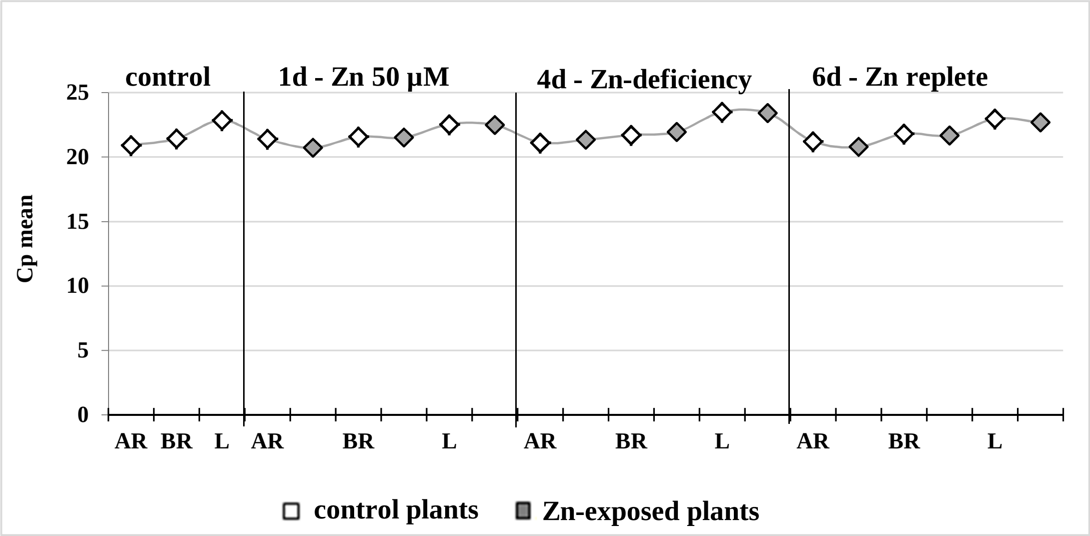

Supplement: Supplementary file 3 [file Image_1.pdf]
