# Supplementary material for: Contribution of NtZIP1-Like to the Regulation of Zn Homeostasis
Source: Front Plant Sci. 2018 Feb 16;9:185. doi: 10.3389/fpls.2018.00185 (PMC5820362; doi:10.3389/fpls.2018.00185)
Supplement: Supplementary file 4 [file Image_2.PDF]

Supplementary Figure S2 :

Alignments of nucleotide sequences of identified in tobacco metal transport genes from the following families: ZIP, NRAMP, MTP and MRP/ABCC, as best hits from *N. tabacum* with *A. thaliana* sequences used as a query.

The names of genes are annotated in the NCBI database. Asterisk indicates homologous sequences found in two different scaffolds. Primers used for the expression analysis are marked in grey (the list of primers is given in Supplementary Table S1; results of the expression analysis are shown in Figure 1).

>*NtZIP1-like* - XM_016652513

```
ATGAATAACCACAATGTCCAAGTTTGTTCTTACTGCTATAAAGCTGTAGTATTAACATGC
CTTGTCATTCTAGTCTTCGCCCCTGGAATCTCAGGTGAATGCACTTGCAACATCAAGGTT
GATCAACCGCGAAACACCAAAAACAGTAGTGATTCCCTTAGATACAGACTCATATCAATA
GTTTCAATACTGATTGCTGGTGCAATTGGGGTCAGTCTCCCACTTTTGGCAAGGAAAATT
GAAGCTCTGAGGCCCGAAAATGATATTTTCTTCATGATCAAGGCCTTTGCTGCTGGTGTC
ATTCTAGCCACTGGCTTCATCCACATATTGCCTGATGCATTTCAGACACTAACATCACCT
TGCCTGCAAGGCATGGACCCTTGGGGTAAATTCCCTTTCACAGGTTTCTTTGCCATGATC
GCCTCTATTGGATGTTTGATGATTGATACATTTGCAACGAGTTTTTACCAAAACAGGCAC
TTTCACATAGCCAAGCAGGTCAATATTGTAGACGAAGAAGCAGCCAGGGATGACATACAA
CACAGTCATAGTCATGCCAGCCATGTTGCACATGGGGCGACTCATTCTATCGGATCTGAT
CAAGAATTGATTCTGTCTGAAAATATACGAAATCGCATCATATCACAGGTGTTGGAGCTA
GGAATTCTGGTCCACTCCATAATAATTGGAGTTTCCTTAGGTGCCTCACAAAATACCGAA
ATGATAAAGCCTCTATTGGTTGCTCTGTCATTCCACCAATTCTTTGAGGGTATGGGACTT
GGAGGCTGCATTTCACAGGCAAAATTCAAGTCTACATCAACAGCAATCATGTCAGTTCTT
TTTTCTCTCACAACGCCAGCAGGAATTGGGATTGGGATCGGAATATCGAGGGTGTATAAT
GCCCATAGCTCTATTTCTCTAATAGTCGAAGGGATTCTGAATTCTGCTTCTTCAGGGATT
TTAATCTACATGGCCCTCGTTGATATACTAGCATCAGATTTTATGAATCCAAGGATGCAG
AACAATGTCAGACTTCTATGCGGGGCACACATTTCACTTCTTCTTGGAGCTGGGTGCATG
TCTGTGATGGCTAAATGGGCTTGA
```

PREDICTED: *Nicotiana tomentosiformis* **zinc transporter 1-like** (LOC104102230), mRNA
Sequence ID: XM_009609886.2Length: 1728Number of Matches: 1
Range 1: 472 to 1575GenBankGraphics Next Match Previous Match First Match

Alignment statistics for match #1

Score	Expect	Identities	Gaps	Strand	Frame
2039 bits(1104)	0.0()	1104/1104(100%)	0/1104(0%)	Plus/Plus	

```
Query  1    ATGAATAACCACAATGTCCAAGTTTGTTCTTACTGCTATAAAGCTGTAGTATTAACATGC  60
            ||||||||||||||||||||||||||||||||||||||||||||||||||||||||||||
Sbjct  472  ATGAATAACCACAATGTCCAAGTTTGTTCTTACTGCTATAAAGCTGTAGTATTAACATGC  531

Query  61   CTTGTCATTCTAGTCTTCGCCCCTGGAATCTCAGGTGAATGCACTTGCAACATCAAGGTT  120
            ||||||||||||||||||||||||||||||||||||||||||||||||||||||||||||
Sbjct  532  CTTGTCATTCTAGTCTTCGCCCCTGGAATCTCAGGTGAATGCACTTGCAACATCAAGGTT  591

Query  121  GATCAACCGCGAAACACCAAAAACAGTAGTGATTCCCTTAGATACAGACTCATATCAATA  180
            ||||||||||||||||||||||||||||||||||||||||||||||||||||||||||||
Sbjct  592  GATCAACCGCGAAACACCAAAAACAGTAGTGATTCCCTTAGATACAGACTCATATCAATA  651

Query  181  GTTTCAATACTGATTGCTGGTGCAATTGGGGTCAGTCTCCCACTTTTGGCAAGGAAAATT  240
            ||||||||||||||||||||||||||||||||||||||||||||||||||||||||||||
Sbjct  652  GTTTCAATACTGATTGCTGGTGCAATTGGGGTCAGTCTCCCACTTTTGGCAAGGAAAATT  711

Query  241  GAAGCTCTGAGGCCCGAAAATGATATTTTCTTCATGATCAAGGCCTTTGCTGCTGGTGTC  300
            ||||||||||||||||||||||||||||||||||||||||||||||||||||||||||||
Sbjct  712  GAAGCTCTGAGGCCCGAAAATGATATTTTCTTCATGATCAAGGCCTTTGCTGCTGGTGTC  771

Query  301  ATTCTAGCCACTGGCTTCATCCACATATTGCCTGATGCATTTCAGACACTAACATCACCT  360
            ||||||||||||||||||||||||||||||||||||||||||||||||||||||||||||
Sbjct  772  ATTCTAGCCACTGGCTTCATCCACATATTGCCTGATGCATTTCAGACACTAACATCACCT  831
```

```
Query  361    TGCCTGCAAGGCATGGACCCTTGGGGTAAATTCCCTTTCACAGGTTTCTTTGCCATGATC  420
              ||||||||||||||||||||||||||||||||||||||||||||||||||||||||||||
Sbjct  832    TGCCTGCAAGGCATGGACCCTTGGGGTAAATTCCCTTTCACAGGTTTCTTTGCCATGATC  891

Query  421    GCCTCTATTGGATGTTTGATGATTGATACATTTGCAACGAGTTTTTACCAAAACAGGCAC  480
              ||||||||||||||||||||||||||||||||||||||||||||||||||||||||||||
Sbjct  892    GCCTCTATTGGATGTTTGATGATTGATACATTTGCAACGAGTTTTTACCAAAACAGGCAC  951

Query  481    TTTCACATAGCCAAGCAGGTCAATATTGTAGACGAAGAAGCAGCCAGGGATGACATACAA  540
              ||||||||||||||||||||||||||||||||||||||||||||||||||||||||||||
Sbjct  952    TTTCACATAGCCAAGCAGGTCAATATTGTAGACGAAGAAGCAGCCAGGGATGACATACAA  1011

Query  541    CACAGTCATAGTCATGCCAGCCATGTTGCACATGGGGCGACTCATTCTATCGGATCTGAT  600
              ||||||||||||||||||||||||||||||||||||||||||||||||||||||||||||
Sbjct  1012   CACAGTCATAGTCATGCCAGCCATGTTGCACATGGGGCGACTCATTCTATCGGATCTGAT  1071

Query  601    CAAGAATTGATTCTGTCTGAAAATATACGAAATCGCATCATATCACAGGTGTTGGAGCTA  660
              ||||||||||||||||||||||||||||||||||||||||||||||||||||||||||||
Sbjct  1072   CAAGAATTGATTCTGTCTGAAAATATACGAAATCGCATCATATCACAGGTGTTGGAGCTA  1131

Query  661    GGAATTCTGGTCCACTCCATAATAATTGGAGTTTCCTTAGGTGCCTCACAAAATACCGAA  720
              ||||||||||||||||||||||||||||||||||||||||||||||||||||||||||||
Sbjct  1132   GGAATTCTGGTCCACTCCATAATAATTGGAGTTTCCTTAGGTGCCTCACAAAATACCGAA  1191

Query  721    ATGATAAAGCCTCTATTGGTTGCTCTGTCATTCCACCAATTCTTTGAGGGTATGGGACTT  780
              ||||||||||||||||||||||||||||||||||||||||||||||||||||||||||||
Sbjct  1192   ATGATAAAGCCTCTATTGGTTGCTCTGTCATTCCACCAATTCTTTGAGGGTATGGGACTT  1251

Query  781    GGAGGCTGCATTTCACAGGCAAAATTCAAGTCTACATCAACAGCAATCATGTCAGTTCTT  840
              ||||||||||||||||||||||||||||||||||||||||||||||||||||||||||||
Sbjct  1252   GGAGGCTGCATTTCACAGGCAAAATTCAAGTCTACATCAACAGCAATCATGTCAGTTCTT  1311

Query  841    TTTTCTCTCACAACGCCAGCAGGAATTGGGATTGGGATCGGAATATCGAGGGTGTATAAT  900
              ||||||||||||||||||||||||||||||||||||||||||||||||||||||||||||
Sbjct  1312   TTTTCTCTCACAACGCCAGCAGGAATTGGGATTGGGATCGGAATATCGAGGGTGTATAAT  1371

Query  901    GCCCATAGCTCTATTTCTCTAATAGTCGAAGGGATTCTGAATTCTGCTTCTTCAGGGATT  960
              ||||||||||||||||||||||||||||||||||||||||||||||||||||||||||||
Sbjct  1372   GCCCATAGCTCTATTTCTCTAATAGTCGAAGGGATTCTGAATTCTGCTTCTTCAGGGATT  1431

Query  961    TTAATCTACATGGCCCTCGTTGATATACTAGCATCAGATTTTATGAATCCAAGGATGCAG  1020
              ||||||||||||||||||||||||||||||||||||||||||||||||||||||||||||
Sbjct  1432   TTAATCTACATGGCCCTCGTTGATATACTAGCATCAGATTTTATGAATCCAAGGATGCAG  1491

Query  1021   AACAATGTCAGACTTCTATGCGGGGCACACATTTCACTTCTTCTTGGAGCTGGGTGCATG  1080
              ||||||||||||||||||||||||||||||||||||||||||||||||||||||||||||
Sbjct  1492   AACAATGTCAGACTTCTATGCGGGGCACACATTTCACTTCTTCTTGGAGCTGGGTGCATG  1551

Query  1081   TCTGTGATGGCTAAATGGGCTTGA  1104
              ||||||||||||||||||||||||
Sbjct  1552   TCTGTGATGGCTAAATGGGCTTGA  1575
```

> *NtZIP1-like - XM_016577112**

ATGTATGAGCTGGTGGGAAAACTAAATAATTTGAAGGGAAAATTAAAGCAGATAAATACA
GAAAAGTTCAGTCATATAAAAAGGCAGAAAGATAAAGCACTGGAAGAACTAATGCAATGT
CAAGCGGCTGAAAAAGGGGACCTAAACACCAAGTTCTTTCATAGTATGATGAAGGCCAGG
AGGAATATGAATATAGTATTTTCTATAACTGATTCACAAGGAGTAAATGTAACTGAGATG
GATGCAATTGCAAAATCTTTTATTGAGTTCTACACTACGTTTTTGGGTACTAACATGAGG
GAAAGGGAGTATGTAAATAGTAGCTTAGTTCGACAAGGACCAATAGTTACTGAAATACAA
AGGAGATTGTTGGAAGAAAAGTTTACAGAAAGAGAGGTTAAGATAGCTTTGTGGAAAATT
AATGGAGAAAAATTACCAGGTCTTGATGGTTATGAAAGGAAGTTCTTCAAAGTTAGTTGG
GAAGTGGTGGAAAAAGATGTAGTGGATGCTGTAATGGAATTGTTCAGAATAGGAAAAATG
CTAAGGGTGTTGAATCACACAGTGATTACCATTATACCTAAGAGTTCTCATGCTACTAGT
GACTTGGTAAGGCTGTACAACAGGAAGAATACTACACAGAGCTGTTTGATCAAGATTGAC
GTCAAAAAGGCATATAATACTGTAGAGTGGTCGTTTGTGGAAGAAATTCTCCATGCAATG
AATTTCCCTCAGAGATTCATTAAGTGGATAATGGCATGTATAACTACTACTCCATATAGC
ATAGCAATAAATATGGGGCTATATGGGAGTATTACAGGGAAGAGAGGTCTCCGACAGATG
ATATACTGTTATTTTTGCAAAGGCAACTATCAGTCAATAATGCTTTTACTAAGAGGACTG
CAGTCTTTCTCTAACGCTTCAGGCTTGAGCACTAATGCAGAAAAGTCAAATATTTTCAGT
GCTAATATGGAATCCCAACTATTAGAAGATTTATGTGAGAAAACTGGTTATCAGAGAGGG

GCATTACCATTTCGATACCTGGGGGTGCCTATCTCAGCAAAGAAGATCTCCACCATGGAC
TGCGAAGTACTAGTAGATAAGATTACTGCTAGACTAAAGATCTGGAGCTCGAAGAACATT
TCATATGCTGGTAGAGTACAATTAATTAACCCAGTTCTTTTACAAAATGAGGCAACAATA
GCAAAATATGTGTGGAACATTGCTCAAAAAGCAGACAATTTGTGGGTGAAGTGCGTTGAT
CACATTTACATAAAAGGTACCAGTTGGAAGCAATACTTGCCACCGACTGATTGCAGCTGG
TACTGGAAGAGAATATGCAATGTAATAGAAAAGTTCAAGGGGGGATATGTGGGGAATGAA
TGGCAACAAATAGGAGGAAGTTTTATAAGCTGGCTGTCAGTACATAAAAGATTGCTGACA
AAGGAAAAGCTGCAAAAAATTGGAGTATGCCAAGAGACAAGATGTGAAATGTGTGGAGCA
ATCATAGAAACAATCCAACATCTGTTTTTTGAATGTCATTTCTCGAAGGAATTCTTGGGG
CTTCTGATTGAGTGGCTAGAAATGAGGATAAATACACCAGATATTGAAGGGTTGTGGAAA
AAATTGACAAGGAATATCAAAGCTACAAAGGGAGAAGCATGGAGAAATCTATGGACAATT
TCATTGCACAAGATATTTGCAGCCGTTGCAATGGGAATTGCACTTCTAAGGATGATACCT
AAGAGACCATTTCTACTTACTTGTGCTTACTCTTTTGCCTTTGCTATTTCAAGCCCTATA
GGTGTTGGAATAGGAATTGCAATTGATGCTACAAGTGAAGGAAAAACAGCAGATTGGACT
TATGCCATTTCCATGGGAATTGCTTGTGGAGTTTTCATATATGTGGCAATAAATCACCTT
ATATCAAAAGGTTTTAGGCCACAGAACAAATGTTATTTTGACACTCAATTCTTCAAGTTT
TTTGCTGTGTTTTTAGGAGTTGGGACTATTGCTATAGTCATGATATGGGACTAA

PREDICTED: *Nicotiana tabacum* **zinc transporter 1-like** (LOC107759225), mRNA
Sequence ID: XM_016577112.1Length: 1357Number of Matches: 1
Range 1: 786 to 1179GenBankGraphics Next Match Previous Match First Match

Alignment statistics for match #1

Score	Expect	Identities	Gaps	Strand	Frame
711 bits(788)	0.0()	394/394(100%)	0/394(0%)	Plus/Plus	

```
Query  1641   AGCTACAAAGGGAGAAGCATGGAGAAATCTATGGACAATTTCATTGCACAAGATATTTGC  1700
              ||||||||||||||||||||||||||||||||||||||||||||||||||||||||||||
Sbjct  786    AGCTACAAAGGGAGAAGCATGGAGAAATCTATGGACAATTTCATTGCACAAGATATTTGC  845

Query  1701   AGCCGTTGCAATGGGAATTGCACTTCTAAGGATGATACCTAAGAGACCATTTCTACTTAC  1760
              ||||||||||||||||||||||||||||||||||||||||||||||||||||||||||||
Sbjct  846    AGCCGTTGCAATGGGAATTGCACTTCTAAGGATGATACCTAAGAGACCATTTCTACTTAC  905

Query  1761   TTGTGCTTACTCTTTTGCCTTTGCTATTTCAAGCCCTATAGGTGTTGGAATAGGAATTGC  1820
              ||||||||||||||||||||||||||||||||||||||||||||||||||||||||||||
Sbjct  906    TTGTGCTTACTCTTTTGCCTTTGCTATTTCAAGCCCTATAGGTGTTGGAATAGGAATTGC  965

Query  1821   AATTGATGCTACAAGTGAAGGAAAAACAGCAGATTGGACTTATGCCATTTCCATGGGAAT  1880
              ||||||||||||||||||||||||||||||||||||||||||||||||||||||||||||
Sbjct  966    AATTGATGCTACAAGTGAAGGAAAAACAGCAGATTGGACTTATGCCATTTCCATGGGAAT  1025

Query  1881   TGCTTGTGGAGTTTTCATATATGTGGCAATAAATCACCTTATATCAAAAGGTTTTAGGCC  1940
              ||||||||||||||||||||||||||||||||||||||||||||||||||||||||||||
Sbjct  1026   TGCTTGTGGAGTTTTCATATATGTGGCAATAAATCACCTTATATCAAAAGGTTTTAGGCC  1085

Query  1941   ACAGAACAAATGTTATTTTGACACTCAATTCTTCAAGTTTTTTGCTGTGTTTTTAGGAGT  2000
              ||||||||||||||||||||||||||||||||||||||||||||||||||||||||||||
Sbjct  1086   ACAGAACAAATGTTATTTTGACACTCAATTCTTCAAGTTTTTTGCTGTGTTTTTAGGAGT  1145

Query  2001   TGGGACTATTGCTATAGTCATGATATGGGACTAA  2034
              ||||||||||||||||||||||||||||||||||
Sbjct  1146   TGGGACTATTGCTATAGTCATGATATGGGACTAA  1179
```

>*NtZIP4* - XM_016586154
ATGTCGTTCACTGAGGATCTCGTGCCCTTCTTTTTTATGGACCCAAAAATTAGAGAAAAGA
CCGGGGCTTTCTCAGATACCGTTATGCTGAAACTTTATCAATCTGTTTCCAATATTACCTG
CGGCAGTGCTGATGAAGAGATAGAAGGCTGCCGAGACAGCTCGGCTGCTCTTACCCTTAAA
ATCGTGGCTATCTCTGCCATCCTAATAGCTAGTACTTGCGGAGTTGGTATCCCGTTAGTTG
GCAAGAAGCATCGGTTCCTCCGAACTGACTCTAATCTCTTTCTTGCTGTTAAAGCCTTTGC
TGCTGGTGTCATCCTCTCTACAGGCTTTGTCCACATATTACCAGGCGCCACCTCATCATTA
ACTAATCCTTGTCTTCCGAAATCTCCTTGGTTGAAATTCCCTTTCGCTGGTTTTATCGCCA
TGATGGCTGCATTGACTACCTTGGTGGTTGACTTTGTTGGGACTCAGTATTATGAGAGGAA

GCAAGAGAAACAAAGCCAAAAAGATCAGATTGATTCAGTGGATTTGGTGTCAGAATCAGCT
ATTGTACCAGTTGAACCAAAGGCAGGGAATGAGAAATTGTTTGGTGAAGAAGATGGTGGTG
CAATACACATTGTTGGGATGCATGCACATGCAGCTCATCACAGACATAGCCATTCACAAGA
ACAAGGGGCATGTCAAGGGAACGTGAGGGAGCATTCCCATGGTCATTCGCACTCCCATAGC
TTTGGTGGTGGAGATGAGGAAGGTGGAGGGAGGCATGTTGTTGTTTCTCAGGTCTTGGAGC
TGGGAATAGTATCACATTCTCTCATAATAGGCATAGCATTGGGTGTTTCAGAAAGTCCATG
CACAATTAGACCCTTGCTCGTGGCCTTATCGTTCCACCAGTTCTTCGAAGGTTTTGCGTTA
GGAGGTTGCATCTCGCAGGCACAGTTCAATTCCCTCCGTTCCACTATAATGGCAACGTTTT
TCGCCGTAACAACACCCTTGGGAATTGCTATAGGAATTCTAGCTTCTTCATCTTACAATCC
ACATAGCCCAAGAGCTTTGGTAGTGGAAGGGAGCCTTAACTCTATATCTGCTGGAATTTTA
ATCTACATGGCTTTAGTAGACCTAATTGCTGCAGATTTCTTGAGTAAAAGAATGAGCTGCA
ATACAAGGCTTCAAATAGTATCTTATTTTGCACTATTCTTAGGGGCTAGACTCATGTCCCT
TCTTGCAATATGGGCATGA

PREDICTED: Nicotiana tabacum **zinc transporter 4**, (LOC107767236), mRNA
Sequence ID: XM_016586154.1Length: 1816Number of Matches: 1
Range 1: 360 to 1598GenBankGraphics Next Match Previous Match First Match

Alignment statistics for match #1

Score	Expect	Identities	Gaps	Strand	Frame
2235 bits(2478)	0.0()	1239/1239(100%)	0/1239(0%)	Plus/Plus	

```
Query  1     ATGTCGTTCACTGAGGATCTCGTGCCCTTCTTTTTTATGGACCCAAAAATTAGAGAAAAG  60
             ||||||||||||||||||||||||||||||||||||||||||||||||||||||||||||
Sbjct  360   ATGTCGTTCACTGAGGATCTCGTGCCCTTCTTTTTTATGGACCCAAAAATTAGAGAAAAG  419

Query  61    ACCGGGGCTTTCTCAGATACCGTTATGCTGAAACTTTATCAATCTGTTTCCAATATTACC  120
             ||||||||||||||||||||||||||||||||||||||||||||||||||||||||||||
Sbjct  420   ACCGGGGCTTTCTCAGATACCGTTATGCTGAAACTTTATCAATCTGTTTCCAATATTACC  479

Query  121   TGCGGCAGTGCTGATGAAGAGATAGAAGGCTGCCGAGACAGCTCGGCTGCTCTTACCCTT  180
             ||||||||||||||||||||||||||||||||||||||||||||||||||||||||||||
Sbjct  480   TGCGGCAGTGCTGATGAAGAGATAGAAGGCTGCCGAGACAGCTCGGCTGCTCTTACCCTT  539

Query  181   AAAATCGTGGCTATCTCTGCCATCCTAATAGCTAGTACTTGCGGAGTTGGTATCCCGTTA  240
             ||||||||||||||||||||||||||||||||||||||||||||||||||||||||||||
Sbjct  540   AAAATCGTGGCTATCTCTGCCATCCTAATAGCTAGTACTTGCGGAGTTGGTATCCCGTTA  599

Query  241   GTTGGCAAGAAGCATCGGTTCCTCCGAACTGACTCTAATCTCTTTCTTGCTGTTAAAGCC  300
             ||||||||||||||||||||||||||||||||||||||||||||||||||||||||||||
Sbjct  600   GTTGGCAAGAAGCATCGGTTCCTCCGAACTGACTCTAATCTCTTTCTTGCTGTTAAAGCC  659

Query  301   TTTGCTGCTGGTGTCATCCTCTCTACAGGCTTTGTCCACATATTACCAGGCGCCACCTCA  360
             ||||||||||||||||||||||||||||||||||||||||||||||||||||||||||||
Sbjct  660   TTTGCTGCTGGTGTCATCCTCTCTACAGGCTTTGTCCACATATTACCAGGCGCCACCTCA  719

Query  361   TCATTAACTAATCCTTGTCTTCCGAAATCTCCTTGGTTGAAATTCCCTTTCGCTGGTTTT  420
             ||||||||||||||||||||||||||||||||||||||||||||||||||||||||||||
Sbjct  720   TCATTAACTAATCCTTGTCTTCCGAAATCTCCTTGGTTGAAATTCCCTTTCGCTGGTTTT  779

Query  421   ATCGCCATGATGGCTGCATTGACTACCTTGGTGGTTGACTTTGTTGGGACTCAGTATTAT  480
             ||||||||||||||||||||||||||||||||||||||||||||||||||||||||||||
Sbjct  780   ATCGCCATGATGGCTGCATTGACTACCTTGGTGGTTGACTTTGTTGGGACTCAGTATTAT  839

Query  481   GAGAGGAAGCAAGAGAAACAAAGCCAAAAAGATCAGATTGATTCAGTGGATTTGGTGTCA  540
             ||||||||||||||||||||||||||||||||||||||||||||||||||||||||||||
Sbjct  840   GAGAGGAAGCAAGAGAAACAAAGCCAAAAAGATCAGATTGATTCAGTGGATTTGGTGTCA  899

Query  541   GAATCAGCTATTGTACCAGTTGAACCAAAGGCAGGGAATGAGAAATTGTTTGGTGAAGAA  600
             ||||||||||||||||||||||||||||||||||||||||||||||||||||||||||||
Sbjct  900   GAATCAGCTATTGTACCAGTTGAACCAAAGGCAGGGAATGAGAAATTGTTTGGTGAAGAA  959

Query  601   GATGGTGGTGCAATACACATTGTTGGGATGCATGCACATGCAGCTCATCACAGACATAGC  660
             ||||||||||||||||||||||||||||||||||||||||||||||||||||||||||||
Sbjct  960   GATGGTGGTGCAATACACATTGTTGGGATGCATGCACATGCAGCTCATCACAGACATAGC  1019

Query  661   CATTCACAAGAACAAGGGGCATGTCAAGGGAACGTGAGGGAGCATTCCCATGGTCATTCG  720
             ||||||||||||||||||||||||||||||||||||||||||||||||||||||||||||
Sbjct  1020  CATTCACAAGAACAAGGGGCATGTCAAGGGAACGTGAGGGAGCATTCCCATGGTCATTCG  1079
```

```
Query  721   CACTCCCATAGCTTTGGTGGTGGAGATGAGGAAGGTGGAGGGAGGCATGTTGTTGTTTCT  780
             ||||||||||||||||||||||||||||||||||||||||||||||||||||||||||||
Sbjct  1080  CACTCCCATAGCTTTGGTGGTGGAGATGAGGAAGGTGGAGGGAGGCATGTTGTTGTTTCT  1139

Query  781   CAGGTCTTGGAGCTGGGAATAGTATCACATTCTCTCATAATAGGCATAGCATTGGGTGTT  840
             ||||||||||||||||||||||||||||||||||||||||||||||||||||||||||||
Sbjct  1140  CAGGTCTTGGAGCTGGGAATAGTATCACATTCTCTCATAATAGGCATAGCATTGGGTGTT  1199

Query  841   TCAGAAAGTCCATGCACAATTAGACCCTTGCTCGTGGCCTTATCGTTCCACCAGTTCTTC  900
             ||||||||||||||||||||||||||||||||||||||||||||||||||||||||||||
Sbjct  1200  TCAGAAAGTCCATGCACAATTAGACCCTTGCTCGTGGCCTTATCGTTCCACCAGTTCTTC  1259

Query  901   GAAGGTTTTGCGTTAGGAGGTTGCATCTCGCAGGCACAGTTCAATTCCCTCCGTTCCACT  960
             ||||||||||||||||||||||||||||||||||||||||||||||||||||||||||||
Sbjct  1260  GAAGGTTTTGCGTTAGGAGGTTGCATCTCGCAGGCACAGTTCAATTCCCTCCGTTCCACT  1319

Query  961   ATAATGGCAACGTTTTTCGCCGTAACAACACCCTTGGGAATTGCTATAGGAATTCTAGCT  1020
             ||||||||||||||||||||||||||||||||||||||||||||||||||||||||||||
Sbjct  1320  ATAATGGCAACGTTTTTCGCCGTAACAACACCCTTGGGAATTGCTATAGGAATTCTAGCT  1379

Query  1021  TCTTCATCTTACAATCCACATAGCCCAAGAGCTTTGGTAGTGGAAGGGAGCCTTAACTCT  1080
             ||||||||||||||||||||||||||||||||||||||||||||||||||||||||||||
Sbjct  1380  TCTTCATCTTACAATCCACATAGCCCAAGAGCTTTGGTAGTGGAAGGGAGCCTTAACTCT  1439

Query  1081  ATATCTGCTGGAATTTTAATCTACATGGCTTTAGTAGACCTAATTGCTGCAGATTTCTTG  1140
             ||||||||||||||||||||||||||||||||||||||||||||||||||||||||||||
Sbjct  1440  ATATCTGCTGGAATTTTAATCTACATGGCTTTAGTAGACCTAATTGCTGCAGATTTCTTG  1499

Query  1141  AGTAAAAGAATGAGCTGCAATACAAGGCTTCAAATAGTATCTTATTTTGCACTATTCTTA  1200
             ||||||||||||||||||||||||||||||||||||||||||||||||||||||||||||
Sbjct  1500  AGTAAAAGAATGAGCTGCAATACAAGGCTTCAAATAGTATCTTATTTTGCACTATTCTTA  1559

Query  1201  GGGGCTAGACTCATGTCCCTTCTTGCAATATGGGCATGA  1239
             |||||||||||||||||||||||||||||||||||||||
Sbjct  1560  GGGGCTAGACTCATGTCCCTTCTTGCAATATGGGCATGA  1598
```

>*NtZIP5-like* - XM_016648479

```
ATGATTACTCAACCTTTACTTCTCAAAATCACAACCCTAATCTACTTTTCAA
TCCTTTTCCAATCAACCCCAATCTTTGCAAATTGTACATGTGAACCCCAAGACAAAGATCA
TCAAAAAACCAAACATGAAGCCTTAGCCTACAAACTAGTAGCCATAGCTTCAATACTTTGT
GCAAGTGCACTTGGTATTGTCATCCCAATTCTCTTGAACAACTTCAAATCATTGCAAAATG
ATTATTCATCTCTTAATTTCTTGATCAAAGCTTTCGCGGCGGGGGTAATTCTTGCCACTGG
ATTCATTCACATTTTACCTGATGCATTTCAGAGCTTGACAAACCCTTGCCTTAATAGTGAA
ATATTTGGGAGTTTTCCATTTGCTGGTTTTGTTTCAATGATGTTTGCTATTTTTACTTTGA
TGATGGAGTCATTTGCAACTGGATATCATAGAAGAGCTGAGCTTAGAAAAGCTCAGCCAGT
GAATATTGGAGATGGTGAAGAAAATGATGGTATCCATACACATGGATATGGACAAGGACAT
GAGCATATTTCTCATGGTCCTCATATTTTGTTAGAAAGAACCAATTCTTCTAGTCTTATAC
GGCACAGGTTAATATCACAGATTTTGGAGCTGGGAATTTTGGTTCATTCTGTGATTATTGG
TATTTCGCTTGGAACTACAGAAAATCCCAAAACAATCAAACCTCTAATAATAGCTTTGAGT
TTTCATCAATTTTTTGAAGGCATGGGACTTGGTGGGTGCATTTCCCAGGCGAAGTACAGGG
CAAGAACAATAGCAATAATGGTATTGTTCTTCACTCTTACAACTCCAAGTGGAATAGCTAT
TGGAATGATGATATCAAAAGGTTACAATGAGCAAAGCTGGGCAGCATTGATTGTGCAAGGG
GTTCTTAATTCAGCATCAGCTGGTATTCTAATTTACATGGCGCTTGTGGATCTTCTTGCAA
AAGATTTCATGGACCCTAATTTACACAGTAGTTTCAAGCTCCAAATTCTTGCCAATGTATC
ACTTGTTTTGGGAGCTTCTTGTATGTCACTATTGGCAAAATGGGGTGGACATAA
```

PREDICTED: Nicotiana tomentosiformis **zinc transporter 5-like** (LOC104093480), transcript variant X1, mRNA
Sequence ID: XM_009599231.2Length: 1282Number of Matches: 1
Range 1: 118 to 1200GenBankGraphics Next Match Previous Match First Match

Alignment statistics for match #1

Score	Expect	Identities	Gaps	Strand	Frame
1954 bits(2166)	0.0()	1083/1083(100%)	0/1083(0%)	Plus/Plus	

```
Query  1     ATGATTACTCAACCTTTACTTCTCAAAATCACAACCCTAATCTACTTTTCAATCCTTTTC  60
             ||||||||||||||||||||||||||||||||||||||||||||||||||||||||||||
```

```
Sbjct  118   ATGATTACTCAACCTTTACTTCTCAAAATCACAACCCTAATCTACTTTTCAATCCTTTTC  177

Query  61    CAATCAACCCCAATCTTTGCAAATTGTACATGTGAACCCCAAGACAAAGATCATCAAAAA  120
             ||||||||||||||||||||||||||||||||||||||||||||||||||||||||||||
Sbjct  178   CAATCAACCCCAATCTTTGCAAATTGTACATGTGAACCCCAAGACAAAGATCATCAAAAA  237

Query  121   ACCAAACATGAAGCCTTAGCCTACAAACTAGTAGCCATAGCTTCAATACTTTGTGCAAGT  180
             ||||||||||||||||||||||||||||||||||||||||||||||||||||||||||||
Sbjct  238   ACCAAACATGAAGCCTTAGCCTACAAACTAGTAGCCATAGCTTCAATACTTTGTGCAAGT  297

Query  181   GCACTTGGTATTGTCATCCCAATTCTCTTGAACAACTTCAAATCATTGCAAAATGATTAT  240
             ||||||||||||||||||||||||||||||||||||||||||||||||||||||||||||
Sbjct  298   GCACTTGGTATTGTCATCCCAATTCTCTTGAACAACTTCAAATCATTGCAAAATGATTAT  357

Query  241   TCATCTCTTAATTTCTTGATCAAAGCTTTCGCGGCGGGGGTAATTCTTGCCACTGGATTC  300
             ||||||||||||||||||||||||||||||||||||||||||||||||||||||||||||
Sbjct  358   TCATCTCTTAATTTCTTGATCAAAGCTTTCGCGGCGGGGGTAATTCTTGCCACTGGATTC  417

Query  301   ATTCACATTTTACCTGATGCATTTCAGAGCTTGACAAACCCTTGCCTTAATAGTGAAATA  360
             ||||||||||||||||||||||||||||||||||||||||||||||||||||||||||||
Sbjct  418   ATTCACATTTTACCTGATGCATTTCAGAGCTTGACAAACCCTTGCCTTAATAGTGAAATA  477

Query  361   TTTGGGAGTTTTCCATTTGCTGGTTTTGTTTCAATGATGTTTGCTATTTTTACTTTGATG  420
             ||||||||||||||||||||||||||||||||||||||||||||||||||||||||||||
Sbjct  478   TTTGGGAGTTTTCCATTTGCTGGTTTTGTTTCAATGATGTTTGCTATTTTTACTTTGATG  537

Query  421   ATGGAGTCATTTGCAACTGGATATCATAGAAGAGCTGAGCTTAGAAAAGCTCAGCCAGTG  480
             ||||||||||||||||||||||||||||||||||||||||||||||||||||||||||||
Sbjct  538   ATGGAGTCATTTGCAACTGGATATCATAGAAGAGCTGAGCTTAGAAAAGCTCAGCCAGTG  597

Query  481   AATATTGGAGATGGTGAAGAAAATGATGGTATCCATACACATGGATATGGACAAGGACAT  540
             ||||||||||||||||||||||||||||||||||||||||||||||||||||||||||||
Sbjct  598   AATATTGGAGATGGTGAAGAAAATGATGGTATCCATACACATGGATATGGACAAGGACAT  657

Query  541   GAGCATATTTCTCATGGTCCTCATATTTTGTTAGAAAGAACCAATTCTTCTAGTCTTATA  600
             ||||||||||||||||||||||||||||||||||||||||||||||||||||||||||||
Sbjct  658   GAGCATATTTCTCATGGTCCTCATATTTTGTTAGAAAGAACCAATTCTTCTAGTCTTATA  717

Query  601   CGGCACAGGTTAATATCACAGATTTTGGAGCTGGGAATTTTGGTTCATTCTGTGATTATT  660
             ||||||||||||||||||||||||||||||||||||||||||||||||||||||||||||
Sbjct  718   CGGCACAGGTTAATATCACAGATTTTGGAGCTGGGAATTTTGGTTCATTCTGTGATTATT  777

Query  661   GGTATTTCGCTTGGAACTACAGAAAATCCCAAAACAATCAAACCTCTAATAATAGCTTTG  720
             ||||||||||||||||||||||||||||||||||||||||||||||||||||||||||||
Sbjct  778   GGTATTTCGCTTGGAACTACAGAAAATCCCAAAACAATCAAACCTCTAATAATAGCTTTG  837

Query  721   AGTTTTCATCAATTTTTTGAAGGCATGGGACTTGGTGGGTGCATTTCCCAGGCGAAGTAC  780
             ||||||||||||||||||||||||||||||||||||||||||||||||||||||||||||
Sbjct  838   AGTTTTCATCAATTTTTTGAAGGCATGGGACTTGGTGGGTGCATTTCCCAGGCGAAGTAC  897

Query  781   AGGGCAAGAACAATAGCAATAATGGTATTGTTCTTCACTCTTACAACTCCAAGTGGAATA  840
             ||||||||||||||||||||||||||||||||||||||||||||||||||||||||||||
Sbjct  898   AGGGCAAGAACAATAGCAATAATGGTATTGTTCTTCACTCTTACAACTCCAAGTGGAATA  957

Query  841   GCTATTGGAATGATGATATCAAAAGGTTACAATGAGCAAAGCTGGGCAGCATTGATTGTG  900
             ||||||||||||||||||||||||||||||||||||||||||||||||||||||||||||
Sbjct  958   GCTATTGGAATGATGATATCAAAAGGTTACAATGAGCAAAGCTGGGCAGCATTGATTGTG  1017

Query  901   CAAGGGGTTCTTAATTCAGCATCAGCTGGTATTCTAATTTACATGGCGCTTGTGGATCTT  960
             ||||||||||||||||||||||||||||||||||||||||||||||||||||||||||||
Sbjct  1018  CAAGGGGTTCTTAATTCAGCATCAGCTGGTATTCTAATTTACATGGCGCTTGTGGATCTT  1077

Query  961   CTTGCAAAAGATTTCATGGACCCTAATTTACACAGTAGTTTCAAGCTCCAAATTCTTGCC  1020
             ||||||||||||||||||||||||||||||||||||||||||||||||||||||||||||
Sbjct  1078  CTTGCAAAAGATTTCATGGACCCTAATTTACACAGTAGTTTCAAGCTCCAAATTCTTGCC  1137

Query  1021  AATGTATCACTTGTTTTGGGAGCTTCTTGTATGTCACTATTGGCAAAATGGGGTGGGACA  1080
             ||||||||||||||||||||||||||||||||||||||||||||||||||||||||||||
Sbjct  1138  AATGTATCACTTGTTTTGGGAGCTTCTTGTATGTCACTATTGGCAAAATGGGGTGGGACA  1197

Query  1081  TAA  1083
             |||
Sbjct  1198  TAA  1200
```

>*NtZIP11-like* - XM_016644574

```
ATGGCACGTTATCAACCCCTTTTCTTCCTCAGTCTCTTTCTTCTTCTCATCATCTCCGCC
GCTGCCCACGGCGGTGCTCACGACGGAGATGCTGACACAGATTCCGACTCACCTGCACCC
TCCGATAAACCCCATCTGAGATCTCGACCGTTGGTTCTTGTAAAAATATGGTGTTTGATT
ATAGTCTTTGTAGGGACTTTTCTTGGTGGAATATCACCTTATTTTATGAAATGGAATGAA
GGGTTTTTGGTTCTTGGGACACAATTTGCTGGTGGTGTGTTTTTAGGGACAGCACTTATG
CATTTCTTGAGTGATTCAAATGAGACATTTGGAGATTTGACGAATAAAGAATACCCTTTT
GCTTTTATGTTGGCATGTGCTGGTTATTTGCTTACTATGTTGGCTGATTGTGTTATCTGC
TTTGTTTATGCCAAGCAGAATAACAGCAACGATGTTCAGCTCCAAGGTGCTACTGAAAAT
GGAAAAGGCAATGGGGTAGGGCAGTCACAGGTCTGTGATGGTAGAGAAGATTACTTTTCA
AAAGCACCTCTTGCAACTGCATCTTCACTTGGTGATAGCATCCTATTAATCGTGGCGCTG
TGTTTCCATTCTGTCTTTGAGGGAATTGCCATTGGCGTCGCAGATTCAAAAGCCGATGCT
TGGAGAGCTCTTTGGACTGTCTGTCTACACAAGATATTTGCTGCTATTGCAATGGGAATA
GCTCTACTTAGGATGATCCCAAATCGTCCGTTGTTATCTTGTGCAGCATATGCTTTTGCC
TTTGCCATCTCTAGTCCAATTGGTGTTGCCATTGGAATCATAATTGATGCTACAACTCAA
GGGGTTGTTGCAGACTGGATCTTTGCTATATCAATGGGATTGGCTTGTGGGGTTTTTGTC
TATGTATCCATTAATCATTTGCTTTCAAGAGGATACAAGCCTCAGAAAATGGTCTTAGTT
GACAAGCCTCATTTCAAGTTTTTGGCTGTATTGTTAGGTGTTGGAGCGATTGCTGTGGTC
ATGATATGGGACACTTGA
```

PREDICTED: Nicotiana tabacum **zinc transporter 11-like** (LOC107818543), mRNA
Sequence ID: XM_016644574.1Length: 1533Number of Matches: 1
Range 1: 112 to 1149GenBankGraphics Next Match Previous Match First Match

Alignment statistics for match #1

Score	Expect	Identities	Gaps	Strand	Frame
1873 bits(2076)	0.0()	1038/1038(100%)	0/1038(0%)	Plus/Plus	

```
Query  1    ATGGCACGTTATCAACCCCTTTTCTTCCTCAGTCTCTTTCTTCTTCTCATCATCTCCGCC  60
            ||||||||||||||||||||||||||||||||||||||||||||||||||||||||||||
Sbjct  112  ATGGCACGTTATCAACCCCTTTTCTTCCTCAGTCTCTTTCTTCTTCTCATCATCTCCGCC  171

Query  61   GCTGCCCACGGCGGTGCTCACGACGGAGATGCTGACACAGATTCCGACTCACCTGCACCC  120
            ||||||||||||||||||||||||||||||||||||||||||||||||||||||||||||
Sbjct  172  GCTGCCCACGGCGGTGCTCACGACGGAGATGCTGACACAGATTCCGACTCACCTGCACCC  231

Query  121  TCCGATAAACCCCATCTGAGATCTCGACCGTTGGTTCTTGTAAAAATATGGTGTTTGATT  180
            ||||||||||||||||||||||||||||||||||||||||||||||||||||||||||||
Sbjct  232  TCCGATAAACCCCATCTGAGATCTCGACCGTTGGTTCTTGTAAAAATATGGTGTTTGATT  291

Query  181  ATAGTCTTTGTAGGGACTTTTCTTGGTGGAATATCACCTTATTTTATGAAATGGAATGAA  240
            ||||||||||||||||||||||||||||||||||||||||||||||||||||||||||||
Sbjct  292  ATAGTCTTTGTAGGGACTTTTCTTGGTGGAATATCACCTTATTTTATGAAATGGAATGAA  351

Query  241  GGGTTTTTGGTTCTTGGGACACAATTTGCTGGTGGTGTGTTTTTAGGGACAGCACTTATG  300
            ||||||||||||||||||||||||||||||||||||||||||||||||||||||||||||
Sbjct  352  GGGTTTTTGGTTCTTGGGACACAATTTGCTGGTGGTGTGTTTTTAGGGACAGCACTTATG  411

Query  301  CATTTCTTGAGTGATTCAAATGAGACATTTGGAGATTTGACGAATAAAGAATACCCTTTT  360
            ||||||||||||||||||||||||||||||||||||||||||||||||||||||||||||
Sbjct  412  CATTTCTTGAGTGATTCAAATGAGACATTTGGAGATTTGACGAATAAAGAATACCCTTTT  471

Query  361  GCTTTTATGTTGGCATGTGCTGGTTATTTGCTTACTATGTTGGCTGATTGTGTTATCTGC  420
            ||||||||||||||||||||||||||||||||||||||||||||||||||||||||||||
Sbjct  472  GCTTTTATGTTGGCATGTGCTGGTTATTTGCTTACTATGTTGGCTGATTGTGTTATCTGC  531

Query  421  TTTGTTTATGCCAAGCAGAATAACAGCAACGATGTTCAGCTCCAAGGTGCTACTGAAAAT  480
            ||||||||||||||||||||||||||||||||||||||||||||||||||||||||||||
Sbjct  532  TTTGTTTATGCCAAGCAGAATAACAGCAACGATGTTCAGCTCCAAGGTGCTACTGAAAAT  591

Query  481  GGAAAAGGCAATGGGGTAGGGCAGTCACAGGTCTGTGATGGTAGAGAAGATTACTTTTCA  540
            ||||||||||||||||||||||||||||||||||||||||||||||||||||||||||||
Sbjct  592  GGAAAAGGCAATGGGGTAGGGCAGTCACAGGTCTGTGATGGTAGAGAAGATTACTTTTCA  651

Query  541  AAAGCACCTCTTGCAACTGCATCTTCACTTGGTGATAGCATCCTATTAATCGTGGCGCTG  600
            ||||||||||||||||||||||||||||||||||||||||||||||||||||||||||||
Sbjct  652  AAAGCACCTCTTGCAACTGCATCTTCACTTGGTGATAGCATCCTATTAATCGTGGCGCTG  711

Query  601  TGTTTCCATTCTGTCTTTGAGGGAATTGCCATTGGCGTCGCAGATTCAAAAGCCGATGCT  660
```

```
                ||||||||||||||||||||||||||||||||||||||||||||||||||||||||||||
Sbjct  712    TGTTTCCATTCTGTCTTTGAGGGAATTGCCATTGGCGTCGCAGATTCAAAAGCCGATGCT  771

Query  661    TGGAGAGCTCTTTGGACTGTCTGTCTACACAAGATATTTGCTGCTATTGCAATGGGAATA  720
              ||||||||||||||||||||||||||||||||||||||||||||||||||||||||||||
Sbjct  772    TGGAGAGCTCTTTGGACTGTCTGTCTACACAAGATATTTGCTGCTATTGCAATGGGAATA  831

Query  721    GCTCTACTTAGGATGATCCCAAATCGTCCGTTGTTATCTTGTGCAGCATATGCTTTTGCC  780
              ||||||||||||||||||||||||||||||||||||||||||||||||||||||||||||
Sbjct  832    GCTCTACTTAGGATGATCCCAAATCGTCCGTTGTTATCTTGTGCAGCATATGCTTTTGCC  891

Query  781    TTTGCCATCTCTAGTCCAATTGGTGTTGCCATTGGAATCATAATTGATGCTACAACTCAA  840
              ||||||||||||||||||||||||||||||||||||||||||||||||||||||||||||
Sbjct  892    TTTGCCATCTCTAGTCCAATTGGTGTTGCCATTGGAATCATAATTGATGCTACAACTCAA  951

Query  841    GGGGTTGTTGCAGACTGGATCTTTGCTATATCAATGGGATTGGCTTGTGGGGTTTTTGTC  900
              ||||||||||||||||||||||||||||||||||||||||||||||||||||||||||||
Sbjct  952    GGGGTTGTTGCAGACTGGATCTTTGCTATATCAATGGGATTGGCTTGTGGGGTTTTTGTC  1011

Query  901    TATGTATCCATTAATCATTTGCTTTCAAGAGGATACAAGCCTCAGAAAATGGTCTTAGTT  960
              ||||||||||||||||||||||||||||||||||||||||||||||||||||||||||||
Sbjct  1012   TATGTATCCATTAATCATTTGCTTTCAAGAGGATACAAGCCTCAGAAAATGGTCTTAGTT  1071

Query  961    GACAAGCCTCATTTCAAGTTTTTGGCTGTATTGTTAGGTGTTGGAGCGATTGCTGTGGTC  1020
              ||||||||||||||||||||||||||||||||||||||||||||||||||||||||||||
Sbjct  1072   GACAAGCCTCATTTCAAGTTTTTGGCTGTATTGTTAGGTGTTGGAGCGATTGCTGTGGTC  1131

Query  1021   ATGATATGGGACACTTGA  1038
              ||||||||||||||||||
Sbjct  1132   ATGATATGGGACACTTGA  1149
```

> *NtZIP11-like - XM_016650095**

```
TGCAGTACTACACCAAATTTGGTAACTACTACAGTATATAAAAAACACACACATCTCCAA
TCTTCCCTCAATGATTTTCACTAAACTCCAATGGCACGTTATCAACCCTTTCTTTTCGTT
ACTCTCTTTCTTTTTCTCATCATCTCCGCCGCTGCCCACGGCGGAGCTCACGACGGAGAT
GCTGATGCAGATTCCGACTCATCTGCACCCTCCGATAAACCCCATCTGAGATCTCGACCG
TTGGTTTTTGTAAAAATATGGTGTTTGATTATAGTGTTCGTAGGGACTTTTCTTGGAGGA
ATATCACCTTATTTTATGAAATGGAATGAAGGGTTTTTGGTTCTTGGAACACAATTTGCT
GTGGGGTGTTTTTAGGGACAGCACTTATGCATTTCTTGAGTGATTCAAATGAGACATTTG
GAGATTTGACGAATAAAGAATACCCTTTTGCTTTTATGTTGGCATGTGCTGGTTATTTGC
TTACTATGTTGGCTGATTGTGTTATCTGTTTTGTTTATGCCAAGCAGAATAACAGCAACG
ATGTTCAGCTCCAAGGTGATACTGAAAATGGAAAAGGCAATGGGATTGTCACACAAGGAC
AGTCACAGGTCTGTGATGGCAGAGAAGATTACTTTTCAAAAGCACCTCTTGCAACTGCAT
CTTCGCTTGGCGATAGCATCCTATTGATCGTGGCGCTGTGTTTCCATTCTGTCTTTGAGG
GAATTGCCATTGGCGTCGCAGATTCAAAAGCCGATGCTTGGAGAGCTCTTTGGACTGTCT
GTCTACACAAGATATTTGCTGCCATTGCAATGGGAATAGCTCTACTTAGGATGATCCCTA
ATCGTCCGCTGTTATCTTGTGCAGCATATGCTTTTGCCTTTGCCATCTCCAGTCCAATTG
GTGTTGCCATTGGAATCATAATTGATGCCACAACTCAAGGGGTTGTTGCAGACTGGATCT
TTGCAATATCAATGGGATTGGCTTGTGGGGTTTTTGTTTATGTATCCATTAATCATTTGC
TTTCAAGGGGATACAAACCTCAGAAAATGGTCTTAGTTGACAAGCCTCATTTCAAGTTTT
TGGCTGTCTTGTTAGGTGTTGGAGTGATTGCTGTGGTGATGATATGGGACACTTGATTTG
TTTGGCAGCAGTAGCAGAATAGTTACCTCTTTATTTTGAAAATTTATCCCATTGTTGTTT
CAAAAGGTAGGTAGGTGGTATACATGATATTGAGAGTGAAATGTACAGTACTAAAAGTTG
TGATTTTTCTGGGTTCTGTCTTTATCCCCTGTTATTTAAATGGAATTGATTTCAAGAAAC
CAAATATATTTGTGGATTATTGCCAAGTTT
```

PREDICTED: Nicotiana tabacum **zinc transporter 11-like** (LOC107823460), mRNA
Sequence ID: XM_016650095.1Length: 1351Number of Matches: 1
Related Information
Gene-associated gene details
Range 1: 1 to 1351GenBankGraphics Next Match Previous Match First Match

Alignment statistics for match #1

Score	Expect	Identities	Gaps	Strand	Frame

2428 bits(2692) 0.0() 1350/1351(99%) 1/1351(0%) Plus/Plus

```
Query  1     TGCAGTACTACACCAAATTTGGTAACTACTACAGTATATAAAAAACACACACATCTCCAA  60
             ||||||||||||||||||||||||||||||||||||||||||||||||||||||||||||
Sbjct  1     TGCAGTACTACACCAAATTTGGTAACTACTACAGTATATAAAAAACACACACATCTCCAA  60

Query  61    TCTTCCCTCAATGATTTTCACTAAACTCCAATGGCACGTTATCAACCCTTTCTTTTCGTT  120
             ||||||||||||||||||||||||||||||||||||||||||||||||||||||||||||
Sbjct  61    TCTTCCCTCAATGATTTTCACTAAACTCCAATGGCACGTTATCAACCCTTTCTTTTCGTT  120

Query  121   ACTCTCTTTCTTTTTCTCATCATCTCCGCCGCTGCCCACGGCGGAGCTCACGACGGAGAT  180
             ||||||||||||||||||||||||||||||||||||||||||||||||||||||||||||
Sbjct  121   ACTCTCTTTCTTTTTCTCATCATCTCCGCCGCTGCCCACGGCGGAGCTCACGACGGAGAT  180

Query  181   GCTGATGCAGATTCCGACTCATCTGCACCCTCCGATAAACCCCATCTGAGATCTCGACCG  240
             ||||||||||||||||||||||||||||||||||||||||||||||||||||||||||||
Sbjct  181   GCTGATGCAGATTCCGACTCATCTGCACCCTCCGATAAACCCCATCTGAGATCTCGACCG  240

Query  241   TTGGTTTTTGTAAAAATATGGTGTTTGATTATAGTGTTCGTAGGGACTTTTCTTGGAGGA  300
             ||||||||||||||||||||||||||||||||||||||||||||||||||||||||||||
Sbjct  241   TTGGTTTTTGTAAAAATATGGTGTTTGATTATAGTGTTCGTAGGGACTTTTCTTGGAGGA  300

Query  301   ATATCACCTTATTTTATGAAATGGAATGAAGGGTTTTTGGTTCTTGGAACACAATTTGCT  360
             ||||||||||||||||||||||||||||||||||||||||||||||||||||||||||||
Sbjct  301   ATATCACCTTATTTTATGAAATGGAATGAAGGGTTTTTGGTTCTTGGAACACAATTTGCT  360

Query  361   G-TGGGGTGTTTTTAGGGACAGCACTTATGCATTTCTTGAGTGATTCAAATGAGACATTT  419
             | ||||||||||||||||||||||||||||||||||||||||||||||||||||||||||
Sbjct  361   GGTGGGGTGTTTTTAGGGACAGCACTTATGCATTTCTTGAGTGATTCAAATGAGACATTT  420

Query  420   GGAGATTTGACGAATAAAGAATACCCTTTTGCTTTTATGTTGGCATGTGCTGGTTATTTG  479
             ||||||||||||||||||||||||||||||||||||||||||||||||||||||||||||
Sbjct  421   GGAGATTTGACGAATAAAGAATACCCTTTTGCTTTTATGTTGGCATGTGCTGGTTATTTG  480

Query  480   CTTACTATGTTGGCTGATTGTGTTATCTGTTTTGTTTATGCCAAGCAGAATAACAGCAAC  539
             ||||||||||||||||||||||||||||||||||||||||||||||||||||||||||||
Sbjct  481   CTTACTATGTTGGCTGATTGTGTTATCTGTTTTGTTTATGCCAAGCAGAATAACAGCAAC  540

Query  540   GATGTTCAGCTCCAAGGTGATACTGAAAATGGAAAAGGCAATGGGATTGTCACACAAGGA  599
             ||||||||||||||||||||||||||||||||||||||||||||||||||||||||||||
Sbjct  541   GATGTTCAGCTCCAAGGTGATACTGAAAATGGAAAAGGCAATGGGATTGTCACACAAGGA  600

Query  600   CAGTCACAGGTCTGTGATGGCAGAGAAGATTACTTTTCAAAAGCACCTCTTGCAACTGCA  659
             ||||||||||||||||||||||||||||||||||||||||||||||||||||||||||||
Sbjct  601   CAGTCACAGGTCTGTGATGGCAGAGAAGATTACTTTTCAAAAGCACCTCTTGCAACTGCA  660

Query  660   TCTTCGCTTGGCGATAGCATCCTATTGATCGTGGCGCTGTGTTTCCATTCTGTCTTTGAG  719
             ||||||||||||||||||||||||||||||||||||||||||||||||||||||||||||
Sbjct  661   TCTTCGCTTGGCGATAGCATCCTATTGATCGTGGCGCTGTGTTTCCATTCTGTCTTTGAG  720

Query  720   GGAATTGCCATTGGCGTCGCAGATTCAAAAGCCGATGCTTGGAGAGCTCTTTGGACTGTC  779
             ||||||||||||||||||||||||||||||||||||||||||||||||||||||||||||
Sbjct  721   GGAATTGCCATTGGCGTCGCAGATTCAAAAGCCGATGCTTGGAGAGCTCTTTGGACTGTC  780

Query  780   TGTCTACACAAGATATTTGCTGCCATTGCAATGGGAATAGCTCTACTTAGGATGATCCCT  839
             ||||||||||||||||||||||||||||||||||||||||||||||||||||||||||||
Sbjct  781   TGTCTACACAAGATATTTGCTGCCATTGCAATGGGAATAGCTCTACTTAGGATGATCCCT  840

Query  840   AATCGTCCGCTGTTATCTTGTGCAGCATATGCTTTTGCCTTTGCCATCTCCAGTCCAATT  899
             ||||||||||||||||||||||||||||||||||||||||||||||||||||||||||||
Sbjct  841   AATCGTCCGCTGTTATCTTGTGCAGCATATGCTTTTGCCTTTGCCATCTCCAGTCCAATT  900

Query  900   GGTGTTGCCATTGGAATCATAATTGATGCCACAACTCAAGGGGTTGTTGCAGACTGGATC  959
             ||||||||||||||||||||||||||||||||||||||||||||||||||||||||||||
Sbjct  901   GGTGTTGCCATTGGAATCATAATTGATGCCACAACTCAAGGGGTTGTTGCAGACTGGATC  960

Query  960   TTTGCAATATCAATGGGATTGGCTTGTGGGGTTTTTGTTTATGTATCCATTAATCATTTG  1019
             ||||||||||||||||||||||||||||||||||||||||||||||||||||||||||||
Sbjct  961   TTTGCAATATCAATGGGATTGGCTTGTGGGGTTTTTGTTTATGTATCCATTAATCATTTG  1020

Query  1020  CTTTCAAGGGGATACAAACCTCAGAAAATGGTCTTAGTTGACAAGCCTCATTTCAAGTTT  1079
             ||||||||||||||||||||||||||||||||||||||||||||||||||||||||||||
Sbjct  1021  CTTTCAAGGGGATACAAACCTCAGAAAATGGTCTTAGTTGACAAGCCTCATTTCAAGTTT  1080

Query  1080  TTGGCTGTCTTGTTAGGTGTTGGAGTGATTGCTGTGGTGATGATATGGGACACTTGATTT  1139
```

```
                ||||||||||||||||||||||||||||||||||||||||||||||||||||||||||||
Sbjct  1081   TTGGCTGTCTTGTTAGGTGTTGGAGTGATTGCTGTGGTGATGATATGGGACACTTGATTT  1140

Query  1140   GTTTGGCAGCAGTAGCAGAATAGTTACCTCTTTATTTTGAAAATTTATCCCATTGTTGTT  1199
              ||||||||||||||||||||||||||||||||||||||||||||||||||||||||||||
Sbjct  1141   GTTTGGCAGCAGTAGCAGAATAGTTACCTCTTTATTTTGAAAATTTATCCCATTGTTGTT  1200

Query  1200   TCAAAAGGTAGGTAGGTGGTATACATGATATTGAGAGTGAAATGTACAGTACTAAAAGTT  1259
              ||||||||||||||||||||||||||||||||||||||||||||||||||||||||||||
Sbjct  1201   TCAAAAGGTAGGTAGGTGGTATACATGATATTGAGAGTGAAATGTACAGTACTAAAAGTT  1260

Query  1260   GTGATTTTTCTGGGTTCTGTCTTTATCCCCTGTTATTTAAATGGAATTGATTTCAAGAAA  1319
              ||||||||||||||||||||||||||||||||||||||||||||||||||||||||||||
Sbjct  1261   GTGATTTTTCTGGGTTCTGTCTTTATCCCCTGTTATTTAAATGGAATTGATTTCAAGAAA  1320

Query  1320   CCAAATATATTTGTGGATTATTGCCAAGTTT  1350
              |||||||||||||||||||||||||||||||
Sbjct  1321   CCAAATATATTTGTGGATTATTGCCAAGTTT  1351
```

> *NtNramp2-like* - XM_016621575

ATGAGTTCTCCATCACCGAAGCACAATGGCACACGAGAATCGAATCGTCTATTATCAGAT
GATTATGATGATCAGGCTAAAGATAAAATCTTGGCCATTGAAATTGAGAAAGCTGACGCC
GGTGATGTTCCAGGGGTGGTCCCACCTTTTTCTTGGAGGAAGCTATGGGAGTTTACAGGA
CCTGGGTTTTTAATGAGCGTTGCATTTCTTGATCCTGGAAATTTGGAAGGAGATTTGCAG
GCTGGAGCCATTGCGGGTTACTCGTTACTTTGGTTGCTTATGTGGTCCACTTTTATGGGC
TTGCTTATACAGCTATTGTCTCTTAGATTGGGTGTTGCTACGGGCCGACACTTGGCTGAG
ATCTGCCGGGAGGAATATCCAAATTGGGTCAGGATTTTGCTTTGGTTAATGGCGGAGGTG
GCTTTGATTGGAGCTGATATTCAGGAAGTTATAGGAAGTGCCATTGCTATTAAGATACTT
AGTTGTGGGGTTATTCCTCTTTGGGGTGGTGTCCTCATTACTGCTTCAGATTGCTTCATC
TTCTTGTTTCTGGAAAATTATGGAGTAAGGAAACTAGAAGCGTTTTTTGCTGTTCTAATT
TCCACTATGGCATTATCTTTCGCCTGGATGTTTGCTGAGACTAGGCCTAATGGCAAGGAA
CTCATAATAGGTCAGAAAACTTGTTTTCACATGAGCATTTCCTTACCTCTGTGGTCAGTA
TCAATTCTGCGCCTACGTCTTTTGCTACCAAGACTCAGCTCAAAGACAATTCAGAAAGCT
GTTGGAGTTGTTGGTTGTGTCATAACTCCTCACAATGTGTTCCTATACTCTGCTTTGGTG
CAGACAAGGAAAATTGATCCCAAAAAGAAAGAAAAAGTTCAAGAGGCATTGAACTACTAT
ACAATCGAGTCATCAATTGCTGTTTTTGTTTCATTCTCTATCAATTTGATGGTAACAACG
GTCTTTGCTAAAGGTTTCTATGGAACTCTACAAGCGCATAGCATAGGGCTAGTAAATGCT
GGGCAATATCTTCAAGAAAGATATGGTGGAGGGTTGTTTCCAATTCTCTACATATGGGGC
ATTGGCCTTTTGGCCGCTGGTCAAAGTAGCACGATGACTGGTACTTATGCTGGTCAGTTT
ATTATGGGAGGTTTTCTGAATCTGCGGATGAAGAAATGGATAAGGTCAGTGATTACAAGG
AGTTGTGCTATCGTGCCAACTATAATTGTGGCCATTTATTTCAACAGATCTGAGGATTCA
CTTGATGTATTAAACGAATGGCTAAATGTGCTACAAGGAATGGTGATCCCATTTGCTATC
ATTCCTCTTCTAACATTGGTGTCAAATGAGCAGATCATGGGAGTCTTCAAAATTGGGAAG
CTTATGGAGAGAACTGTTTGGACAGTAGCTGCTCTGGTAATAATGATAAATGGATATGTT
ATGTTGAGCTTCTTCCTTTCTGAAGTTAATGGCATGCTGTTTGGTTTAGTGGTTTGCATG
GGAGCTTCAGCATATGTGGCTTTTCTTGTATATCTCATTTCCCAGAGTCATAACCAGGCG
GAGTTGAATGGTTTTACTCACCTTACAAATTGA

PREDICTED: Nicotiana tabacum **metal transporter Nramp2-like** (LOC107798557), mRNA
Sequence ID: XM_016621570.1Length: 2224Number of Matches: 2
Range 1: 1019 to 1878GenBankGraphics Next Match Previous Match First Match

Alignment statistics for match #1

Score	Expect	Identities	Gaps	Strand	Frame
1546 bits(1714)	0.0()	859/860(99%)	0/860(0%)	Plus/Plus	

```
Query  734    TACGTCTTTTGCTACCAAGACTCAGCTCAAAGACAATTCAGAAAGCTGTTGGAGTTGTTG  793
              || |||||||||||||||||||||||||||||||||||||||||||||||||||||||||
Sbjct  1019   TAGGTCTTTTGCTACCAAGACTCAGCTCAAAGACAATTCAGAAAGCTGTTGGAGTTGTTG  1078

Query  794    GTTGTGTCATAACTCCTCACAATGTGTTCCTATACTCTGCTTTGGTGCAGACAAGGAAAA  853
              ||||||||||||||||||||||||||||||||||||||||||||||||||||||||||||
```

```
Sbjct  1079  GTTGTGTCATAACTCCTCACAATGTGTTCCTATACTCTGCTTTGGTGCAGACAAGGAAAA  1138

Query  854   TTGATCCCAAAAAGAAAGAAAAAGTTCAAGAGGCATTGAACTACTATACAATCGAGTCAT  913
             ||||||||||||||||||||||||||||||||||||||||||||||||||||||||||||
Sbjct  1139  TTGATCCCAAAAAGAAAGAAAAAGTTCAAGAGGCATTGAACTACTATACAATCGAGTCAT  1198

Query  914   CAATTGCTGTTTTTGTTTCATTCTCTATCAATTTGATGGTAACAACGGTCTTTGCTAAAG  973
             ||||||||||||||||||||||||||||||||||||||||||||||||||||||||||||
Sbjct  1199  CAATTGCTGTTTTTGTTTCATTCTCTATCAATTTGATGGTAACAACGGTCTTTGCTAAAG  1258

Query  974   GTTTCTATGGAACTCTACAAGCGCATAGCATAGGGCTAGTAAATGCTGGGCAATATCTTC  1033
             ||||||||||||||||||||||||||||||||||||||||||||||||||||||||||||
Sbjct  1259  GTTTCTATGGAACTCTACAAGCGCATAGCATAGGGCTAGTAAATGCTGGGCAATATCTTC  1318

Query  1034  AAGAAAGATATGGTGGAGGGTTGTTTCCAATTCTCTACATATGGGGCATTGGCCTTTTGG  1093
             ||||||||||||||||||||||||||||||||||||||||||||||||||||||||||||
Sbjct  1319  AAGAAAGATATGGTGGAGGGTTGTTTCCAATTCTCTACATATGGGGCATTGGCCTTTTGG  1378

Query  1094  CCGCTGGTCAAAGTAGCACGATGACTGGTACTTATGCTGGTCAGTTTATTATGGGAGGTT  1153
             ||||||||||||||||||||||||||||||||||||||||||||||||||||||||||||
Sbjct  1379  CCGCTGGTCAAAGTAGCACGATGACTGGTACTTATGCTGGTCAGTTTATTATGGGAGGTT  1438

Query  1154  TTCTGAATCTGCGGATGAAGAAATGGATAAGGTCAGTGATTACAAGGAGTTGTGCTATCG  1213
             ||||||||||||||||||||||||||||||||||||||||||||||||||||||||||||
Sbjct  1439  TTCTGAATCTGCGGATGAAGAAATGGATAAGGTCAGTGATTACAAGGAGTTGTGCTATCG  1498

Query  1214  TGCCAACTATAATTGTGGCCATTTATTTCAACAGATCTGAGGATTCACTTGATGTATTAA  1273
             ||||||||||||||||||||||||||||||||||||||||||||||||||||||||||||
Sbjct  1499  TGCCAACTATAATTGTGGCCATTTATTTCAACAGATCTGAGGATTCACTTGATGTATTAA  1558

Query  1274  ACGAATGGCTAAATGTGCTACAAGGAATGGTGATCCCATTTGCTATCATTCCTCTTCTAA  1333
             ||||||||||||||||||||||||||||||||||||||||||||||||||||||||||||
Sbjct  1559  ACGAATGGCTAAATGTGCTACAAGGAATGGTGATCCCATTTGCTATCATTCCTCTTCTAA  1618

Query  1334  CATTGGTGTCAAATGAGCAGATCATGGGAGTCTTCAAAATTGGGAAGCTTATGGAGAGAA  1393
             ||||||||||||||||||||||||||||||||||||||||||||||||||||||||||||
Sbjct  1619  CATTGGTGTCAAATGAGCAGATCATGGGAGTCTTCAAAATTGGGAAGCTTATGGAGAGAA  1678

Query  1394  CTGTTTGGACAGTAGCTGCTCTGGTAATAATGATAAATGGATATGTTATGTTGAGCTTCT  1453
             ||||||||||||||||||||||||||||||||||||||||||||||||||||||||||||
Sbjct  1679  CTGTTTGGACAGTAGCTGCTCTGGTAATAATGATAAATGGATATGTTATGTTGAGCTTCT  1738

Query  1454  TCCTTTCTGAAGTTAATGGCATGCTGTTTGGTTTAGTGGTTTGCATGGGAGCTTCAGCAT  1513
             ||||||||||||||||||||||||||||||||||||||||||||||||||||||||||||
Sbjct  1739  TCCTTTCTGAAGTTAATGGCATGCTGTTTGGTTTAGTGGTTTGCATGGGAGCTTCAGCAT  1798

Query  1514  ATGTGGCTTTTCTTGTATATCTCATTTCCCAGAGTCATAACCAGGCGGAGTTGAATGGTT  1573
             ||||||||||||||||||||||||||||||||||||||||||||||||||||||||||||
Sbjct  1799  ATGTGGCTTTTCTTGTATATCTCATTTCCCAGAGTCATAACCAGGCGGAGTTGAATGGTT  1858

Query  1574  TTACTCACCTTACAAATTGA  1593
             ||||||||||||||||||||
Sbjct  1859  TTACTCACCTTACAAATTGA  1878
```

> *NtNramp3-like* - XM_009618066

ATGCCTCCACACGATGACGAACAGCAACAATTGCTAGCCAACCGATTACTCGATTCCAAC
GAGGAAGAAACAGCGTATGACTACTCCGACAAAGTTCATATCATCGGAGTCGACGAACAC
GACGGTGAAGATTTCACCGAAGCTCCTCCTTTTTCTTGGAAAAAGCTATGGCTTTTTACT
GGGCCGGGTTTTTAATGAGCATAGCGTTTTTGGATCCGGGAAACCTTGAAGGAGATCTT
CAGGCGGGTGCAATCGCTGGGTACTCTCTGTTATGGCTACTCTTTTGGGCTACGGCTATT
GGGCTACTTGTTCAGCTTTTATCGGCTCGTTTGGGAGTGGCCACCTCTAGACATTTGGCT
GAGTTGTGTAGGGATGAATATCCTACATGGGCTAGGTTGCTTTTATGGATCATGGCTGAA
TTGGCTTTAATTGGGGCTGATATTCAAGAGGTTATTGGCAGTGCTATTGCTATAAAGATT
TTGAGTCGAGGATTCTTGCCTCTCTGGTCTGGTGTTGTCATTACCGCTCTTGATTGCTTT
ATATTCTTATTTCTTGAGAACTATGGTGTGCGAAAGCTGGAAGCACTCTTTGCCGTCCTT
ATTGCAGTTATGGCAGTCTCATTTGCATGGATGTTTGGAGAAACAAAACCTAATGGAGTT
GAACTTCTTGTTGGTATTGTGGTTCCAAAACTGAGCTCCAAGACAATAAAGCAGGCAGTG
GGAATTGTAGGGTGTGTTATCATGCCTCACAATGTGTTTCTACATTCTGCTCTAGTGCAG
TCCAGAGAGATTGACCACCATAGGGTTGGAAGAGTTCGAGAAGCACTCAAATACTACTCC
ATAGAGTCGACAGCTGCTTTGGCAATTTCATTCATTATCAATCTGTTTGTCACAACAGTG

TTCGCAAAGTCATTTTATGGTAGTGAAATAGCCAATAGCATTGGCCTAGAAAATGCAGGT
CAATATCTTCAGGAAAAGTATGGCGGAGGAGTGTTCCCCATCCTTTATATCTGGGCTATT
GGATTGTTGGCTGCTGGACAGAGTAGCACTATAACTGGCACTTATGCTGGGCAATTTATT
ATGGGAGGTTTTCTGCACATGAGGTTGAAAAAATGGCAGAGAGCGTTAATAACAAGAAGT
TGTGCTATCATCCCAACTCTGATCGTTGCTCTTGCTTTTGACACCTCTGAGAAGTCATTA
GATGTTCTCAACGAGTGGCTTAATGTTCTTCAGTCTGTTCAAATCCCTTTTGCCCTGATC
CCCCTTCTTTGTCTTGTATCCAAAGAAGAAATCATGGGTGTTTTCAAAATTGGCTCTACT
CTAAAGGTGATATCATGGCTTGTGGCTGCGCTGGTGATACTGATTAATGGCTATCTTTTG
ATGGACTCCTTATCTTCTGCAGTCAGTGGGGTGTTGTTTACATCTGTTGTATTTGCATTT
ACGGGTGGATACGTTGCTTTTATTGTATACCTCATTTTACGGGGAATTACCTTCCCCAAT
TGGTTTGTAAAAAACAAGAGTATCACCAGCATAGAGAATTGA

PREDICTED: Nicotiana tomentosiformis **metal transporter Nramp3-like** (LOC104108913), mRNA
Sequence ID: XM_009618066.2Length: 1887Number of Matches: 1
Range 1: 141 to 1682GenBankGraphics Next Match Previous Match First Match

Alignment statistics for match #1

Score	Expect	Identities	Gaps	Strand	Frame
2776 bits(3078)	0.0()	1541/1542(99%)	0/1542(0%)	Plus/Plus	

```
Query  1    ATGCCTCCACACGATGACGAACAGCAACAATTGCTAGCCAACCGATTACTCGATTCCAAC  60
            |||||||||||||||| |||||||||||||||||||||||||||||||||||||||||||
Sbjct  141  ATGCCTCCACACGATGGCGAACAGCAACAATTGCTAGCCAACCGATTACTCGATTCCAAC  200

Query  61   GAGGAAGAAACAGCGTATGACTACTCCGACAAAGTTCATATCATCGGAGTCGACGAACAC  120
            ||||||||||||||||||||||||||||||||||||||||||||||||||||||||||||
Sbjct  201  GAGGAAGAAACAGCGTATGACTACTCCGACAAAGTTCATATCATCGGAGTCGACGAACAC  260

Query  121  GACGGTGAAGATTTCACCGAAGCTCCTCCTTTTTCTTGGAAAAAGCTATGGCTTTTTACT  180
            ||||||||||||||||||||||||||||||||||||||||||||||||||||||||||||
Sbjct  261  GACGGTGAAGATTTCACCGAAGCTCCTCCTTTTTCTTGGAAAAAGCTATGGCTTTTTACT  320

Query  181  GGGCCGGGTTTTTTAATGAGCATAGCGTTTTTGGATCCGGGAAACCTTGAAGGAGATCTT  240
            ||||||||||||||||||||||||||||||||||||||||||||||||||||||||||||
Sbjct  321  GGGCCGGGTTTTTTAATGAGCATAGCGTTTTTGGATCCGGGAAACCTTGAAGGAGATCTT  380

Query  241  CAGGCGGGTGCAATCGCTGGGTACTCTCTGTTATGGCTACTCTTTTGGGCTACGGCTATT  300
            ||||||||||||||||||||||||||||||||||||||||||||||||||||||||||||
Sbjct  381  CAGGCGGGTGCAATCGCTGGGTACTCTCTGTTATGGCTACTCTTTTGGGCTACGGCTATT  440

Query  301  GGGCTACTTGTTCAGCTTTTATCGGCTCGTTTGGGAGTGGCCACCTCTAGACATTTGGCT  360
            ||||||||||||||||||||||||||||||||||||||||||||||||||||||||||||
Sbjct  441  GGGCTACTTGTTCAGCTTTTATCGGCTCGTTTGGGAGTGGCCACCTCTAGACATTTGGCT  500

Query  361  GAGTTGTGTAGGGATGAATATCCTACATGGGCTAGGTTGCTTTTATGGATCATGGCTGAA  420
            ||||||||||||||||||||||||||||||||||||||||||||||||||||||||||||
Sbjct  501  GAGTTGTGTAGGGATGAATATCCTACATGGGCTAGGTTGCTTTTATGGATCATGGCTGAA  560

Query  421  TTGGCTTTAATTGGGGCTGATATTCAAGAGGTTATTGGCAGTGCTATTGCTATAAAGATT  480
            ||||||||||||||||||||||||||||||||||||||||||||||||||||||||||||
Sbjct  561  TTGGCTTTAATTGGGGCTGATATTCAAGAGGTTATTGGCAGTGCTATTGCTATAAAGATT  620

Query  481  TTGAGTCGAGGATTCTTGCCTCTCTGGTCTGGTGTTGTCATTACCGCTCTTGATTGCTTT  540
            ||||||||||||||||||||||||||||||||||||||||||||||||||||||||||||
Sbjct  621  TTGAGTCGAGGATTCTTGCCTCTCTGGTCTGGTGTTGTCATTACCGCTCTTGATTGCTTT  680

Query  541  ATATTCTTATTTCTTGAGAACTATGGTGTGCGAAAGCTGGAAGCACTCTTTGCCGTCCTT  600
            ||||||||||||||||||||||||||||||||||||||||||||||||||||||||||||
Sbjct  681  ATATTCTTATTTCTTGAGAACTATGGTGTGCGAAAGCTGGAAGCACTCTTTGCCGTCCTT  740

Query  601  ATTGCAGTTATGGCAGTCTCATTTGCATGGATGTTTGGAGAAACAAAACCTAATGGAGTT  660
            ||||||||||||||||||||||||||||||||||||||||||||||||||||||||||||
Sbjct  741  ATTGCAGTTATGGCAGTCTCATTTGCATGGATGTTTGGAGAAACAAAACCTAATGGAGTT  800

Query  661  GAACTTCTTGTTGGTATTGTGGTTCCAAAACTGAGCTCCAAGACAATAAAGCAGGCAGTG  720
            ||||||||||||||||||||||||||||||||||||||||||||||||||||||||||||
Sbjct  801  GAACTTCTTGTTGGTATTGTGGTTCCAAAACTGAGCTCCAAGACAATAAAGCAGGCAGTG  860
```

```
Query  721   GGAATTGTAGGGTGTGTTATCATGCCTCACAATGTGTTTCTACATTCTGCTCTAGTGCAG  780
             ||||||||||||||||||||||||||||||||||||||||||||||||||||||||||||
Sbjct  861   GGAATTGTAGGGTGTGTTATCATGCCTCACAATGTGTTTCTACATTCTGCTCTAGTGCAG  920

Query  781   TCCAGAGAGATTGACCACCATAGGGTTGGAAGAGTTCGAGAAGCACTCAAATACTACTCC  840
             ||||||||||||||||||||||||||||||||||||||||||||||||||||||||||||
Sbjct  921   TCCAGAGAGATTGACCACCATAGGGTTGGAAGAGTTCGAGAAGCACTCAAATACTACTCC  980

Query  841   ATAGAGTCGACAGCTGCTTTGGCAATTTCATTCATTATCAATCTGTTTGTCACAACAGTG  900
             ||||||||||||||||||||||||||||||||||||||||||||||||||||||||||||
Sbjct  981   ATAGAGTCGACAGCTGCTTTGGCAATTTCATTCATTATCAATCTGTTTGTCACAACAGTG  1040

Query  901   TTCGCAAAGTCATTTTATGGTAGTGAAATAGCCAATAGCATTGGCCTAGAAAATGCAGGT  960
             ||||||||||||||||||||||||||||||||||||||||||||||||||||||||||||
Sbjct  1041  TTCGCAAAGTCATTTTATGGTAGTGAAATAGCCAATAGCATTGGCCTAGAAAATGCAGGT  1100

Query  961   CAATATCTTCAGGAAAAGTATGGCGGAGGAGTGTTCCCCATCCTTTATATCTGGGCTATT  1020
             ||||||||||||||||||||||||||||||||||||||||||||||||||||||||||||
Sbjct  1101  CAATATCTTCAGGAAAAGTATGGCGGAGGAGTGTTCCCCATCCTTTATATCTGGGCTATT  1160

Query  1021  GGATTGTTGGCTGCTGGACAGAGTAGCACTATAACTGGCACTTATGCTGGGCAATTTATT  1080
             ||||||||||||||||||||||||||||||||||||||||||||||||||||||||||||
Sbjct  1161  GGATTGTTGGCTGCTGGACAGAGTAGCACTATAACTGGCACTTATGCTGGGCAATTTATT  1220

Query  1081  ATGGGAGGTTTTCTGCACATGAGGTTGAAAAAATGGCAGAGAGCGTTAATAACAAGAAGT  1140
             ||||||||||||||||||||||||||||||||||||||||||||||||||||||||||||
Sbjct  1221  ATGGGAGGTTTTCTGCACATGAGGTTGAAAAAATGGCAGAGAGCGTTAATAACAAGAAGT  1280

Query  1141  TGTGCTATCATCCCAACTCTGATCGTTGCTCTTGCTTTTGACACCTCTGAGAAGTCATTA  1200
             ||||||||||||||||||||||||||||||||||||||||||||||||||||||||||||
Sbjct  1281  TGTGCTATCATCCCAACTCTGATCGTTGCTCTTGCTTTTGACACCTCTGAGAAGTCATTA  1340

Query  1201  GATGTTCTCAACGAGTGGCTTAATGTTCTTCAGTCTGTTCAAATCCCTTTTGCCCTGATC  1260
             ||||||||||||||||||||||||||||||||||||||||||||||||||||||||||||
Sbjct  1341  GATGTTCTCAACGAGTGGCTTAATGTTCTTCAGTCTGTTCAAATCCCTTTTGCCCTGATC  1400

Query  1261  CCCCTTCTTTGTCTTGTATCCAAAGAAGAAATCATGGGTGTTTTCAAAATTGGCTCTACT  1320
             ||||||||||||||||||||||||||||||||||||||||||||||||||||||||||||
Sbjct  1401  CCCCTTCTTTGTCTTGTATCCAAAGAAGAAATCATGGGTGTTTTCAAAATTGGCTCTACT  1460

Query  1321  CTAAAGGTGATATCATGGCTTGTGGCTGCGCTGGTGATACTGATTAATGGCTATCTTTTG  1380
             ||||||||||||||||||||||||||||||||||||||||||||||||||||||||||||
Sbjct  1461  CTAAAGGTGATATCATGGCTTGTGGCTGCGCTGGTGATACTGATTAATGGCTATCTTTTG  1520

Query  1381  ATGGACTCCTTATCTTCTGCAGTCAGTGGGGTGTTGTTTACATCTGTTGTATTTGCATTT  1440
             ||||||||||||||||||||||||||||||||||||||||||||||||||||||||||||
Sbjct  1521  ATGGACTCCTTATCTTCTGCAGTCAGTGGGGTGTTGTTTACATCTGTTGTATTTGCATTT  1580

Query  1441  ACGGGTGGATACGTTGCTTTTATTGTATACCTCATTTTACGGGGAATTACCTTCCCCAAT  1500
             ||||||||||||||||||||||||||||||||||||||||||||||||||||||||||||
Sbjct  1581  ACGGGTGGATACGTTGCTTTTATTGTATACCTCATTTTACGGGGAATTACCTTCCCCAAT  1640

Query  1501  TGGTTTGTAAAAAACAAGAGTATCACCAGCATAGAGAATTGA  1542
             ||||||||||||||||||||||||||||||||||||||||||
Sbjct  1641  TGGTTTGTAAAAAACAAGAGTATCACCAGCATAGAGAATTGA  1682
```

>*NtNramp6-like* - XM_016619903

ATGGCGGCGAACTCGTCCCCACAGCCGCAGTTTATGACAAACACTGCAAACAAGAACCTT
TCCAATCAGCCACTGATTGATGATATCGAATATGATCAGATTGTTGTACCCGATATAATT
TTTGTACTGTGCTTATGTTTTTCATTGTTGGATATTCCGTATTTTAGTTTGCCCGGTTGT
GAGAAAATTTGTAGGACTGGTAGATGGAAAAAGAAAAGCTGGAAGAACATATTTTCATAT
GTTGGTCCTGGTTTCCTTGTTTGTATTGCCTATATCGATCCTGGAAATTTTCAAACTGAT
TTACAAGCTGGAGCTCAATACAAATATGGGGTGTGTATCTGTGTTGCTCTATCTTTCATA
ATCAAGTGTGCAGATTTGCTTTGGATAATCTTACTGGCGTCCTTTGCTGCTCTTGTGATC
CAATCCTTGGCAGCGAATCTAGGGGTGGTTACAGGGAAGCATTTAGCAGAGCATTGTAGA
AAGGAGTACCCAAAGGTGCCAAATTTCATCCTATGGATCATAGCAGAAATCGCTATTGTG
GCATGTGACATTCCTGAGGTTATTGGGACAGCATTTGCTTTAAACATGCTGTTCAGAATA
CCAATATGGTGTGGTGTGCTGATCACAGGGCTGAGTACTTTGGGTCTACTTCTATTACAA
CAGTATGGGGTTCGGAAACTTGAATTCTTGATTGCATTCCTTGTACTTACCATAGCTGTA
TGCTTTTTTGTGGAGCTTGGATATGCAAAGCCGGAGTCTTCAGAAGTTCTCCATGGGCTT
TTTGTTCCTCAACTCAAAGGGAGTGGTGCAACTAAGCTTGCTATTTCCCTACTTGGTGCT

```
ATGGTTATGCCGCACAATCTTTTCCTCCATTCAGCCCTGGTGCTTTCCAGGAAAATTCCT
CGATCTGTCAATGGCATCAGAGACGCATGCAGATATTATCTAATCGAAAGTGGTCTGGCT
TTGATGGTGGCATTTCTTATCAACATATCAGTTATTTCAGTCAGTGGTGCTGTCTGCAAT
TCCTCAACTATGACCGCAGATGACCGGGAGAAGTGTGAGGACTTAGACCTGAACAAAGCC
TCTTTTTTACTCCAAGTATGTGATATCATACTGTCAGATAATTTCTATTCACTTAAATTC
TACGTTTTTCATGCTTTTTATAATTTTAATGTTTTAGGGAACTGGAGTTCCAAGCTATTT
GCAATTGCTTTACTAGCATCGGGACAGAGTTCGACAATAACTGGGACATACGCCGGGCAA
TACGTTATGCAGGGTTTTCTTGATTTACGGCTGAAGCCATGGATAAGGAACTTCTTAACT
CGTAGCTTAGCTATAGTCCCAAGTTTAATTGTTTCACTTATTGGAGGCTCTGCTGGGGCT
GGAGACTTGATCATTATTGCTTCGGTAAAGACTATGGGTAGGACTGACCACAGACTGGCT
CGGAGTGGAACAAACATGTGCATTTTTGAGCTGGCACAACTCGATTGTCATTTTCTAGAT
TTATTAAAACGGGGTAAGGGGGTTTGGGGCTTTCCCGCGGAGTTAATTCTGACCCACAGA
GTAATTAAGAGAAGGTTTTTGCTGGAGTTGATTCTGACCTTCCTGGAACTATGCTATATC
TTTTTAAGATCTCATAATATGATCTTATCTTTTGAGCTCCCCTTTGCTCTGATTCCATTG
CTCAAATTCACAAGCAGTAAAACCAAGATGGGTTCACATGTAAATCCAGTTGCGGTTTCA
GCAGCAACCTGGGTTATTGGCACATTAATCATGGGAATAAATATATATTATCTAGCAGAG
AAGTTGGTTACCTCTCTCAAGGATAGCCATTTAGGAAAGGCGGTTAAGGTTCTTTGTGGA
ATATTAGGCACCTTGTGTTTGTTAGTTTATCTGTGCAGTATCTTGTACTTGGCTATCCGA
AAAAATAAGGAAAGCACGCACCTTCTGGCGCTTACAGGGCAAGAAGGTTTACAAGTTTCT
GAATCAAACAATCTACCTAGAGAGGACATTCTGCGCATGCAGTTGCCTCAGCAGAGGACC
ACTAATTGA
```

PREDICTED: Nicotiana tabacum **metal transporter Nramp6-like** (LOC107797051), transcript variant X2, mRNA
Sequence ID: XM_016619903.1Length: 2010Number of Matches: 3
Range 1: 303 to 1146GenBankGraphics Next Match Previous Match First Match

Alignment statistics for match #1

Score	Expect	Identities	Gaps	Strand	Frame
1433 bits(1588)	0.0()	843/889(95%)	45/889(5%)	Plus/Plus	

```
Query  207    GAAAAAGAAAAGCTGGAAGAACATATTTTCATATGTTGGTCCTGGTTTCCTTGTTTGTAT  266
              || |||||||||||||||||||||||||||||||||||||||||||||||||||||||||
Sbjct  303    GAGAAAGAAAAGCTGGAAGAACATATTTTCATATGTTGGTCCTGGTTTCCTTGTTTGTAT  362

Query  267    TGCCTATATCGATCCTGGAAATTTTCAAACTGATTTACAAGCTGGAGCTCAATACAAATA  326
              ||||||||||||||||||||||||||||||||||||||||||||||||||||||||||||
Sbjct  363    TGCCTATATCGATCCTGGAAATTTTCAAACTGATTTACAAGCTGGAGCTCAATACAAATA  422

Query  327    TGGGGTGTGTATCTGTGTTGCTCTATCTTTCATAATCAAGTGTGCAGATTTGCTTTGGAT  386
              ||||                                             |||||||||||
Sbjct  423    TGGG---------------------------------------------TTGCTTTGGAT  437

Query  387    AATCTTACTGGCGTCCTTTGCTGCTCTTGTGATCCAATCCTTGGCAGCGAATCTAGGGGT  446
              ||||||||||||||||||||||||||||||||||||||||||||||||||||||||||||
Sbjct  438    AATCTTACTGGCGTCCTTTGCTGCTCTTGTGATCCAATCCTTGGCAGCGAATCTAGGGGT  497

Query  447    GGTTACAGGGAAGCATTTAGCAGAGCATTGTAGAAAGGAGTACCCAAAGGTGCCAAATTT  506
              ||||||||||||||||||||||||||||||||||||||||||||||||||||||||||||
Sbjct  498    GGTTACAGGGAAGCATTTAGCAGAGCATTGTAGAAAGGAGTACCCAAAGGTGCCAAATTT  557

Query  507    CATCCTATGGATCATAGCAGAAATCGCTATTGTGGCATGTGACATTCCTGAGGTTATTGG  566
              ||||||||||||||||||||||||||||||||||||||||||||||||||||||||||||
Sbjct  558    CATCCTATGGATCATAGCAGAAATCGCTATTGTGGCATGTGACATTCCTGAGGTTATTGG  617

Query  567    GACAGCATTTGCTTTAAACATGCTGTTCAGAATACCAATATGGTGTGGTGTGCTGATCAC  626
              ||||||||||||||||||||||||||||||||||||||||||||||||||||||||||||
Sbjct  618    GACAGCATTTGCTTTAAACATGCTGTTCAGAATACCAATATGGTGTGGTGTGCTGATCAC  677

Query  627    AGGGCTGAGTACTTTGGGTCTACTTCTATTACAACAGTATGGGGTTCGGAAACTTGAATT  686
              ||||||||||||||||||||||||||||||||||||||||||||||||||||||||||||
Sbjct  678    AGGGCTGAGTACTTTGGGTCTACTTCTATTACAACAGTATGGGGTTCGGAAACTTGAATT  737

Query  687    CTTGATTGCATTCCTTGTACTTACCATAGCTGTATGCTTTTTTGTGGAGCTTGGATATGC  746
              ||||||||||||||||||||||||||||||||||||||||||||||||||||||||||||
Sbjct  738    CTTGATTGCATTCCTTGTACTTACCATAGCTGTATGCTTTTTTGTGGAGCTTGGATATGC  797
```

```
Query  747   AAAGCCGGAGTCTTCAGAAGTTCTCCATGGGCTTTTTGTTCCTCAACTCAAAGGGAGTGG  806
             ||||||||||||||||||||||||||||||||||||||||||||||||||||||||||||
Sbjct  798   AAAGCCGGAGTCTTCAGAAGTTCTCCATGGGCTTTTTGTTCCTCAACTCAAAGGGAGTGG  857

Query  807   TGCAACTAAGCTTGCTATTTCCCTACTTGGTGCTATGGTTATGCCGCACAATCTTTTCCT  866
             ||||||||||||||||||||||||||||||||||||||||||||||||||||||||||||
Sbjct  858   TGCAACTAAGCTTGCTATTTCCCTACTTGGTGCTATGGTTATGCCGCACAATCTTTTCCT  917

Query  867   CCATTCAGCCCTGGTGCTTTCCAGGAAAATTCCTCGATCTGTCAATGGCATCAGAGACGC  926
             ||||||||||||||||||||||||||||||||||||||||||||||||||||||||||||
Sbjct  918   CCATTCAGCCCTGGTGCTTTCCAGGAAAATTCCTCGATCTGTCAATGGCATCAGAGACGC  977

Query  927   ATGCAGATATTATCTAATCGAAAGTGGTCTGGCTTTGATGGTGGCATTTCTTATCAACAT  986
             ||||||||||||||||||||||||||||||||||||||||||||||||||||||||||||
Sbjct  978   ATGCAGATATTATCTAATCGAAAGTGGTCTGGCTTTGATGGTGGCATTTCTTATCAACAT  1037

Query  987   ATCAGTTATTTCAGTCAGTGGTGCTGTCTGCAATTCCTCAACTATGACCGCAGATGACCG  1046
             ||||||||||||||||||||||||||||||||||||||||||||||||||||||||||||
Sbjct  1038  ATCAGTTATTTCAGTCAGTGGTGCTGTCTGCAATTCCTCAACTATGACCGCAGATGACCG  1097

Query  1047  GGAGAAGTGTGAGGACTTAGACCTGAACAAAGCCTCTTTTTTACTCCAA  1095
             |||||||||||||||||||||||||||||||||||||||||||||||||
Sbjct  1098  GGAGAAGTGTGAGGACTTAGACCTGAACAAAGCCTCTTTTTTACTCCAA  1146
```

> *NtMTP2-transcript variant-X1* - XM_016593653

ATGGTAGCTAGATTCATTTCAAGCAATCTAAATCGAATACGAAAAACGTATATAGCATCA
ACTATTAGATACAATAACAATTTCACTCCTCTATATCTATCTCCCAATTTGCAATTCGAT
AATGATGATTCAGCTACAAGTATTGGGAATCGCAGCTTTATGCTTCAGAAAAGATGGCAT
ATGGGGCATTCACATTCACATCATGACCATGACGACCTCCGCTCCGGTAAAGACGGCGAG
AGGATTTTCCGGCTTGGCCTCGCTGCTGATATTGGCCTCGCCGCCAGTAAAGCTTTCACA
GGTTATATATGCGGTAGCACCGCCATTATCGCTGATGCCGCCCATTCTATCTCCGATGTG
GTTCTGAGTGGAGTGGCATTGTTGTCGTTTAAAGCTGCAAGGGTTCCCAAGGACAAAGAA
CATCCTTATGGACATGGTAAATTTGAGACTCTTGGAGCTCTTGGAATTTCTGGTGTACTA
TTGGCTACTGCTGGAGGTATTGGATGGCATGCTTTAGATGTTTTGCTGGGACTATGGTCT
ACAGCGCCTGAAGTTGTTAATCAGTCATTGAGTCATTTGGACGTGCATGAGCAACATCAT
AGTGGACATCACCATGGAATAGATATGGATCACCCTATTCTTGCTTTGAATGTGACTATA
CTCTCTATAGCTGTTAAAGAAGGATTATACTGGATAACAAAGAGAGCGGGGGATAAGATT
GGCAGTGGACTGATGAAAGCCAATGCTTGGCATCATCGTGCTGATGCAGTATCCTCTGTC
GTTGCTCTCGTAGGAGTTGGTAATGACCTTTATACTCAAATTTTACCTACTTATGGCTGG
AGATAA

PREDICTED: Nicotiana tomentosiformis **metal tolerance protein 2** (LOC104099394), transcript variant X1, mRNA
Sequence ID: XM_018771850.1Length: 1805Number of Matches: 1
Range 1: 184 to 984GenBankGraphics Next Match Previous Match First Match

Alignment statistics for match #1

Score	Expect	Identities	Gaps	Strand	Frame
1440 bits(1596)	0.0()	800/801(99%)	0/801(0%)	Plus/Plus	

```
Query  1     ATGGTAGCTAGATTCATTTCAAGCAATCTAAATCGAATACGAAAAACGTATATAGCATCA  60
             ||||||||||||||||||||||||||||||||||||||||||||||||||||||||||||
Sbjct  184   ATGGTAGCTAGATTCATTTCAAGCAATCTAAATCGAATACGAAAAACGTATATAGCATCA  243

Query  61    ACTATTAGATACAATAACAATTTCACTCCTCTATATCTATCTCCCAATTTGCAATTCGAT  120
             ||||||||||||||||||||||||||||||||||||||||||||||||||||||||||||
Sbjct  244   ACTATTAGATACAATAACAATTTCACTCCTCTATATCTATCTCCCAATTTGCAATTCGAT  303

Query  121   AATGATGATTCAGCTACAAGTATTGGGAATCGCAGCTTTATGCTTCAGAAAAGATGGCAT  180
             ||||||||||||||||||||||||||||||||||||||||||||||||||||||||||||
Sbjct  304   AATGATGATTCAGCTACAAGTATTGGGAATCGCAGCTTTATGCTTCAGAAAAGATGGCAT  363

Query  181   ATGGGGCATTCACATTCACATCATGACCATGACGACCTCCGCTCCGGTAAAGACGGCGAG  240
             ||||||||||||||||||||||||||||||||||||||||||||||||||||||||||||
Sbjct  364   ATGGGGCATTCACATTCACATCATGACCATGACGACCTCCGCTCCGGTAAAGACGGCGAG  423
```

```
Query  241   AGGATTTTCCGGCTTGGCCTCGCTGCTGATATTGGCCTCGCCGCCAGTAAAGCTTTCACA  300
             ||||||||||||||||||||||||||||||||||||||||||||||||||||||||||||
Sbjct  424   AGGATTTTCCGGCTTGGCCTCGCTGCTGATATTGGCCTCGCCGCCAGTAAAGCTTTCACA  483

Query  301   GGTTATATATGCGGTAGCACCGCCATTATCGCTGATGCCGCCCATTCTATCTCCGATGTG  360
             ||||||||||||||||||||||||||||||||||||||||||||||||||||||||||||
Sbjct  484   GGTTATATATGCGGTAGCACCGCCATTATCGCTGATGCCGCCCATTCTATCTCCGATGTG  543

Query  361   GTTCTGAGTGGAGTGGCATTGTTGTCGTTTAAAGCTGCAAGGGTTCCCAAGGACAAAGAA  420
             ||| ||||||||||||||||||||||||||||||||||||||||||||||||||||||||
Sbjct  544   GTTTTGAGTGGAGTGGCATTGTTGTCGTTTAAAGCTGCAAGGGTTCCCAAGGACAAAGAA  603

Query  421   CATCCTTATGGACATGGTAAATTTGAGACTCTTGGAGCTCTTGGAATTTCTGGTGTACTA  480
             ||||||||||||||||||||||||||||||||||||||||||||||||||||||||||||
Sbjct  604   CATCCTTATGGACATGGTAAATTTGAGACTCTTGGAGCTCTTGGAATTTCTGGTGTACTA  663

Query  481   TTGGCTACTGCTGGAGGTATTGGATGGCATGCTTTAGATGTTTTGCTGGGACTATGGTCT  540
             ||||||||||||||||||||||||||||||||||||||||||||||||||||||||||||
Sbjct  664   TTGGCTACTGCTGGAGGTATTGGATGGCATGCTTTAGATGTTTTGCTGGGACTATGGTCT  723

Query  541   ACAGCGCCTGAAGTTGTTAATCAGTCATTGAGTCATTTGGACGTGCATGAGCAACATCAT  600
             ||||||||||||||||||||||||||||||||||||||||||||||||||||||||||||
Sbjct  724   ACAGCGCCTGAAGTTGTTAATCAGTCATTGAGTCATTTGGACGTGCATGAGCAACATCAT  783

Query  601   AGTGGACATCACCATGGAATAGATATGGATCACCCTATTCTTGCTTTGAATGTGACTATA  660
             ||||||||||||||||||||||||||||||||||||||||||||||||||||||||||||
Sbjct  784   AGTGGACATCACCATGGAATAGATATGGATCACCCTATTCTTGCTTTGAATGTGACTATA  843

Query  661   CTCTCTATAGCTGTTAAAGAAGGATTATACTGGATAACAAAGAGAGCGGGGGATAAGATT  720
             ||||||||||||||||||||||||||||||||||||||||||||||||||||||||||||
Sbjct  844   CTCTCTATAGCTGTTAAAGAAGGATTATACTGGATAACAAAGAGAGCGGGGGATAAGATT  903

Query  721   GGCAGTGGACTGATGAAAGCCAATGCTTGGCATCATCGTGCTGATGCAGTATCCTCTGTC  780
             ||||||||||||||||||||||||||||||||||||||||||||||||||||||||||||
Sbjct  904   GGCAGTGGACTGATGAAAGCCAATGCTTGGCATCATCGTGCTGATGCAGTATCCTCTGTC  963

Query  781   GTTGCTCTCGTAGGAGTTGGT  801
             |||||||||||||||||||||
Sbjct  964   GTTGCTCTCGTAGGAGTTGGT  984
```

>*NtMTP2-transcript variant-X1** - XM_016603324

ATGAATCAGAGTCATACGCCGTCGTTTCATTACAGGAAATTAGAATCCGACTCTCCTAGA
CAACCGTGGAGCGGCGATTTTGATTTCGACGGCAGCGATCGGAGATTCGCATTTTCACGG
CAGAGTTCGTTTCAGCAATCGGCTGAGCCTCACACGCCGATTTCCTTATTAACGAACGAT
TCCGCAAAGCCTTTGCTTACTCGAACTGTTTCGAGCATTGATATACCGCCGAATATTTAT
CCACAACATGCATTTGGCAAGTTGCAGGATTCGGATGTGAAATTTAGGATTTCTACGTAT
GCGTCATCGGTTTTTCGAGGCGTGATGTCTGGGAATAAGCAGATGAGGAGGTTATTTATG
TTGATTTCGCTAAATGTAGCTTATTCTACTGCGGAGTTGTGTATTGGCCTTCTCTCTGGC
CAAGTAGGGTTGGTATCAGATGCATTTCATTTGACCTTTGGTTGTGGCCTTTTGACATTT
TCATTGTTTGCAATGGCCGCTTCTAGAGAAAAGCCCGACCGCGTTTACACTTATGGGTAC
AAAAGGCTCGAGGTCTTGTCTGCTTTCACTAATGCTCAAATAAAAAAGGTTAGATATTTA
GCAAAAATTACAAAAGTCGTTAAGTGCAATTTGGGATGGGAAAGAGGAGTTGCTTGGAGA
AATAGTGTAAGACATGAAATAATCCGTGGCGAAGTCAAAAAATTTACAAAGGGTGTTCAA
GGTTGTTCAGTTGAACACCCTTCACTCAATGTGGCTACGCCATTGGAAATAAGGATGCAG
CTGTTCCTATTGTTCTTGTCATTCTCCTTGGCTGTGGAAGCACTTCATGCATTTATACAA
GATGAATCTGAGCACAAATTTATTGTACCAAGTAATCGACCCTTCTTTGTTTATTACAAA
CCATTTTTTATTAGCCCTAGTCTTCTTATTTTGTTAGCCTCAATCCAGTTGGTTTATCAG
GTTGAAGTCATTCTTAGTTTATTACAGATAATTTATTGTTGTATTAGCTTTAGTCTAGAT
GGTATGGAAGACTTTGTAGTAGTGGGTGGAAATTATTTGGGTGAATGTGTGGAGACTCCA
AATTACTGGATTAGGTGTTCAAAGGTGATGGTACCAATTGCACTGAGTAGGCAAGCTACC
TATGAAGGGTTGGTTGAAAGAGTCATTGAAAGTTGCCATTTGAGAAGCTCGCCGAGCGAT
GTGTCGATTAGTTCTGCGCCTGACATAAAATATTCCAATAACAACAATAACAACATACCC
AGTATTATCCCACACCGTGGGTCTGGGGAGGGTACTAAGTTGCGCGGACTCTTCGTTTTT
GGTGCCGCACGCGTCTCGACACGGGACGGGAGCGGGAGCGAGATACGTCCCAGATTCGGT
CAACTAACTTCGGATACTTTGACCTGA

PREDICTED: Nicotiana tomentosiformis **metal tolerance protein 2** (LOC104099394), transcript variant X1, mRNA
Sequence ID: XM_009606363.1Length: 1976Number of Matches: 1
Range 1: 177 to 977GenBankGraphics Next Match Previous Match First Match

Alignment statistics for match #1

Score	Expect	Identities	Gaps	Strand	Frame
1474 bits(798)	0.0()	800/801(99%)	0/801(0%)	Plus/Plus	

```
Query  1    ATGGTAGCTAGATTCATTTCAAGCAATCTAAATCGAATACGAAAAACGTATATAGCATCA  60
            ||||||||||||||||||||||||||||||||||||||||||||||||||||||||||||
Sbjct  177  ATGGTAGCTAGATTCATTTCAAGCAATCTAAATCGAATACGAAAAACGTATATAGCATCA  236

Query  61   ACTATTAGATACAATAACAATTTCACTCCTCTATATCTATCTCCCAATTTGCAATTCGAT  120
            ||||||||||||||||||||||||||||||||||||||||||||||||||||||||||||
Sbjct  237  ACTATTAGATACAATAACAATTTCACTCCTCTATATCTATCTCCCAATTTGCAATTCGAT  296

Query  121  AATGATGATTCAGCTACAAGTATTGGGAATCGCAGCTTTATGCTTCAGAAAAGATGGCAT  180
            ||||||||||||||||||||||||||||||||||||||||||||||||||||||||||||
Sbjct  297  AATGATGATTCAGCTACAAGTATTGGGAATCGCAGCTTTATGCTTCAGAAAAGATGGCAT  356

Query  181  ATGGGGCATTCACATTCACATCATGACCATGACGACCTCCGCTCCGGTAAAGACGGCGAG  240
            ||||||||||||||||||||||||||||||||||||||||||||||||||||||||||||
Sbjct  357  ATGGGGCATTCACATTCACATCATGACCATGACGACCTCCGCTCCGGTAAAGACGGCGAG  416

Query  241  AGGATTTTCCGGCTTGGCCTCGCTGCTGATATTGGCCTCGCCGCCAGTAAAGCTTTCACA  300
            ||||||||||||||||||||||||||||||||||||||||||||||||||||||||||||
Sbjct  417  AGGATTTTCCGGCTTGGCCTCGCTGCTGATATTGGCCTCGCCGCCAGTAAAGCTTTCACA  476

Query  301  GGTTATATATGCGGTAGCACCGCCATTATCGCTGATGCCGCCCATTCTATCTCCGATGTG  360
            ||||||||||||||||||||||||||||||||||||||||||||||||||||||||||||
Sbjct  477  GGTTATATATGCGGTAGCACCGCCATTATCGCTGATGCCGCCCATTCTATCTCCGATGTG  536

Query  361  GTTCTGAGTGGAGTGGCATTGTTGTCGTTTAAAGCTGCAAGGGTTCCCAAGGACAAAGAA  420
            ||| ||||||||||||||||||||||||||||||||||||||||||||||||||||||||
Sbjct  537  GTTTTGAGTGGAGTGGCATTGTTGTCGTTTAAAGCTGCAAGGGTTCCCAAGGACAAAGAA  596

Query  421  CATCCTTATGGACATGGTAAATTTGAGACTCTTGGAGCTCTTGGAATTTCTGGTGTACTA  480
            ||||||||||||||||||||||||||||||||||||||||||||||||||||||||||||
Sbjct  597  CATCCTTATGGACATGGTAAATTTGAGACTCTTGGAGCTCTTGGAATTTCTGGTGTACTA  656

Query  481  TTGGCTACTGCTGGAGGTATTGGATGGCATGCTTTAGATGTTTTGCTGGGACTATGGTCT  540
            ||||||||||||||||||||||||||||||||||||||||||||||||||||||||||||
Sbjct  657  TTGGCTACTGCTGGAGGTATTGGATGGCATGCTTTAGATGTTTTGCTGGGACTATGGTCT  716

Query  541  ACAGCGCCTGAAGTTGTTAATCAGTCATTGAGTCATTTGGACGTGCATGAGCAACATCAT  600
            ||||||||||||||||||||||||||||||||||||||||||||||||||||||||||||
Sbjct  717  ACAGCGCCTGAAGTTGTTAATCAGTCATTGAGTCATTTGGACGTGCATGAGCAACATCAT  776

Query  601  AGTGGACATCACCATGGAATAGATATGGATCACCCTATTCTTGCTTTGAATGTGACTATA  660
            ||||||||||||||||||||||||||||||||||||||||||||||||||||||||||||
Sbjct  777  AGTGGACATCACCATGGAATAGATATGGATCACCCTATTCTTGCTTTGAATGTGACTATA  836

Query  661  CTCTCTATAGCTGTTAAAGAAGGATTATACTGGATAACAAAGAGAGCGGGGGATAAGATT  720
            ||||||||||||||||||||||||||||||||||||||||||||||||||||||||||||
Sbjct  837  CTCTCTATAGCTGTTAAAGAAGGATTATACTGGATAACAAAGAGAGCGGGGGATAAGATT  896

Query  721  GGCAGTGGACTGATGAAAGCCAATGCTTGGCATCATCGTGCTGATGCAGTATCCTCTGTC  780
            ||||||||||||||||||||||||||||||||||||||||||||||||||||||||||||
Sbjct  897  GGCAGTGGACTGATGAAAGCCAATGCTTGGCATCATCGTGCTGATGCAGTATCCTCTGTC  956

Query  781  GTTGCTCTCGTAGGAGTTGGT  801
            |||||||||||||||||||||
Sbjct  957  GTTGCTCTCGTAGGAGTTGGT  977
```

> *NtMTP2-transcript variant-X2* - XM_016593653

ATGAAGTCTCTATACTTCCTGTCAGATGCTCCGACAGTACTGACTACTGTAGAATATGCG
TCTACAAATTTCTTTGATTTATCTTTCGTAGGAAAACTAGTTCTTTTTCTATTATACTGG
ATGACAAAGAGAGCGGGGGATAAGATTGGCAGTGGACTGATGAAAGCCAATGCTTGGCAT
CATCGTGCTGATGCAGTATCCTCCGTCGTTGCTCTCATAGGAGTTGGTAATGACCTTTAT

ACTCAAATTTTACCTACTTATGGCTGGAGACATAAATCTGTTCTTTTAGAAAACAAGTTT
CACTGTTTGATTTGTCTAATTGTATCATTGAGGATTAAACAAATTTATACTTTCATAGCT
AGTATGCTTGATAGATTTAACTTCATGCTGGTGGTGAAGTTTTCCCTTCGTTCTATACCA
TTAAGCCGAATGACTCAGGAGGTCACTAGTGAAGGAGGACCTAGGGGGTGCAGAGGTGGT
TCGATCCTTGGGGTGAGGATCCTTGATCCACTTGCTGGGCTTGTTGTTGCAGGCATGATC
ATGAAAGCTGGACTTGAAACCGGATATCAGAGATGCAACCGAACATTTAGTCTTTTATCT
GAACCTGCTTGTATAAAATCCTGGGTCCGCCAATGGTGGTTGTTTGTCTTGGAATTGGTT
GATGCTGCTATTCCTTCACATACCCTGAAGCCTTTCAAGCGCACGATTCTACAAGTTGAC
GGAGTTAAGGCAACAATTCCCTTCTCCCACCTACTTGATGTTGATACTGTTGTCAGAAAT
TCCCTTCTCCCACCTACTTGTCAGCACTTAATTATGTATGCTGATGAGATATTTGAGAAA
CATGGGTTGACGGGATGCAGTCACCTGAGGGGAAGGAGGGCTGGTTCATATCTCTATCTT
GATGTTATTGTTGAGGTTGACCCCTTTTCTAGTGTGAGTGCCGCACATGAAATCGGGGAA
AACGTCCGCCGTGAAATCCAGCAGTTACATCCCGAAATTGCTGAAGTTTTCGTACACATA
GAGCCATCCCTTATACACATTCCACCAACTGTTGTGTTTCAGCAGAAGGCTAACACAATG
TGTGGCCCCCAAGACCATTCTGCAATGGAGCTTGCAGACATTGAAAATATAGTTTACAAC
ATATTTTCTACAAACTTTTCTCAGAAGATGATAATTGAGCGTGTAACTCCACATCTATTG
CAAGGACAGATTTTACTTCAAGTTGAAGTTTCTATGCCTCCTGATCTCTTGATTAGGTAC
ACTATTCAAGGTGCTTCACCTGGGGCATCAGATCTTTGTGTGCTTTCTTATAAGTCTCAT
TTATTTTACTTGGGACTGCTTGTTAACTTTCCAGGGATGCGGGAAAAGTTGCAAAAGAAG
CAGAGAAATTAA

PREDICTED: Nicotiana tabacum **metal tolerance protein 2** (LOC107774175), **transcript variant X2**, mRNA
Sequence ID: XM_016593654.1Length: 1815Number of Matches: 5
Range 1: 1088 to 1493GenBankGraphics Next Match Previous Match First Match

Alignment statistics for match #1

Score	Expect	Identities	Gaps	Strand	Frame
664 bits(736)	0.0()	391/406(96%)	0/406(0%)	Plus/Plus	

```
Query  852   GGGATGCAGTCACCTGAGGGGAAGGAGGGCTGGTTCATATCTCTATCTTGATGTTATTGT  911
             ||||||||||||||||||||||||||||||||||||||||||||||||||||||||||||
Sbjct  1088  GGGATGCAGTCACCTGAGGGGAAGGAGGGCTGGTTCATATCTCTATCTTGATGTTATTGT  1147

Query  912   TGAGGTTGACCCCTTTTCTAGTGTGAGTGCCGCACATGAAATCGGGGAAAACGTCCGCCG  971
             ||||||||||||||||||||||||||||||||||||||||||||||||||||||||||||
Sbjct  1148  TGAGGTTGACCCCTTTTCTAGTGTGAGTGCCGCACATGAAATCGGGGAAAACGTCCGCCG  1207

Query  972   TGAAATCCAGCAGTTACATCCCGAAATTGCTGAAGTTTTCGTACACATAGAGCCATCCCT  1031
             |||||||||||||||||||||||||||||| ||||| |||||||||||||||||||||
Sbjct  1208  TGAAATCCAGCAGTTACATCCCGAAATTGCCGAAGTCTTCGTACACATAGAGCCATCCAC  1267

Query  1032  TATACACATTCCACCAACTGTTGTGTTTCAGCAGAAGGCTAACACAATGTGTGGCCCCCA  1091
             ||||||||||||||||||||||||||||||||||| ||||||||||||| ||| ||||||
Sbjct  1268  TATACACATTCCACCAACTGTTGTGTTTCAGCAGAGGGCTAACACAATGGGTGCCCCCCA  1327

Query  1092  AGACCATTCTGCAATGGAGCTTGCAGACATTGAAAATATAGTTTACAACATATTTTCTAC  1151
             |||||||||| |||||||||| |||||||||||||||| |||||||||||| || |||||
Sbjct  1328  AGACCATTCTTCAATGGAGCTCGCAGACATTGAAAATAAAGTTTACAACATTTTATCTAC  1387

Query  1152  AAACTTTTCTCAGAAGATGATAATTGAGCGTGTAACTCCACATCTATTGCAAGGACAGAT  1211
             ||||| ||||||||||||||||||||||||||||| ||||||||| ||||||||||||||
Sbjct  1388  AAACTATTCTCAGAAGATGATAATTGAGCGTGTAAATCCACATCTGTTGCAAGGACAGAT  1447

Query  1212  TTTACTTCAAGTTGAAGTTTCTATGCCTCCTGATCTCTTGATTAGG  1257
             ||||||||||||||||||||||||||||||||||||||||||||||
Sbjct  1448  TTTACTTCAAGTTGAAGTTTCTATGCCTCCTGATCTCTTGATTAGG  1493
```

>*NtMTPC2-like-transcript variant-X1* - XR_001649367

AAGCGATTACCCTGTATAGCTCATTACATGAACTGGATCCATGGTTCAGATGCTATCTTA
TCAATTACTCAATATCTCAAATATCTTATTCTCTGTCCTGGAGTGAAGAATGCTGAAGTC
TTATGTTTGGGACTAGTTTCTGCTACAGTGTTTTTCCTCGTCATGCCACTCTTCAGAGCT
ACTGGTGGCATCTTGCTCCAAATGGCACCCCCAAGGATCCCGTCTTCAGCCTTGAGCAAA
TGCTTGAGACAGGTTTCATCTCGTGAAGATGTTTCAGAAGTTTCTGAAGCTCGTTTTTGG
GAACTGGTGCCTGGTCATGTTATTGGATCAATTTTATTGCAGGTAAAGGAGGGAGTTGAC

GATCGGCCTATTTTGCAATATGTGCATGACTTATACCATGAATTAGGGATACAGGACTTG
ACCTCAGGTGCTCGCCTTTTATCAATCAAGGCAACAATCTTTCAAGGTGTGACATCCCTC
CTCACTCTCCGAAGACTTCCCCTCTTTGAAGTAACTAAAATAAATACGCCTTCAACCCGA
TGGTGTTCTGTTATTTCAATTCCAATAGGTCTAACATTCAGGAACTTTGTGGAGGTTTCA
CTTTCAGGGAGCTTAAAACATAAGCACGCATATGCATCTCAAGTCTCAATGTTGATCGGC
CTATGCAAAAAGTTATCAGGAGCCGTGCAACCTGGAGTTCCAGCAATCTCTTTAGTTGAG
TGGTGA

PREDICTED: Nicotiana tabacum **metal tolerance protein C2-like** (LOC107791892), **transcript variant X1**, misc_RNA
Sequence ID: XR_001649367.1Length: 2195Number of Matches: 3
Range 1: 1120 to 1444GenBankGraphics Next Match Previous Match First Match

Alignment statistics for match #1

Score	Expect	Identities	Gaps	Strand	Frame
587 bits(650)	2e-163()	325/325(100%)	0/325(0%)	Plus/Plus	

```
Query  99    TGGAGTGAAGAATGCTGAAGTCTTATGTTTGGGACTAGTTTCTGCTACAGTGTTTTTCCT  158
             ||||||||||||||||||||||||||||||||||||||||||||||||||||||||||||
Sbjct  1120  TGGAGTGAAGAATGCTGAAGTCTTATGTTTGGGACTAGTTTCTGCTACAGTGTTTTTCCT  1179

Query  159   CGTCATGCCACTCTTCAGAGCTACTGGTGGCATCTTGCTCCAAATGGCACCCCCAAGGAT  218
             ||||||||||||||||||||||||||||||||||||||||||||||||||||||||||||
Sbjct  1180  CGTCATGCCACTCTTCAGAGCTACTGGTGGCATCTTGCTCCAAATGGCACCCCCAAGGAT  1239

Query  219   CCCGTCTTCAGCCTTGAGCAAATGCTTGAGACAGGTTTCATCTCGTGAAGATGTTTCAGA  278
             ||||||||||||||||||||||||||||||||||||||||||||||||||||||||||||
Sbjct  1240  CCCGTCTTCAGCCTTGAGCAAATGCTTGAGACAGGTTTCATCTCGTGAAGATGTTTCAGA  1299

Query  279   AGTTTCTGAAGCTCGTTTTTGGGAACTGGTGCCTGGTCATGTTATTGGATCAATTTTATT  338
             ||||||||||||||||||||||||||||||||||||||||||||||||||||||||||||
Sbjct  1300  AGTTTCTGAAGCTCGTTTTTGGGAACTGGTGCCTGGTCATGTTATTGGATCAATTTTATT  1359

Query  339   GCAGGTAAAGGAGGGAGTTGACGATCGGCCTATTTTGCAATATGTGCATGACTTATACCA  398
             ||||||||||||||||||||||||||||||||||||||||||||||||||||||||||||
Sbjct  1360  GCAGGTAAAGGAGGGAGTTGACGATCGGCCTATTTTGCAATATGTGCATGACTTATACCA  1419

Query  399   TGAATTAGGGATACAGGACTTGACC  423
             |||||||||||||||||||||||||
Sbjct  1420  TGAATTAGGGATACAGGACTTGACC  1444
```

> *NtMTP4-like-transcript variat-X2* - XM_016623041

ATGGATGTGGAAGAGCCATTGCTGAGGAATGAAGTTCCAAATGCGCGTCGAAACTCAGTT
ACTTCTATGAGATGTGATTTCTTCTCTAAACTACCAAGAAAGGTTAAGACAGGGCTTGAT
CCTGAAGAACCTTTTCTTTTGGACTTGTCTAAAACCACTGGTTTAATTAATGGGGAGAAA
GAATATTATGAAAGACAGATTGCAACTTTAAAGTCCTTTGAGGAAGTAGACTCACTTGAT
TCAGCTGATGTCATTGATGAAGAACAAGACCTACTAGATCAAGCACAACATGAAAGAGCA
ATGAACATTTCCAATTTTGCTAATGTCTTATTACTTGCCTTTAAGATCTATGCTACTGTA
AAAAGTGGTTCTTTAGCCATTGCAGCGTCGACGTTGGATTCATTACTTGATCTAATGGCT
GGTGGTATTCTTTGGTTCACTCATCTGTCAATGAAGAGTATAAACATTTACAAGTACCCT
ATAGGAAAGTTAAGAGTTCAACCAGTGGGGATTATCATCTTTGCTGCTGTTATGGCTACT
CTTGGCTTTCAAGTCTTAGTGCAGGCTGTGGAACAACTGATAAAAGATACACCTTCGGAC
AAGATGACTGAGGAGCAACTGGCTTGGCTATATGCAATCATGCTGACAGCCACTGGAGTG
AAGCTCGTCTTATGGATTTATTGCAGAAGTTCAGGAAACAAGATAGTTAGAGCATATGCA
AAGCAACATTTGACAAATTATGGAACCATGCATGATATCCAGGATCATTACTTCGATGTG
GTAACAAATGTTGTCGGTCTGGTTGCTGCTGTTCTTGGTGATCGATTCTATTGGTGGATC
GATCCTGTTGGTGCTATTGTCCTTGCTGTATATACAATTACAAATTGGTCAGGAACTGTG
CTGGAAAATGCAGTTTCTCTAGTTGGACAATCAGCCCCTCCTGAATTTCTACAGAAACTA
ACTTATCTTGTTCTAAGGCATGATCCTCAGATAAAAAGAGTTGATACCGTTCGAGCCTAT
ACTTTTGGAGTTCTTTACTTTGTTGAGGTTGACATAGAGCTCCCAGAAGATTTGCCACTG
AAAGAAGCTCATGCCATTGGAGAGTCACTTCAGATTAAGATTGAAGAACTTCCAGAAGTT
GAACGTGCTTTTGTCCATCTTGATTATGAGTGTGATCACAAACCTGAACACTCCATTTTG
AGTAGAATTCCCAACAGCCCACCTTAA

PREDICTED: Nicotiana tabacum **metal tolerance protein 4-like** (LOC107799900), **transcript variant X2**, mRNA
Sequence ID: XM_016623041.1Length: 1758Number of Matches: 1
Range 1: 387 to 1574GenBankGraphics Next Match Previous Match First Match

Alignment statistics for match #1

Score	Expect	Identities	Gaps	Strand	Frame
2067 bits(2292)	0.0()	1188/1227(97%)	39/1227(3%)	Plus/Plus	

```
Query  1     ATGGATGTGGAAGAGCCATTGCTGAGGAATGAAGTTCCAAATGCGCGTCGAAACTCAGTT  60
             ||||||||||||||||||||||||||||||||||||||||||||||||||||||||||||
Sbjct  387   ATGGATGTGGAAGAGCCATTGCTGAGGAATGAAGTTCCAAATGCGCGTCGAAACTCAGTT  446

Query  61    ACTTCTATGAGATGTGATTTCTTCTCTAAACTACCAAGAAAGGTTAAGACAGGGCTTGAT  120
             ||||||||||||||||||||||||||||||||||||||||||||||||||||||||||||
Sbjct  447   ACTTCTATGAGATGTGATTTCTTCTCTAAACTACCAAGAAAGGTTAAGACAGGGCTTGAT  506

Query  121   CCTGAAGAACCTTTTCTTTTGGACTTGTCTAAAACCACTGGTTTAATTAATGGGGAGAAA  180
             ||||||||||||||||||||||||||||||||||||||||||||||||||||||||||||
Sbjct  507   CCTGAAGAACCTTTTCTTTTGGACTTGTCTAAAACCACTGGTTTAATTAATGGGGAGAAA  566

Query  181   GAATATTATGAAAGACAGATTGCAACTTTAAAGTCCTTTGAGGAAGTAGACTCACTTGAT  240
             ||||||||||||||||||||||||||||||||||||||||||||||||||||||||||||
Sbjct  567   GAATATTATGAAAGACAGATTGCAACTTTAAAGTCCTTTGAGGAAGTAGACTCACTTGAT  626

Query  241   TCAGCTGATGTCATTGATGAAGAACAAGACCTACTAGATCAAGCACAACATGAAAGAGCA  300
             ||||||||||||||||||||||||||||||||||||||||||||||||||||||||||||
Sbjct  627   TCAGCTGATGTCATTGATGAAGAACAAGACCTACTAGATCAAGCACAACATGAAAGAGCA  686

Query  301   ATGAACATTTCCAATTTTGCTAATGTCTTATTACTTGCCTTTAAGATCTATGCTACTGTA  360
             ||||||||||||||||||||||||||||||||||||||||||||||||||||||||||||
Sbjct  687   ATGAACATTTCCAATTTTGCTAATGTCTTATTACTTGCCTTTAAGATCTATGCTACTGTA  746

Query  361   AAAAGTGGTTCTTTAGCCATTGCAGCGTCGACGTTGGATTCATTACTTGATCTAATGGCT  420
             ||||||||||||||||||||||||||||||||||||||||||||||||||||||||||||
Sbjct  747   AAAAGTGGTTCTTTAGCCATTGCAGCGTCGACGTTGGATTCATTACTTGATCTAATGGCT  806

Query  421   GGTGGTATTCTTTGGTTCACTCATCTGTCAATGAAGAGTATAAACATTTACAAGTACCCT  480
             ||||||||||||||||||||||||||||||||||||||||||||||||||||||||||||
Sbjct  807   GGTGGTATTCTTTGGTTCACTCATCTGTCAATGAAGAGTATAAACATTTACAAGTACCCT  866

Query  481   ATAGGAAAGTTAAGAGTTCAACCAGTGGGGATTATCATCTTTGCTGCTGTTATGGCTACT  540
             ||||||||||||||||||||||||||||||||||||||||||||||||||||||||||||
Sbjct  867   ATAGGAAAGTTAAGAGTTCAACCAGTGGGGATTATCATCTTTGCTGCTGTTATGGCTACT  926

Query  541   CTTGGCTTTCAAGTCTTAGTGCAGGCTGTGGAACAACTGATAAAAGATACACCTTCGGAC  600
             ||||||||||||||||||||||||||||||||||||||||||||||||||||||||||||
Sbjct  927   CTTGGCTTTCAAGTCTTAGTGCAGGCTGTGGAACAACTGATAAAAGATACACCTTCGGAC  986

Query  601   AAGATGACTGAGGAGCAACTGGCTTGGCTATATGCAATCATGCTGACAGCCACTGGAGTG  660
             ||||||||||||||||||||||||||||||||||||||||||||||||||||||||||||
Sbjct  987   AAGATGACTGAGGAGCAACTGGCTTGGCTATATGCAATCATGCTGACAGCCACTGGAGTG  1046

Query  661   AAGCTCGTCTTATGGATTTATTGCAGAAGTTCAGGAAACAAGATAGTTAGAGCATATGCA  720
             ||||||||||||||||||||||||||||||||||||||||||||||||||||||||||||
Sbjct  1047  AAGCTCGTCTTATGGATTTATTGCAGAAGTTCAGGAAACAAGATAGTTAGAGCATATGCA  1106

Query  721   AAGCAACATTTGACAAATTATGGAACCATGCATGATATCCAGGATCATTACTTCGATGTG  780
             |                                       ||||||||||||||||||||
Sbjct  1107  A---------------------------------------AGGATCATTACTTCGATGTG  1127

Query  781   GTAACAAATGTTGTCGGTCTGGTTGCTGCTGTTCTTGGTGATCGATTCTATTGGTGGATC  840
             ||||||||||||||||||||||||||||||||||||||||||||||||||||||||||||
Sbjct  1128  GTAACAAATGTTGTCGGTCTGGTTGCTGCTGTTCTTGGTGATCGATTCTATTGGTGGATC  1187

Query  841   GATCCTGTTGGTGCTATTGTCCTTGCTGTATATACAATTACAAATTGGTCAGGAACTGTG  900
             ||||||||||||||||||||||||||||||||||||||||||||||||||||||||||||
Sbjct  1188  GATCCTGTTGGTGCTATTGTCCTTGCTGTATATACAATTACAAATTGGTCAGGAACTGTG  1247

Query  901   CTGGAAAATGCAGTTTCTCTAGTTGGACAATCAGCCCCTCCTGAATTTCTACAGAAACTA  960
             ||||||||||||||||||||||||||||||||||||||||||||||||||||||||||||
Sbjct  1248  CTGGAAAATGCAGTTTCTCTAGTTGGACAATCAGCCCCTCCTGAATTTCTACAGAAACTA  1307
```

```
Query  961   ACTTATCTTGTTCTAAGGCATGATCCTCAGATAAAAAGAGTTGATACCGTTCGAGCCTAT  1020
             ||||||||||||||||||||||||||||||||||||||||||||||||||||||||||||
Sbjct  1308  ACTTATCTTGTTCTAAGGCATGATCCTCAGATAAAAAGAGTTGATACCGTTCGAGCCTAT  1367

Query  1021  ACTTTTGGAGTTCTTTACTTTGTTGAGGTTGACATAGAGCTCCCAGAAGATTTGCCACTG  1080
             ||||||||||||||||||||||||||||||||||||||||||||||||||||||||||||
Sbjct  1368  ACTTTTGGAGTTCTTTACTTTGTTGAGGTTGACATAGAGCTCCCAGAAGATTTGCCACTG  1427

Query  1081  AAAGAAGCTCATGCCATTGGAGAGTCACTTCAGATTAAGATTGAAGAACTTCCAGAAGTT  1140
             ||||||||||||||||||||||||||||||||||||||||||||||||||||||||||||
Sbjct  1428  AAAGAAGCTCATGCCATTGGAGAGTCACTTCAGATTAAGATTGAAGAACTTCCAGAAGTT  1487

Query  1141  GAACGTGCTTTTGTCCATCTTGATTATGAGTGTGATCACAAACCTGAACACTCCATTTTG  1200
             ||||||||||||||||||||||||||||||||||||||||||||||||||||||||||||
Sbjct  1488  GAACGTGCTTTTGTCCATCTTGATTATGAGTGTGATCACAAACCTGAACACTCCATTTTG  1547

Query  1201  AGTAGAATTCCCAACAGCCCACCTTAA  1227
             |||||||||||||||||||||||||||
Sbjct  1548  AGTAGAATTCCCAACAGCCCACCTTAA  1574
```

> *NtMTP4-like* - XM_016626487

ATGGAGGGAGAAGAAGTAAAGAATGAAAACAAGACGCCATTGTTAGAAGGGTGGAAGCTT
AGTGGAAGTGAACGGCGGAGCAGCCACCGGATAAGTCGCCGGAATTCTGTAACATCTCTG
AGGGGTGATTTCTTGGCAAGATTACCTGAAAAGGTCAAGTCTTGTGTTAATGATAATGGT
GATGTTGAATCTTGCTCCAATATTGACCTTTCCAAATCCTCTTGCTTAACTAAAGGAGAA
AAGGACTACTATGAAAAACAATTTGAGACATTGAAGTCATTTGAAGAAGTTGATTCCATT
GTGGCATCTAACTGCATTGATGCTGAGGATCTTGAAGAACAAGCTCAACATGAGAGGGCG
ATGAAGATCTCCAATTACGCAAATGTTTTATTGCTAGCTCTTAAGTTTTTGATTCTCCTT
GACATACGGGAAGTTTTAAATTTAATGGGGCTCAAAAATTGCAATAGGTCACAAGTTCGA
ATCCTAGGTGGCAAAGCAATTTCTGAAGGGGATTCAATTGAACTTAGGTTCGCCAGTACT
TGCACTCCAGAGGAAATTAGTTTGGCAAAGATGGTGATTATGCAATTTCATATAATGGCT
CTGATCTATGCCACGGTGAAGAGTGGTTCCCTGGCTATTGCTGCATCTACATTGGATTCC
TTACTTGATCTCATGGCTGGTGCCATACTGTGGTTGACTCACCTATCAATGAAAAATATC
AACATCTACAAATATCCTATAGGGAAGTTGCGAGTGCAGCCAGTAGGAATTATTATATTT
GCTGCTGTTATGGCTACACTTGGCTTTCAGGTGTTGATTCAGGCTGTAGGACAACTAGTT
GAAAATAAATCTCCCGAAAAGATGACCTCGGATGAACTTTTCTGGTTGTATGCTATCATG
ATAACAGCCACAGTGGTAAAACTTACCCTTTGGCTCTACTGCAGAAGCTCAGGAAACAAC
ATTGTACGTGCCTATGCAAAGGATCACTATTTTGACGTGGTAACTAATGTAGTCGGATTG
GTAGCAGCTGTCCTTGGTGATAAGTTCTACTGGTGGATCGATCCCGTTGGCGCCCTTATC
CTTGCAATTTATACAATCTCCAACTGGTCAGGAACTGTTTTAGAGAACGCAGCGTCATTG
GTGGGACAGTCAGCGCCTCCCGAGTATTTGCAAAAGTTAACATATCTTGTTATAAGACAT
CCCCAAGTGAAGCGCATTGATACAGTTCGGGCATACACATTTGGTGTTTTGTACTTTGTA
GAGGTTGATATTGAACTTCCAGAAGATTTGCCTTTAAAAGAAGCACATACTATTGGAGAA
ACTCTACAAATAAAGCTCGAGAAACTCCCTGACGTTGAACGAGCATTCGTTCATCTCGAT
TTTGAATGCGAACACAAACCAGAGCATTCTGTCCTCAGCAGGCTACCAAACACTGAACCT
TAG

PREDICTED: Nicotiana tabacum **metal tolerance protein 4-like** (LOC107802905), mRNA
Sequence ID: XM_016626487.1Length: 1559Number of Matches: 2
Range 1: 545 to 1385GenBankGraphics Next Match Previous Match First Match

Alignment statistics for match #1

Score	Expect	Identities	Gaps	Strand	Frame
1517 bits(1682)	0.0()	841/841(100%)	0/841(0%)	Plus/Plus	

```
Query  603   GATCTATGCCACGGTGAAGAGTGGTTCCCTGGCTATTGCTGCATCTACATTGGATTCCTT  662
             ||||||||||||||||||||||||||||||||||||||||||||||||||||||||||||
Sbjct  545   GATCTATGCCACGGTGAAGAGTGGTTCCCTGGCTATTGCTGCATCTACATTGGATTCCTT  604

Query  663   ACTTGATCTCATGGCTGGTGCCATACTGTGGTTGACTCACCTATCAATGAAAAATATCAA  722
             ||||||||||||||||||||||||||||||||||||||||||||||||||||||||||||
Sbjct  605   ACTTGATCTCATGGCTGGTGCCATACTGTGGTTGACTCACCTATCAATGAAAAATATCAA  664

Query  723   CATCTACAAATATCCTATAGGGAAGTTGCGAGTGCAGCCAGTAGGAATTATTATATTTGC  782
             ||||||||||||||||||||||||||||||||||||||||||||||||||||||||||||
Sbjct  665   CATCTACAAATATCCTATAGGGAAGTTGCGAGTGCAGCCAGTAGGAATTATTATATTTGC  724
```

```
Query  783   TGCTGTTATGGCTACACTTGGCTTTCAGGTGTTGATTCAGGCTGTAGGACAACTAGTTGA  842
             ||||||||||||||||||||||||||||||||||||||||||||||||||||||||||||
Sbjct  725   TGCTGTTATGGCTACACTTGGCTTTCAGGTGTTGATTCAGGCTGTAGGACAACTAGTTGA  784

Query  843   AAATAAATCTCCCGAAAAGATGACCTCGGATGAACTTTTCTGGTTGTATGCTATCATGAT  902
             ||||||||||||||||||||||||||||||||||||||||||||||||||||||||||||
Sbjct  785   AAATAAATCTCCCGAAAAGATGACCTCGGATGAACTTTTCTGGTTGTATGCTATCATGAT  844

Query  903   AACAGCCACAGTGGTAAAACTTACCCTTTGGCTCTACTGCAGAAGCTCAGGAAACAACAT  962
             ||||||||||||||||||||||||||||||||||||||||||||||||||||||||||||
Sbjct  845   AACAGCCACAGTGGTAAAACTTACCCTTTGGCTCTACTGCAGAAGCTCAGGAAACAACAT  904

Query  963   TGTACGTGCCTATGCAAAGGATCACTATTTTGACGTGGTAACTAATGTAGTCGGATTGGT  1022
             ||||||||||||||||||||||||||||||||||||||||||||||||||||||||||||
Sbjct  905   TGTACGTGCCTATGCAAAGGATCACTATTTTGACGTGGTAACTAATGTAGTCGGATTGGT  964

Query  1023  AGCAGCTGTCCTTGGTGATAAGTTCTACTGGTGGATCGATCCCGTTGGCGCCCTTATCCT  1082
             ||||||||||||||||||||||||||||||||||||||||||||||||||||||||||||
Sbjct  965   AGCAGCTGTCCTTGGTGATAAGTTCTACTGGTGGATCGATCCCGTTGGCGCCCTTATCCT  1024

Query  1083  TGCAATTTATACAATCTCCAACTGGTCAGGAACTGTTTTAGAGAACGCAGCGTCATTGGT  1142
             ||||||||||||||||||||||||||||||||||||||||||||||||||||||||||||
Sbjct  1025  TGCAATTTATACAATCTCCAACTGGTCAGGAACTGTTTTAGAGAACGCAGCGTCATTGGT  1084

Query  1143  GGGACAGTCAGCGCCTCCCGAGTATTTGCAAAAGTTAACATATCTTGTTATAAGACATCC  1202
             ||||||||||||||||||||||||||||||||||||||||||||||||||||||||||||
Sbjct  1085  GGGACAGTCAGCGCCTCCCGAGTATTTGCAAAAGTTAACATATCTTGTTATAAGACATCC  1144

Query  1203  CCAAGTGAAGCGCATTGATACAGTTCGGGCATACACATTTGGTGTTTTGTACTTTGTAGA  1262
             ||||||||||||||||||||||||||||||||||||||||||||||||||||||||||||
Sbjct  1145  CCAAGTGAAGCGCATTGATACAGTTCGGGCATACACATTTGGTGTTTTGTACTTTGTAGA  1204

Query  1263  GGTTGATATTGAACTTCCAGAAGATTTGCCTTTAAAAGAAGCACATACTATTGGAGAAAC  1322
             ||||||||||||||||||||||||||||||||||||||||||||||||||||||||||||
Sbjct  1205  GGTTGATATTGAACTTCCAGAAGATTTGCCTTTAAAAGAAGCACATACTATTGGAGAAAC  1264

Query  1323  TCTACAAATAAAGCTCGAGAAACTCCCTGACGTTGAACGAGCATTCGTTCATCTCGATTT  1382
             ||||||||||||||||||||||||||||||||||||||||||||||||||||||||||||
Sbjct  1265  TCTACAAATAAAGCTCGAGAAACTCCCTGACGTTGAACGAGCATTCGTTCATCTCGATTT  1324

Query  1383  TGAATGCGAACACAAACCAGAGCATTCTGTCCTCAGCAGGCTACCAAACACTGAACCTTA  1442
             ||||||||||||||||||||||||||||||||||||||||||||||||||||||||||||
Sbjct  1325  TGAATGCGAACACAAACCAGAGCATTCTGTCCTCAGCAGGCTACCAAACACTGAACCTTA  1384

Query  1443  G  1443
             |
Sbjct  1385  G  1385
```

> *NtMRP2-like* - XM_009799201

TATAAAAATGCTTTGGGAGGTTCTTGGGTGGTTGTGATACTATTTTTGTGCTATTTCTTG
ATAGAAGCTCTCCGTGTTGGAAGTAGCACATGGTTAAGTTTTTGGACTGATCAAAGCAGT
TCGACGAGATATAGTGCTGGCTTCTACAATCTGATTTATTCACTTTTATCTCTTGGTCAA
GTTATGGTAACTCTGACAAATTCATTTTGGTTGATCACGTCAAGCCTTTATGCTGCCAGG
AGGTTGCATGATGCTATGCTTAATTCTATTTTAAGAGCTCCAATGGTCTTCTTTCACACA
AATCCACTGGGGAGGATTATCAATAGGTTTGCCAAGGATCTAGGGGACATAGATAGAACT
GTTGCACCCTTTGTAAGCATGTTCCTGGGCCAGGTGTTTCAGCTTATCTCAACATTTGTC
CTGATTGGTATAGTGAGTACCATGTCTTTATGGGCAATAATGCCGTTGTTGGTCTTGTTT
TATGGAGCCTATTTATATTATCAGAGCACTGCTCGTGAGGTGAAGCGCTTGGATTCCATC
TCCAGATCTCCTGTCTATGCACAGTTTGGTGAAGCGCTAAATGGTCTTGCAACTATTCGT
GCATATAAAGCTTATGATCGAATGGCCAACATCAATGGGAAGTCAGTGGATAACAATATT
AGGTTTACACTTGTGAACATGGGTGGAAATCGATGGCTTGGGATCCGTCTCGAGACTGTA
GGGGGTCTTATGATTTGGCTTACGGCAACATTTGCTGTGGTGCAGAATGGAAGGGCAGAA
AACCAACAGGCCTTTGCCTCAACAATGGGTTTGCTTCTCAGTTACGCCCTAAATATCACA
AGCTTGTTAACTGCTGTACTGAGACTTGCAAGTTTGGCTGAGAATAGTTTAAATGCTGTC
GAGAGAGTTGGAACGTATATAGACTTACCATCAGAAGGTCCCGCCATAATTGAGGGCAGC
CGTCCTCCTCCTGGATGGCCTTCAGCAGGATCCATTCGATTTGAGAACGTTGTCTTACGT
TACAGGCCCGAACTTCCTCCTGTTCTGCATGGTATATCCTTCACAATTCCTCCGAGTGAC
AAAGTCGGAGTAGTTGGCAGGACTGGAGCTGGAAAATCTAGCATGTTTAATGCTTTATTC
CGACTTGTGGAACCTGAAAGAGGAAGGATATTAATTGATGATTGCGATGTTTCAAAGTTT

GGACTGACAGATCTTCGGAAGGTTCTTGGTATTATACCGCAAGCACCAGTTTTGTTCTCA
GATGTTCGGAGAGTAACATGCCCGAACATTCTGTCTGTTGCCCATCTGACTTTGGAATCT
ATCTTGCTCCACAAAGTCCATGGACATTGTCAAATAATTTTAGTTAGTGGATACGCTTTG
ATGACTTTGAGATGGTCATACAAGAGATGTATAATTTATAAACAAAAAGACCATGGGACC
GTGAGATTTAACCTTGATCCATTCCATGAGCACAATGATGCTGACCTCTGGGAATCTTTG
GAAAGGGCGCACCTTAAGGAAGTTATCAGGAGGAACTCTTTGGGTCTCGACGCCGAGGTG
TCGGAAGCTGGAGAAAATTTCAGTGTTGGACAAAGACAGCTTTTAAGTCTTGCTCGAGCA
TTGCTTCGCCGGTCAAAGATTCTTGTTCTTGATGAAGCAACTGCTGCTGTTGATGTCAGA
ACTGATGCTCTTATTCAAAAAACTATACGTGAAGAATTTAAGTCATGCACCATGCTCATC
ATTGCTCACCGTTTGAACACTATCATCGACTGTGACAGAATTCTTCTGCTTGATGCTGGT
CAGGTCATGCCTGATTGCAGGTTTGAGGGTCTACTTGGGGTTCTAATACAAAAGAGGGAT
TGCGTCTTTATTTTTTCTTTAACCATCTTTTCTCTATGGGTTATTTCAATTTACCTGTTG
TCTGTCTCTTTTCAGGTACTGGAATATGATACTCCAGAAGTATTGTTACAAAAGGAAGAA
ACTGCTTTTTCTAGGATGGTTCAGAGTACAGGAGCTGCAAATGCTCAATACTTACGAAGC
TTAGTATTAGGAGGGGAAGAGGGTAACAGTATAGGGAGGGATAAACAGCTAGATGGGCAA
AGGAGATGGCTTGCCTCTTCACGTTGGACTGCTGCAGCACAGTTTGCACTAGCTGTTACC
CTTACCTCATCCCAAAATGACCTTGTGCAGTTAGAAATTGAGGATGAGGATAATATCCTG
AAGAAAACAAAGAATGCCGTAATCACTCTGCAGGGAGTATTAGAGGGTAAGCACGACAAA
GAAATCGAAGATACCCTGGATCAGTACCAGGTTTCCAGAGACAGATGGTGGTCTTCTTTA
TACAAGATGGTTGAAGGTCTCGCGATGATGAGTAAGCTGGCGCGCAATAGACTTCATCAA
GCAGAACTTGAGGTTGAAGAAAAAGCAATTAACTGGGATCGCGCTGAGATATTTTTTGTC
TACCCCTTAACACTAGTTGTGACATTGATTTTTTATCCCGTGAGAAAGTTTTGCTGTGCC
TCTTCTGAAGATGATGATGAGGTTTCTTGTAAAATTAAGGAGAAATGTTATTTTGAGTGC
AACTACCACAGATACAGGGAACTGGCATTAGAGTGGCTTATTATTATAAGAGATCTCCGA
GTTCTGTGCTGCATGGCTAAAACAACTTATTTTTGGATCCTATCATATATTATTATCCAG
CTTCTATCTACACACACACATCCATGCACCACTGATAGTAGCGTTGGTAATCTCTTTCTA
CAAGCTAGTGATAGTTTTGCGCTAAGGTGGCACGAACAAATCGAGAAATGA

PREDICTED: Nicotiana sylvestris **ABC transporter C family member 2-like** (LOC104243920), partial mRNA
Sequence ID: XM_009799201.1Length: 2432Number of Matches: 4
Range 1: 16 to 1276GenBankGraphics Next Match Previous Match First Match

Alignment statistics for match #1

Score	Expect	Identities	Gaps	Strand	Frame
2324 bits(1258)	0.0()	1260/1261(99%)	0/1261(0%)	Plus/Plus	

```
Query  1    TATAAAAATGCTTTGGGAGGTTCTTGGGTGGTTGTGATACTATTTTTGTGCTATTTCTTG  60
            ||||||||||||||||||||||||||||||||||||||||||||||||||||||||||||
Sbjct  16   TATAAAAATGCTTTGGGAGGTTCTTGGGTGGTTGTGATACTATTTTTGTGCTATTTCTTG  75

Query  61   ATAGAAGCTCTCCGTGTTGGAAGTAGCACATGGTTAAGTTTTTGGACTGATCAAAGCAGT  120
            ||||||||||||||||||||||||||||||||||||||||||||||||||||||||||||
Sbjct  76   ATAGAAGCTCTCCGTGTTGGAAGTAGCACATGGTTAAGTTTTTGGACTGATCAAAGCAGT  135

Query  121  TCGACGAGATATAGTGCTGGCTTCTACAATCTGATTTATTCACTTTTATCTCTTGGTCAA  180
            ||||||||||||||||||||||||||||||||||||||||||||||||||||||||||||
Sbjct  136  TCGACGAGATATAGTGCTGGCTTCTACAATCTGATTTATTCACTTTTATCTCTTGGTCAA  195

Query  181  GTTATGGTAACTCTGACAAATTCATTTTGGTTGATCACGTCAAGCCTTTATGCTGCCAGG  240
            ||||||||||||||||||||||||||||||||||||||||||||||||||||||||||||
Sbjct  196  GTTATGGTAACTCTGACAAATTCATTTTGGTTGATCACGTCAAGCCTTTATGCTGCCAGG  255

Query  241  AGGTTGCATGATGCTATGCTTAATTCTATTTTAAGAGCTCCAATGGTCTTCTTTCACACA  300
            ||||||||||||||||||||||||||||||||||||||||||||||||||||||||||||
Sbjct  256  AGGTTGCATGATGCTATGCTTAATTCTATTTTAAGAGCTCCAATGGTCTTCTTTCACACA  315

Query  301  AATCCACTGGGGAGGATTATCAATAGGTTTGCCAAGGATCTAGGGGACATAGATAGAACT  360
            ||||||||||||||||||||||||||||||||||||||||||||||||||||||||||||
Sbjct  316  AATCCACTGGGGAGGATTATCAATAGGTTTGCCAAGGATCTAGGGGACATAGATAGAACT  375

Query  361  GTTGCACCCTTTGTAAGCATGTTCCTGGGCCAGGTGTTTCAGCTTATCTCAACATTTGTC  420
            ||||||||||||||||||||||||||||||||||||||||||||||||||||||||||||
Sbjct  376  GTTGCACCCTTTGTAAGCATGTTCCTGGGCCAGGTGTTTCAGCTTATCTCAACATTTGTC  435

Query  421  CTGATTGGTATAGTGAGTACCATGTCTTTATGGGCAATAATGCCGTTGTTGGTCTTGTTT  480
```

```
                ||||||||||||||||||||||||||||||||||||||||||||||||||||||||||||
Sbjct  436   CTGATTGGTATAGTGAGTACCATGTCTTTATGGGCAATAATGCCGTTGTTGGTCTTGTTT  495

Query  481   TATGGAGCCTATTTATATTATCAGAGCACTGCTCGTGAGGTGAAGCGCTTGGATTCCATC  540
             ||||||||||||||||||||||||||||||||||||||||||||||||||||||||||||
Sbjct  496   TATGGAGCCTATTTATATTATCAGAGCACTGCTCGTGAGGTGAAGCGCTTGGATTCCATC  555

Query  541   TCCAGATCTCCTGTCTATGCACAGTTTGGTGAAGCGCTAAATGGTCTTGCAACTATTCGT  600
             ||||||||||||||||||||||||||||||||||||||||||||||||||||||||||||
Sbjct  556   TCCAGATCTCCTGTCTATGCACAGTTTGGTGAAGCGCTAAATGGTCTTGCAACTATTCGT  615

Query  601   GCATATAAAGCTTATGATCGAATGGCCAACATCAATGGGAAGTCAGTGGATAACAATATT  660
             ||||||||||||||||||||||||||||||||||||||||||||||||||||||||||||
Sbjct  616   GCATATAAAGCTTATGATCGAATGGCCAACATCAATGGGAAGTCAGTGGATAACAATATT  675

Query  661   AGGTTTACACTTGTGAACATGGGTGGAAATCGATGGCTTGGGATCCGTCTCGAGACTGTA  720
             ||||||||||||||||||||||||||||||||||||||||||||||||||||||||||||
Sbjct  676   AGGTTTACACTTGTGAACATGGGTGGAAATCGATGGCTTGGGATCCGTCTCGAGACTGTA  735

Query  721   GGGGGTCTTATGATTTGGCTTACGGCAACATTTGCTGTGGTGCAGAATGGAAGGGCAGAA  780
             ||||||||||||||||||||||||||||||||||||||||||||||||||||||||||||
Sbjct  736   GGGGGTCTTATGATTTGGCTTACGGCAACATTTGCTGTGGTGCAGAATGGAAGGGCAGAA  795

Query  781   AACCAACAGGCCTTTGCCTCAACAATGGGTTTGCTTCTCAGTTACGCCCTAAATATCACA  840
             ||||| ||||||||||||||||||||||||||||||||||||||||||||||||||||||
Sbjct  796   AACCAGCAGGCCTTTGCCTCAACAATGGGTTTGCTTCTCAGTTACGCCCTAAATATCACA  855

Query  841   AGCTTGTTAACTGCTGTACTGAGACTTGCAAGTTTGGCTGAGAATAGTTTAAATGCTGTC  900
             ||||||||||||||||||||||||||||||||||||||||||||||||||||||||||||
Sbjct  856   AGCTTGTTAACTGCTGTACTGAGACTTGCAAGTTTGGCTGAGAATAGTTTAAATGCTGTC  915

Query  901   GAGAGAGTTGGAACGTATATAGACTTACCATCAGAAGGTCCCGCCATAATTGAGGGCAGC  960
             ||||||||||||||||||||||||||||||||||||||||||||||||||||||||||||
Sbjct  916   GAGAGAGTTGGAACGTATATAGACTTACCATCAGAAGGTCCCGCCATAATTGAGGGCAGC  975

Query  961   CGTCCTCCTCCTGGATGGCCTTCAGCAGGATCCATTCGATTTGAGAACGTTGTCTTACGT  1020
             ||||||||||||||||||||||||||||||||||||||||||||||||||||||||||||
Sbjct  976   CGTCCTCCTCCTGGATGGCCTTCAGCAGGATCCATTCGATTTGAGAACGTTGTCTTACGT  1035

Query  1021  TACAGGCCCGAACTTCCTCCTGTTCTGCATGGTATATCCTTCACAATTCCTCCGAGTGAC  1080
             ||||||||||||||||||||||||||||||||||||||||||||||||||||||||||||
Sbjct  1036  TACAGGCCCGAACTTCCTCCTGTTCTGCATGGTATATCCTTCACAATTCCTCCGAGTGAC  1095

Query  1081  AAAGTCGGAGTAGTTGGCAGGACTGGAGCTGGAAAATCTAGCATGTTTAATGCTTTATTC  1140
             ||||||||||||||||||||||||||||||||||||||||||||||||||||||||||||
Sbjct  1096  AAAGTCGGAGTAGTTGGCAGGACTGGAGCTGGAAAATCTAGCATGTTTAATGCTTTATTC  1155

Query  1141  CGACTTGTGGAACCTGAAAGAGGAAGGATATTAATTGATGATTGCGATGTTTCAAAGTTT  1200
             ||||||||||||||||||||||||||||||||||||||||||||||||||||||||||||
Sbjct  1156  CGACTTGTGGAACCTGAAAGAGGAAGGATATTAATTGATGATTGCGATGTTTCAAAGTTT  1215

Query  1201  GGACTGACAGATCTTCGGAAGGTTCTTGGTATTATACCGCAAGCACCAGTTTTGTTCTCA  1260
             ||||||||||||||||||||||||||||||||||||||||||||||||||||||||||||
Sbjct  1216  GGACTGACAGATCTTCGGAAGGTTCTTGGTATTATACCGCAAGCACCAGTTTTGTTCTCA  1275

Query  1261  G  1261
             |
Sbjct  1276  G  1276
```

> *NtMRP3-like* - XR_001973770

GTTCTTGAAGCGTGCTCCTTAAAGAAAGACCTGGAAATTCTCTCTTTTGGCGATCAAACA
GTAATAGGGGAGAGAGGCATTAATTTGAGCGGTGGACAGAAGCAGAGAATACAGATTGCT
CGTGCTCTTTACCAAGATGCTGATGTTTACCTATTTGATGATCCGTTCAGTGCTGTGGAT
GCTCATACCGGATCCCATCTCTTCAATGAATGTATAATGGGGCTATGGAATTCAAAAACA
GTTTTATATGTTACACATCAAGTGGAGTTTTTGCCTGCTGCGGATTTGATCTTGGTCATG
AAAGATGGAAGGATCAGAGAAGCTGGGAAATACAATGATCTTCTCAAATTAGGTAGTGAC
TTCATGGAACTTGTGGGTGCTCACCAAGAAGCTTTAACAGCAATTGACACAGTTAAGGGA
GAAGCATTGAGAAAGAGTGTGGAAATGACTGGTGATAATACAAATGTGCAGAAGGATAAA
AAGATTCCAGATGGCCAAAATGGTAAAGTGGATGATATTGTTGGAACAAAGGGACAAATT
GTTCAGGAGGAGGAAAGAGAGAAAGGTAGTGTTGGTTTTGCAATTTACTGGAAATATATA
ACAACTGCATATGGAGGTGCTCTCGTGCCATTTATGCTGTTGGCACAAGTTGGTTTTCAG
CTCCTTCAAATTGGAAGCAATTATTGGATGGCTTGGGCAACTCCCGTCTCAAAGAGTGAT

```
CCACCTCCTGTTGGGGGTTCTACTCTCATCATTGTATATGTTGCTTTAGGAATCGCAAGT
GCTTTCTGCATCCTTGCTAGAACCATGCTTCTTGTTACCGCTGGATATAAGACAGCCTCA
TTGCTTTTCCAAAAAATGCATCTTTGCATTTTCCGTGCTCCAATGTCCTTCTTCGATGCC
ACACCAAGTGGGCGGATCCTAAACAGAGCATCGACAGATCAAAGTGCAATTGATCTGAAC
GTTCCCTTTCAAGTTGGATCCTTTGCCTTCACAATAATACAGCTTTTAGGAATTATTGGA
GTAATGTCACAAGTTGCATGGCAGCAATATTACATACCATCAGCACGAGAACTGGCACGA
CTAAATGGGACATGCAAAGCTCCAGTAATACAGCACTTTGCCGAGACAATTTCAGGATCA
AGCACAATTAGAAGTTTCGATCAGGAATCTAGATTCCAGGACACAAGTATGAAATTGATA
GACAATTATTCTCGACCTAAGTTTCACACCGCTGCTGCAATGGAGTGGCTCTGCATGCGT
TTGGATATGCTATCTCTGATCACTTTTGCTTTCTCATTAATTTTCTTGATCTCTCTTCCT
GTTGGAACAATTGACCCAAGTGTTGCTGGCTTAGCTGTTACATATGGGCTTAATCTGAAC
ATATTACAAGCTTGGGTTGTTTGGAATCTTTGTATGATGGAAAATAAAATTATTTCTGTT
GAAAGAATACTTCAGTATACTGCTCTTCCAAGTGAACCTCCTCTTATCATAGAATCTAAC
AGACCAGACCCTAACTGGCCATCTTGTGGAGAGGTTGATTTTAGCAATCTTCAGGTCCGA
TATGCTCCTCACATGCCTCTCGTGTTGCGAGGCCTTACATGCACTTTCTTTGGTGGAAAG
AAGACTGGAATTGTCGGTAGGACAGGCAGCGGTAAATCTACTCTAATACAGACCCTCTTC
CGCATAGTTGAACCAGCTGCTGGACAAATAAAAATAGATGGTATCAGCATCTCCTCAATT
GGTCTACATGATCTACGGTCTAGATTGAGTATAATTCCACAGGATCCAACTATGTTTGAG
GGGACAGTTCGCAGCAACCTAGACCCGCTTGAAGAGTATTCAGATGAACAAATTTGGGAG
GCGCTCGATAAGTGTCAGCTAGGAGAAGAAGTGAGGAAGAAGGAAGGCAAACTTTATTCT
ACAGTATCTGAGAACGGAGAGAACTGGAGTGTAGGCCAAAGGCAGCTGGTCTGTCTTGGC
CGTGTGCTACTGAAAAAGAGCAAGGTCCTGGTCCTTGACGAGGCTACAGCATCTGTCGAC
ACTGCAACTGATAATCTGATTCAGCAAACTCTAAGGCTGCACTTCTCTGATTCCACGGTT
ATAACCATTGCTCATAGGATTACATCTGTGCTTGACAGTGATATGGTCCTACTATTAGAT
CATGGGCTCATTGCTGAATACGACACTCCAGCCAGGTTGTTAGAGAACGAATCCTCATTG
TTTGCTAAGCTCGTGGCAGAGTATAGTATGAGGTCAAATTCAAGTTTTGAGAATGTTTCA
GATACCTTGAACAAGAAGTCAAGAACCAGCGAAATGCAGGTCATGCCTGTGGAAACTGTA
ACAATCCTATGGCAGGGAAAGAAACCTATATCTAGTGATGCAATATTGATTGTGAAATGG
CATTTGTTTTTGTTTAGACTTTTTGATGAGAAAATGTATACGTAA
```

PREDICTED: Nicotiana tomentosiformis **ABC transporter C family member 3-like** (LOC104119292), transcript variant X2, misc_RNA

Sequence ID: XR_001973770.1 Length: 5089Number of Matches: 3

Range 1: 3388 to 4633GenBankGraphics Next Match Previous Match First Match

Alignment statistics for match #1

Score	Expect	Identities	Gaps	Strand	Frame
2279 bits(1234)	0.0()	1243/1247(99%)	1/1247(0%)	Plus/Plus	

```
Query  1039  TGGCAGCAATATTACATACCATCAGCACGAGAACTGGCACGACTAAATGGGACATGCAAA  1098
             ||| ||||||||||||||||||||||||||||||||||||||||||||||||||||||||
Sbjct  3388  TGG-AGCAATATTACATACCATCAGCACGAGAACTGGCACGACTAAATGGGACATGCAAA  3446

Query  1099  GCTCCAGTAATACAGCACTTTGCCGAGACAATTTCAGGATCAAGCACAATTAGAAGTTTC  1158
             ||||||||||||||||||||||||||||||||||||||||||||||||||||||||||||
Sbjct  3447  GCTCCAGTAATACAGCACTTTGCCGAGACAATTTCAGGATCAAGCACAATTAGAAGTTTC  3506

Query  1159  GATCAGGAATCTAGATTCCAGGACACAAGTATGAAATTGATAGACAATTATTCTCGACCT  1218
             |||||||||||||||||||||||| |||||||||||||||||||||||||||||||||||
Sbjct  3507  GATCAGGAATCTAGATTCCAGGACGCAAGTATGAAATTGATAGACAATTATTCTCGACCT  3566

Query  1219  AAGTTTCACACCGCTGCTGCAATGGAGTGGCTCTGCATGCGTTTGGATATGCTATCTCTG  1278
             ||||||||||||||||||||||||||||||||||||||||||||||||||||||||||||
Sbjct  3567  AAGTTTCACACCGCTGCTGCAATGGAGTGGCTCTGCATGCGTTTGGATATGCTATCTCTG  3626

Query  1279  ATCACTTTTGCTTTCTCATTAATTTTCTTGATCTCTCTTCCTGTTGGAACAATTGACCCA  1338
             ||||||||||||||||||||||||||||||||||||||||||||||||||||||||||||
Sbjct  3627  ATCACTTTTGCTTTCTCATTAATTTTCTTGATCTCTCTTCCTGTTGGAACAATTGACCCA  3686

Query  1339  AGTGTTGCTGGCTTAGCTGTTACATATGGGCTTAATCTGAACATATTACAAGCTTGGGTT  1398
             ||||||||||||||||||||||||||||||||||||||||||||||||||||||||||||
Sbjct  3687  AGTGTTGCTGGCTTAGCTGTTACATATGGGCTTAATCTGAACATATTACAAGCTTGGGTT  3746

Query  1399  GTTTGGAATCTTTGTATGATGGAAAATAAAATTATTTCTGTTGAAAGAATACTTCAGTAT  1458
             ||||||||||||||||||||||||||||||||||||||||||||||||||||||||||||
Sbjct  3747  GTTTGGAATCTTTGTATGATGGAAAATAAAATTATTTCTGTTGAAAGAATACTTCAGTAT  3806
```

```
Query  1459  ACTGCTCTTCCAAGTGAACCTCCTCTTATCATAGAATCTAACAGACCAGACCCTAACTGG  1518
             ||||||||||||||||||||||||||||||||||||||||||||||||||||||||||||
Sbjct  3807  ACTGCTCTTCCAAGTGAACCTCCTCTTATCATAGAATCTAACAGACCAGACCCTAACTGG  3866

Query  1519  CCATCTTGTGGAGAGGTTGATTTAGCAATCTTCAGGTCCGATATGCTCCTCACATGCCT  1578
             ||||||||||||||||||||||||||||||||||||||||||||||||||||||||||||
Sbjct  3867  CCATCTTGTGGAGAGGTTGATTTAGCAATCTTCAGGTCCGATATGCTCCTCACATGCCT  3926

Query  1579  CTCGTGTTGCGAGGCCTTACATGCACTTTCTTTGGTGGAAAGAAGACTGGAATTGTCGGT  1638
             || |||||||||||||||||||||||||||||||||||||||||||||||||||||||||
Sbjct  3927  CTAGTGTTGCGAGGCCTTACATGCACTTTCTTTGGTGGAAAGAAGACTGGAATTGTCGGT  3986

Query  1639  AGGACAGGCAGCGGTAAATCTACTCTAATACAGACCCTCTTCCGCATAGTTGAACCAGCT  1698
             ||||||||||||||||||||||||||||||||||||||||||||||||||||||||||||
Sbjct  3987  AGGACAGGCAGCGGTAAATCTACTCTAATACAGACCCTCTTCCGCATAGTTGAACCAGCT  4046

Query  1699  GCTGGACAAATAAAAATAGATGGTATCAGCATCTCCTCAATTGGTCTACATGATCTACGG  1758
             ||||||||||||||||||||||||| ||||||||||||||||||||||||||||||||||
Sbjct  4047  GCTGGACAAATAAAAATAGATGGTACCAGCATCTCCTCAATTGGTCTACATGATCTACGG  4106

Query  1759  TCTAGATTGAGTATAATTCCACAGGATCCAACTATGTTTGAGGGGACAGTTCGCAGCAAC  1818
             ||||||||||||||||||||||||||||||||||||||||||||||||||||||||||||
Sbjct  4107  TCTAGATTGAGTATAATTCCACAGGATCCAACTATGTTTGAGGGGACAGTTCGCAGCAAC  4166

Query  1819  CTAGACCCGCTTGAAGAGTATTCAGATGAACAAATTTGGGAGGCGCTCGATAAGTGTCAG  1878
             ||||||||||||||||||||||||||||||||||||||||||||||||||||||||||||
Sbjct  4167  CTAGACCCGCTTGAAGAGTATTCAGATGAACAAATTTGGGAGGCGCTCGATAAGTGTCAG  4226

Query  1879  CTAGGAGAAGAAGTGAGGAAGAAGGAAGGCAAACTTTATTCTACAGTATCTGAGAACGGA  1938
             ||||||||||||||||||||||||||||||||||||||||||||||||||||||||||||
Sbjct  4227  CTAGGAGAAGAAGTGAGGAAGAAGGAAGGCAAACTTTATTCTACAGTATCTGAGAACGGA  4286

Query  1939  GAGAACTGGAGTGTAGGCCAAAGGCAGCTGGTCTGTCTTGGCCGTGTGCTACTGAAAAAG  1998
             ||||||||||||||||||||||||||||||||||||||||||||||||||||||||||||
Sbjct  4287  GAGAACTGGAGTGTAGGCCAAAGGCAGCTGGTCTGTCTTGGCCGTGTGCTACTGAAAAAG  4346

Query  1999  AGCAAGGTCCTGGTCCTTGACGAGGCTACAGCATCTGTCGACACTGCAACTGATAATCTG  2058
             ||||||||||||||||||||||||||||||||||||||||||||||||||||||||||||
Sbjct  4347  AGCAAGGTCCTGGTCCTTGACGAGGCTACAGCATCTGTCGACACTGCAACTGATAATCTG  4406

Query  2059  ATTCAGCAAACTCTAAGGCTGCACTTCTCTGATTCCACGGTTATAACCATTGCTCATAGG  2118
             ||||||||||||||||||||||||||||||||||||||||||||||||||||||||||||
Sbjct  4407  ATTCAGCAAACTCTAAGGCTGCACTTCTCTGATTCCACGGTTATAACCATTGCTCATAGG  4466

Query  2119  ATTACATCTGTGCTTGACAGTGATATGGTCCTACTATTAGATCATGGGCTCATTGCTGAA  2178
             ||||||||||||||||||||||||||||||||||||||||||||||||||||||||||||
Sbjct  4467  ATTACATCTGTGCTTGACAGTGATATGGTCCTACTATTAGATCATGGGCTCATTGCTGAA  4526

Query  2179  TACGACACTCCAGCCAGGTTGTTAGAGAACGAATCCTCATTGTTTGCTAAGCTCGTGGCA  2238
             ||||||||||||||||||||||||||||||||||||||||||||||||||||||||||||
Sbjct  4527  TACGACACTCCAGCCAGGTTGTTAGAGAACGAATCCTCATTGTTTGCTAAGCTCGTGGCA  4586

Query  2239  GAGTATAGTATGAGGTCAAATTCAAGTTTTGAGAATGTTTCAGATAC  2285
             |||||||||||||||||||||||||||||||||||||||||||||||
Sbjct  4587  GAGTATAGTATGAGGTCAAATTCAAGTTTTGAGAATGTTTCAGATAC  4633
```

> *NtMRP5-like* - XM_016603310

ATGATTAGCTACTTTGTAGACTACCTAGCAGGAGTGGAGACATTCCCTCATGAGGGATAC
ATTTTGGCTGGAATATTCTTCACCGCAAAGTTGGTTGAGACCTTAACGACCCGGCAGTGG
TATCTTGGAGTCGACATTTTGGGCATGCATGTGAGATCAGCTCTCACTGCAATGGTATAT
CGCAAGGGACTGAGGCTCTCGAGTTCAGCTAGGCAAAGTCACACTAGTGGAGAGATTGTT
AATTACATGGCAGTAGATGTTCAGAGAGTAGGTGACTACTCGTGGTATCTTCATGATATA
TGGATGCTGCCTCTTCAAATCATTCTGGCTCTTGCTATTTTGTATAAAAATGTTGGTATT
GCATCTGTGGCAACTCTAGTTGCCACCATTATTTCCATTGTTGCTACTGTCCCATTAGCT
AGGGTTCAGGAAGACTATCAAGATAAACTAATGGGTGCCAAGGACGATAGGATGAGAAAG
ACTTCCGAGTGCCTCAGGAACATGAGGATTCTGAAGTTACAAGCTTGGGAGGATAGGTAT
AGAGTAATGCTAGAGGAAATGCGGAACGTGGAATTCAAGTATCTTCGAAAAGCTCTCTAC
TCTCAGGCTTTCATCACCTTCATTTTCTGGAGCTCCCCTATATTTGTTTCAGCTATTACG
TTTGGTACTTGCATACTGTTGGGCGGTCAGCTTACGGCAGGAAGTGTTCTTTCTGCGTTG
GCAACTTTCAGGATCCTCCAAGAACCACTTAGGAATTTCCCTGACTTGGTGTCGATGATG

```
GCTCAGACAAAAGTATCCCTTGATCGGATTGCTGGATTTCTGCAAGAGGAAGAGTTGCAG
GAAGATGCCACCATTGTGGTGCCTCGAGACATCACAAATGTCGCAATAGAAATTAAAGAT
AGTGAATTTTGGTGGGATCCGTCTTCTTCAAGTCCAACGTTGGCAGGCATACAACTAAGG
GTAGAAAAGGGCATGTGTGTTGCTGTCTGTGGCGTGGTTGGCTCAGGGAAATCAAGCTTC
CTTTCTTGCATCCTTGGTGAGATTCCCAAAATCTCTGGTGAAGTTAGAATATGTGGAAAT
GCTGCTTATGTCTCACAGTCGGCTTGGATACAATCTGGAACTATAGAAGATAATATCCTT
TTTGGTAGCCCAATGGATAAGGCAAAATACAAGGCCGTGATTCATGCTTGTTCTCTGAAA
AAGGACTTAGAACTTTTCTCACATGGAGATCAGACTATTATTGGTGATAGGGGCATAAAT
CTTAGTGGTGGTCAAAAGCAACGTGTGCAACTTGCCAGGGCGCTCTATCAGGACGCTGAT
ATATATTTACTTGATGATCCTTTTAGTGCTGTTGATGCACACACTGGGTCAGAATTATTT
AAGGTCCTGAAGGAAGGTCGTATCAGTCAATGTGGAAAGTATGATGAACTTCTGCAAGCA
GGGACCGACTTCAACGCTTTGGTTTCAGCTCATCATGAAGCAATTGAAGCTATGGATTTT
TCGTACCAATCTTCTGAAGAATTGGAGAAAGATCCTTCGCCTGATGGTTCTGCTGTAGTG
GCTAAAAAATGTGATTCAGGCGAAAAGAGTATTGACAGTCTTGCAAAGGAAGTGCAAGAA
GGTGTCTCAGCTGCTGATAAGAAGGCAATCAAAGAGAAAAAGAAAGCTAAAAGATTGAGA
AAAAAGCAGCTTGTTCAGGAAGAGGAACGAGAGAGGGGGAAAGTTAGCATGAAAGTTTAT
TTGTCATATATGGCGGCCGCTTATAAGGGCTTGTTGATTCCGCTTATCATTCTTGCACAG
ACATTATTTCAGGTGTCGATTGATCAAAGTGTGGTTGATCTTGATATTCCTTTCAGACTT
GGCGGCTTTGCTTCAACTACAATTCAGCTTATTGGTATTGTTGGTGTTATGTCAAAAGTT
ACCTGGCAAGTTTTATTACTTGTCGTCCCAATGGCTATTGCTTGCCTATGGATGCAGAAA
TACTATATGGCTTCATCAAGGGAACTAGTTCGCATTGTTAGCATCCAAAAATCTCCAATC
ATCCATCTTTTTGCTGAGTCAATTGCTGGAGCTGCAACAATCAGAGGTTTTGGGCAAGAA
AAGAGATTTATGAAGAGGAACCTTTATCTCTTGGATTGTTTTGCTCGGCCATTCTTCTGC
AGTCTTGCAGCAATTGAATGGCTTTGCCTACGCATGGAGTTGCTCTCTACATTTGTCTTT
GCTTTCTGCATGGTTTTACTAGTGAGCTTTCCTCATGGAACTATAGATCCTAGTATGGCA
GGCCTTGCCGTGACATATGGGCTAAACTTGAATGCCCGCCTGTCACGATGGATACTCAGC
TTCTGCAAGCTTGAAAACAAGATTATATCAATAGAAAGGATTCATCAATATTGCCATATT
CCTAGTGAGGCCCCTTCAATTATTGAACCTCGCCCCTCATTGTCATGGCCGGAAGAAGGA
ACCATTGAACTGATTGATTTAAAGGTTCGTTATAAGGAGAGTCTTCCAGTTGTGCTTCAT
GGTGTATCTTGCAAATTTCCTGGAGGAAAGAAAATTGGAATTGTGGGGCGCACGGGTAGT
GGTAAATCTACTCTGATTCAGGCCTTATTCAGATTGCTTGAACCAGAAGCTGGAAAAATA
ATAATCGACAACATTGATATTTCAACAATTGGCCTTCATGACCTTCGAAGTCGTTTGAGT
ATTATTCCCCAAGATCCAACATTATTTGAAGGGACAATTCGAGACAACCTTGACCCCCTT
GGAGAACATTCAGATCTAGAAATATGGCAGGCACTTGAGAAGTCCCAGCTTGGAGAGATT
GTCAGGCAAAAAGATCAAAAGCTCGAAACACCAGTCTTGGAGAATGGAGATAATTGGAGT
GTGGGCCAGAGGCAGCTTGTATCTCTTGGGCGGGCTTTACTTAAACAAGCCAGAATTTTG
GTGCTTGATGAAGCGACTGCCTCGGTTGACTCAGCAACAGATAACCTCATCCAGAAGATT
ATTAGGACAGAGTTTAGGGACTGCACTGTTTGTACCATTGCACATCGTATCCCTACAGTT
ATTGACAGTGATCTGGTTCTGGTCCTCAGTGATGGTGCGTTCTCTATGCTTCACTTGTTA
```

PREDICTED: Nicotiana tabacum **ABC transporter C family member 5-like** (LOC107782424), transcript variant X2, mRNA
Sequence ID: XM_016603310.1Length: 5423Number of Matches: 3
Range 1: 1413 to 2796GenBankGraphics Next Match Previous Match First Match

Alignment statistics for match #1

Score	Expect	Identities	Gaps	Strand	Frame
2556 bits(1384)	0.0()	1384/1384(100%)	0/1384(0%)	Plus/Plus	

```
Query  1     ATGATTAGCTACTTTGTAGACTACCTAGCAGGAGTGGAGACATTCCCTCATGAGGGATAC  60
             ||||||||||||||||||||||||||||||||||||||||||||||||||||||||||||
Sbjct  1413  ATGATTAGCTACTTTGTAGACTACCTAGCAGGAGTGGAGACATTCCCTCATGAGGGATAC  1472

Query  61    ATTTTGGCTGGAATATTCTTCACCGCAAAGTTGGTTGAGACCTTAACGACCCGGCAGTGG  120
             ||||||||||||||||||||||||||||||||||||||||||||||||||||||||||||
Sbjct  1473  ATTTTGGCTGGAATATTCTTCACCGCAAAGTTGGTTGAGACCTTAACGACCCGGCAGTGG  1532

Query  121   TATCTTGGAGTCGACATTTTGGGCATGCATGTGAGATCAGCTCTCACTGCAATGGTATAT  180
             ||||||||||||||||||||||||||||||||||||||||||||||||||||||||||||
Sbjct  1533  TATCTTGGAGTCGACATTTTGGGCATGCATGTGAGATCAGCTCTCACTGCAATGGTATAT  1592

Query  181   CGCAAGGGACTGAGGCTCTCGAGTTCAGCTAGGCAAAGTCACACTAGTGGAGAGATTGTT  240
             ||||||||||||||||||||||||||||||||||||||||||||||||||||||||||||
```

```
Sbjct  1593  CGCAAGGGACTGAGGCTCTCGAGTTCAGCTAGGCAAAGTCACACTAGTGGAGAGATTGTT  1652

Query  241   AATTACATGGCAGTAGATGTTCAGAGAGTAGGTGACTACTCGTGGTATCTTCATGATATA  300
             ||||||||||||||||||||||||||||||||||||||||||||||||||||||||||||
Sbjct  1653  AATTACATGGCAGTAGATGTTCAGAGAGTAGGTGACTACTCGTGGTATCTTCATGATATA  1712

Query  301   TGGATGCTGCCTCTTCAAATCATTCTGGCTCTTGCTATTTTGTATAAAAATGTTGGTATT  360
             ||||||||||||||||||||||||||||||||||||||||||||||||||||||||||||
Sbjct  1713  TGGATGCTGCCTCTTCAAATCATTCTGGCTCTTGCTATTTTGTATAAAAATGTTGGTATT  1772

Query  361   GCATCTGTGGCAACTCTAGTTGCCACCATTATTTCCATTGTTGCTACTGTCCCATTAGCT  420
             ||||||||||||||||||||||||||||||||||||||||||||||||||||||||||||
Sbjct  1773  GCATCTGTGGCAACTCTAGTTGCCACCATTATTTCCATTGTTGCTACTGTCCCATTAGCT  1832

Query  421   AGGGTTCAGGAAGACTATCAAGATAAACTAATGGGTGCCAAGGACGATAGGATGAGAAAG  480
             ||||||||||||||||||||||||||||||||||||||||||||||||||||||||||||
Sbjct  1833  AGGGTTCAGGAAGACTATCAAGATAAACTAATGGGTGCCAAGGACGATAGGATGAGAAAG  1892

Query  481   ACTTCCGAGTGCCTCAGGAACATGAGGATTCTGAAGTTACAAGCTTGGGAGGATAGGTAT  540
             ||||||||||||||||||||||||||||||||||||||||||||||||||||||||||||
Sbjct  1893  ACTTCCGAGTGCCTCAGGAACATGAGGATTCTGAAGTTACAAGCTTGGGAGGATAGGTAT  1952

Query  541   AGAGTAATGCTAGAGGAAATGCGGAACGTGGAATTCAAGTATCTTCGAAAAGCTCTCTAC  600
             ||||||||||||||||||||||||||||||||||||||||||||||||||||||||||||
Sbjct  1953  AGAGTAATGCTAGAGGAAATGCGGAACGTGGAATTCAAGTATCTTCGAAAAGCTCTCTAC  2012

Query  601   TCTCAGGCTTTCATCACCTTCATTTTCTGGAGCTCCCCTATATTTGTTTCAGCTATTACG  660
             ||||||||||||||||||||||||||||||||||||||||||||||||||||||||||||
Sbjct  2013  TCTCAGGCTTTCATCACCTTCATTTTCTGGAGCTCCCCTATATTTGTTTCAGCTATTACG  2072

Query  661   TTTGGTACTTGCATACTGTTGGGCGGTCAGCTTACGGCAGGAAGTGTTCTTTCTGCGTTG  720
             ||||||||||||||||||||||||||||||||||||||||||||||||||||||||||||
Sbjct  2073  TTTGGTACTTGCATACTGTTGGGCGGTCAGCTTACGGCAGGAAGTGTTCTTTCTGCGTTG  2132

Query  721   GCAACTTTCAGGATCCTCCAAGAACCACTTAGGAATTTCCCTGACTTGGTGTCGATGATG  780
             ||||||||||||||||||||||||||||||||||||||||||||||||||||||||||||
Sbjct  2133  GCAACTTTCAGGATCCTCCAAGAACCACTTAGGAATTTCCCTGACTTGGTGTCGATGATG  2192

Query  781   GCTCAGACAAAAGTATCCCTTGATCGGATTGCTGGATTTCTGCAAGAGGAAGAGTTGCAG  840
             ||||||||||||||||||||||||||||||||||||||||||||||||||||||||||||
Sbjct  2193  GCTCAGACAAAAGTATCCCTTGATCGGATTGCTGGATTTCTGCAAGAGGAAGAGTTGCAG  2252

Query  841   GAAGATGCCACCATTGTGGTGCCTCGAGACATCACAAATGTCGCAATAGAAATTAAAGAT  900
             ||||||||||||||||||||||||||||||||||||||||||||||||||||||||||||
Sbjct  2253  GAAGATGCCACCATTGTGGTGCCTCGAGACATCACAAATGTCGCAATAGAAATTAAAGAT  2312

Query  901   AGTGAATTTTGGTGGGATCCGTCTTCTTCAAGTCCAACGTTGGCAGGCATACAACTAAGG  960
             ||||||||||||||||||||||||||||||||||||||||||||||||||||||||||||
Sbjct  2313  AGTGAATTTTGGTGGGATCCGTCTTCTTCAAGTCCAACGTTGGCAGGCATACAACTAAGG  2372

Query  961   GTAGAAAAGGGCATGTGTGTTGCTGTCTGTGGCGTGGTTGGCTCAGGGAAATCAAGCTTC  1020
             ||||||||||||||||||||||||||||||||||||||||||||||||||||||||||||
Sbjct  2373  GTAGAAAAGGGCATGTGTGTTGCTGTCTGTGGCGTGGTTGGCTCAGGGAAATCAAGCTTC  2432

Query  1021  CTTTCTTGCATCCTTGGTGAGATTCCCAAAATCTCTGGTGAAGTTAGAATATGTGGAAAT  1080
             ||||||||||||||||||||||||||||||||||||||||||||||||||||||||||||
Sbjct  2433  CTTTCTTGCATCCTTGGTGAGATTCCCAAAATCTCTGGTGAAGTTAGAATATGTGGAAAT  2492

Query  1081  GCTGCTTATGTCTCACAGTCGGCTTGGATACAATCTGGAACTATAGAAGATAATATCCTT  1140
             ||||||||||||||||||||||||||||||||||||||||||||||||||||||||||||
Sbjct  2493  GCTGCTTATGTCTCACAGTCGGCTTGGATACAATCTGGAACTATAGAAGATAATATCCTT  2552

Query  1141  TTTGGTAGCCCAATGGATAAGGCAAAATACAAGGCCGTGATTCATGCTTGTTCTCTGAAA  1200
             ||||||||||||||||||||||||||||||||||||||||||||||||||||||||||||
Sbjct  2553  TTTGGTAGCCCAATGGATAAGGCAAAATACAAGGCCGTGATTCATGCTTGTTCTCTGAAA  2612

Query  1201  AAGGACTTAGAACTTTTCTCACATGGAGATCAGACTATTATTGGTGATAGGGGCATAAAT  1260
             ||||||||||||||||||||||||||||||||||||||||||||||||||||||||||||
Sbjct  2613  AAGGACTTAGAACTTTTCTCACATGGAGATCAGACTATTATTGGTGATAGGGGCATAAAT  2672

Query  1261  CTTAGTGGTGGTCAAAAGCAACGTGTGCAACTTGCCAGGGCGCTCTATCAGGACGCTGAT  1320
             ||||||||||||||||||||||||||||||||||||||||||||||||||||||||||||
Sbjct  2673  CTTAGTGGTGGTCAAAAGCAACGTGTGCAACTTGCCAGGGCGCTCTATCAGGACGCTGAT  2732

Query  1321  ATATATTTACTTGATGATCCTTTTAGTGCTGTTGATGCACACACTGGGTCAGAATTATTT  1380
             ||||||||||||||||||||||||||||||||||||||||||||||||||||||||||||
Sbjct  2733  ATATATTTACTTGATGATCCTTTTAGTGCTGTTGATGCACACACTGGGTCAGAATTATTT  2792
```

```
Query  1381  AAGG  1384
             ||||
Sbjct  2793  AAGG  2796
```

> *NtMRP10-like* - XM_016648918

ATGGCAGATACAAATTTTCCCGAATTGAAGACTGCGTGGCTGCAGCCAATGTCGCGCTGT
TTTTGGGAGGATGCCAGCATCATTGTCTTTCTTGGATTCCTCGGAATCTTACTGCTGGAT
TCACTGTTATGCAAATGCAGAAAGAAGGTTATGACAGTTGATCAGAAGTACACTGTTGGA
ACAGAAGTCCGTGTTTCCTACTCCTACATATTGAGCATCATTTGCACGACTATATTATCG
TGCACTCATCTCATAATGCTCTTGATATTGCAAAAGAGAAATGGTGCTCACTGCCAATTC
AGATTTCCAGTTCTCTCCTCTGAGATTCTGCAATCAACTTCATGGGCAGTCTCATTTTTC
GTGCTCTACAGAACCAGGAGTAGGAAATACATCAAGTTTCCTTGGGTTCTGAGAATCTGG
TGGATTTCCAGCTTCTTTCTGTCTATTGCTCGTGCGACTCTTGATGCCCATTTTGTCATC
ACAAGCGATGAACATCTAGGACTAGCAGACTATGTGGACATCATTGGTCTTATTTCATCT
GCCTGTCTTCTCGGTATCTCAATCCGAGGGAAGACAGGCATAATTCTTGACATCTCGGAC
AGTACAACTGAGCCACTTTTAAATGGGAAGAATGAAAAGGATCCAGAAGACAAAAGGGAC
AGTCCATATGGAAAAGCTAGTCTTCTCCAACTGATCACCTTCTCTTGGCTCAATCCACTA
TTTGAAGTTGGAAACAAGAAGCCCCTTGACCAGGATGAAGTCCCCGATGTTGACTTCAGG
GACTCCGCAAAATTTCTATCTGGTTCCTTTGATGAAAGCCTGAAGTACGTAAAGGGAAGG
AATGGAGCCAAAAACCCATCTATCTATAAGGCCATTTATGTATTTGCAGGGAAGAAAGCA
GCAATCAACGCTGTCTTTGCAGTTATTAGTGCAGGATCATCTTATGTTGGTCCATACCTT
ATGGATGACTTTGTAAATTTCCTCAACGAAAAGAAACTTCGGGGGTTACAAAGTGGCTAT
CTTTTAGCACTAGCTTTTTGTTGTGCAAAAATGGTTGAGACAACAACACAAAGGCAGTGG
ATGTTTGGAGCTCGGCAACTAAGCCTTCGGCTCAGAGCTGCTCTGATATCTCACATTTAC
CAAAAGGGCCTAGCTTTATCAAGTCAATCACACCAAAGCTACACCAGTGGAGAGATAATC
AACTACATGAGCGTAGATGTCGGAAGGATTACGGATTTTATATGGTACCTTAACTCAATG
TGGATGTTACCCATCCAGATATCATTGGCAATCTATGTTTTACACATGAATCTAGGGAAC
GGGGCACTTGTGGCGTTAGGGGCAACCCTGGTAGTGATGACCGCCAACGTGCCCCTGACA
AGGATCCAAAAGGGATATCAAACTAAGATAATGGAATCCAAGGATGAAAGAATGAAAGCT
ACTTCAGAAATTCTGCGAAATATGAAGACTATTAAACTTCAGGCATGGGATAGTTATTAT
CTCCAGAAGCTGGAAATCTTAAGAAAGGTGGAACATAATTGGCTATGGAAATCACTAAGA
TTGTCAGCCCTATCTGATTTTTTCTTCTGGGGATCACCTGCATTTATTTCTGTGGCAACT
TTCTCCGGATGTGTTATGATGGGCATTCCACTGACTGCAGGCCGAATCTTATCTGCATTG
GCCACATTTCGGATGCTTCAAGATCCTATATTCAATTTGCCAGATTTGCTAAACATTATA
GCACGAGGGAAAGTTTCTGCTGATAGAGTTGCTTCCTACCTGCAGGAAGATGAGATTCAA
CCCGATGCAGTTGAATTTGTTCCCAAAGCTGAAACGCAATATGGGGTTGAGATAAAGAGT
GGGAGATTCAGCTGGGACACAGAATCAGGAACTCCCCCAACCCTTGATGGAATAGAATTA
CAAGCTAAGAGGGGAATGAAGGTGGCAATTTGTGGCACTGTTGGATCAGGGAAGTCAAGC
TTGCTCTCTTGTGTACTAGGAGAGATGCCAAAATTGTCAGGGAACGTGAAGATCAGTGGT
GAAGTGGCATATGTTCCTCAGTCTCCTTGGATACTTAGTGGAAATATCAAGGAGAATATT
CTATTTGGAAAACCTTATGAGAGTGTC

PREDICTED: Nicotiana tabacum **putative ABC transporter C family member 10** (LOC107822384), transcript variant X2, mRNA
Sequence ID: XM_016648918.1Length: 5119Number of Matches: 1
Range 1: 289 to 2355GenBankGraphics Next Match Previous Match First Match

Alignment statistics for match #1

Score	Expect	Identities	Gaps	Strand	Frame
3818 bits(2067)	0.0()	2067/2067(100%)	0/2067(0%)	Plus/Plus	

```
Query  1     TTTTGGGAGGATGCCAGCATCATTGTCTTTCTTGGATTCCTCGGAATCTTACTGCTGGAT  60
             ||||||||||||||||||||||||||||||||||||||||||||||||||||||||||||
Sbjct  289   TTTTGGGAGGATGCCAGCATCATTGTCTTTCTTGGATTCCTCGGAATCTTACTGCTGGAT  348

Query  61    TCACTGTTATGCAAATGCAGAAAGAAGGTTATGACAGTTGATCAGAAGTACACTGTTGGA  120
             ||||||||||||||||||||||||||||||||||||||||||||||||||||||||||||
Sbjct  349   TCACTGTTATGCAAATGCAGAAAGAAGGTTATGACAGTTGATCAGAAGTACACTGTTGGA  408

Query  121   ACAGAAGTCCGTGTTTCCTACTCCTACATATTGAGCATCATTTGCACGACTATATTATCG  180
             ||||||||||||||||||||||||||||||||||||||||||||||||||||||||||||
```

```
Sbjct  409   ACAGAAGTCCGTGTTTCCTACTCCTACATATTGAGCATCATTTGCACGACTATATTATCG  468

Query  181   TGCACTCATCTCATAATGCTCTTGATATTGCAAAAGAGAAATGGTGCTCACTGCCAATTC  240
             ||||||||||||||||||||||||||||||||||||||||||||||||||||||||||||
Sbjct  469   TGCACTCATCTCATAATGCTCTTGATATTGCAAAAGAGAAATGGTGCTCACTGCCAATTC  528

Query  241   AGATTTCCAGTTCTCTCCTCTGAGATTCTGCAATCAACTTCATGGGCAGTCTCATTTTTC  300
             ||||||||||||||||||||||||||||||||||||||||||||||||||||||||||||
Sbjct  529   AGATTTCCAGTTCTCTCCTCTGAGATTCTGCAATCAACTTCATGGGCAGTCTCATTTTTC  588

Query  301   GTGCTCTACAGAACCAGGAGTAGGAAATACATCAAGTTTCCTTGGGTTCTGAGAATCTGG  360
             ||||||||||||||||||||||||||||||||||||||||||||||||||||||||||||
Sbjct  589   GTGCTCTACAGAACCAGGAGTAGGAAATACATCAAGTTTCCTTGGGTTCTGAGAATCTGG  648

Query  361   TGGATTTCCAGCTTCTTTCTGTCTATTGCTCGTGCGACTCTTGATGCCCATTTTGTCATC  420
             ||||||||||||||||||||||||||||||||||||||||||||||||||||||||||||
Sbjct  649   TGGATTTCCAGCTTCTTTCTGTCTATTGCTCGTGCGACTCTTGATGCCCATTTTGTCATC  708

Query  421   ACAAGCGATGAACATCTAGGACTAGCAGACTATGTGGACATCATTGGTCTTATTTCATCT  480
             ||||||||||||||||||||||||||||||||||||||||||||||||||||||||||||
Sbjct  709   ACAAGCGATGAACATCTAGGACTAGCAGACTATGTGGACATCATTGGTCTTATTTCATCT  768

Query  481   GCCTGTCTTCTCGGTATCTCAATCCGAGGGAAGACAGGCATAATTCTTGACATCTCGGAC  540
             ||||||||||||||||||||||||||||||||||||||||||||||||||||||||||||
Sbjct  769   GCCTGTCTTCTCGGTATCTCAATCCGAGGGAAGACAGGCATAATTCTTGACATCTCGGAC  828

Query  541   AGTACAACTGAGCCACTTTTAAATGGGAAGAATGAAAAGGATCCAGAAGACAAAAGGGAC  600
             ||||||||||||||||||||||||||||||||||||||||||||||||||||||||||||
Sbjct  829   AGTACAACTGAGCCACTTTTAAATGGGAAGAATGAAAAGGATCCAGAAGACAAAAGGGAC  888

Query  601   AGTCCATATGGAAAAGCTAGTCTTCTCCAACTGATCACCTTCTCTTGGCTCAATCCACTA  660
             ||||||||||||||||||||||||||||||||||||||||||||||||||||||||||||
Sbjct  889   AGTCCATATGGAAAAGCTAGTCTTCTCCAACTGATCACCTTCTCTTGGCTCAATCCACTA  948

Query  661   TTTGAAGTTGGAAACAAGAAGCCCCTTGACCAGGATGAAGTCCCCGATGTTGACTTCAGG  720
             ||||||||||||||||||||||||||||||||||||||||||||||||||||||||||||
Sbjct  949   TTTGAAGTTGGAAACAAGAAGCCCCTTGACCAGGATGAAGTCCCCGATGTTGACTTCAGG  1008

Query  721   GACTCCGCAAAATTTCTATCTGGTTCCTTTGATGAAAGCCTGAAGTACGTAAAGGGAAGG  780
             ||||||||||||||||||||||||||||||||||||||||||||||||||||||||||||
Sbjct  1009  GACTCCGCAAAATTTCTATCTGGTTCCTTTGATGAAAGCCTGAAGTACGTAAAGGGAAGG  1068

Query  781   AATGGAGCCAAAAACCCATCTATCTATAAGGCCATTTATGTATTTGCAGGGAAGAAAGCA  840
             ||||||||||||||||||||||||||||||||||||||||||||||||||||||||||||
Sbjct  1069  AATGGAGCCAAAAACCCATCTATCTATAAGGCCATTTATGTATTTGCAGGGAAGAAAGCA  1128

Query  841   GCAATCAACGCTGTCTTTGCAGTTATTAGTGCAGGATCATCTTATGTTGGTCCATACCTT  900
             ||||||||||||||||||||||||||||||||||||||||||||||||||||||||||||
Sbjct  1129  GCAATCAACGCTGTCTTTGCAGTTATTAGTGCAGGATCATCTTATGTTGGTCCATACCTT  1188

Query  901   ATGGATGACTTTGTAAATTTCCTCAACGAAAAGAAACTTCGGGGGTTACAAAGTGGCTAT  960
             ||||||||||||||||||||||||||||||||||||||||||||||||||||||||||||
Sbjct  1189  ATGGATGACTTTGTAAATTTCCTCAACGAAAAGAAACTTCGGGGGTTACAAAGTGGCTAT  1248

Query  961   CTTTTAGCACTAGCTTTTTGTTGTGCAAAAATGGTTGAGACAACAACACAAAGGCAGTGG  1020
             ||||||||||||||||||||||||||||||||||||||||||||||||||||||||||||
Sbjct  1249  CTTTTAGCACTAGCTTTTTGTTGTGCAAAAATGGTTGAGACAACAACACAAAGGCAGTGG  1308

Query  1021  ATGTTTGGAGCTCGGCAACTAAGCCTTCGGCTCAGAGCTGCTCTGATATCTCACATTTAC  1080
             ||||||||||||||||||||||||||||||||||||||||||||||||||||||||||||
Sbjct  1309  ATGTTTGGAGCTCGGCAACTAAGCCTTCGGCTCAGAGCTGCTCTGATATCTCACATTTAC  1368

Query  1081  CAAAAGGGCCTAGCTTTATCAAGTCAATCACACCAAAGCTACACCAGTGGAGAGATAATC  1140
             ||||||||||||||||||||||||||||||||||||||||||||||||||||||||||||
Sbjct  1369  CAAAAGGGCCTAGCTTTATCAAGTCAATCACACCAAAGCTACACCAGTGGAGAGATAATC  1428

Query  1141  AACTACATGAGCGTAGATGTCGGAAGGATTACGGATTTTATATGGTACCTTAACTCAATG  1200
             ||||||||||||||||||||||||||||||||||||||||||||||||||||||||||||
Sbjct  1429  AACTACATGAGCGTAGATGTCGGAAGGATTACGGATTTTATATGGTACCTTAACTCAATG  1488

Query  1201  TGGATGTTACCCATCCAGATATCATTGGCAATCTATGTTTTACACATGAATCTAGGGAAC  1260
             ||||||||||||||||||||||||||||||||||||||||||||||||||||||||||||
Sbjct  1489  TGGATGTTACCCATCCAGATATCATTGGCAATCTATGTTTTACACATGAATCTAGGGAAC  1548

Query  1261  GGGGCACTTGTGGCGTTAGGGGCAACCCTGGTAGTGATGACCGCCAACGTGCCCCTGACA  1320
             ||||||||||||||||||||||||||||||||||||||||||||||||||||||||||||
Sbjct  1549  GGGGCACTTGTGGCGTTAGGGGCAACCCTGGTAGTGATGACCGCCAACGTGCCCCTGACA  1608
```

```
Query  1321  AGGATCCAAAAGGGATATCAAACTAAGATAATGGAATCCAAGGATGAAAGAATGAAAGCT  1380
             ||||||||||||||||||||||||||||||||||||||||||||||||||||||||||||
Sbjct  1609  AGGATCCAAAAGGGATATCAAACTAAGATAATGGAATCCAAGGATGAAAGAATGAAAGCT  1668

Query  1381  ACTTCAGAAATTCTGCGAAATATGAAGACTATTAAACTTCAGGCATGGGATAGTTATTAT  1440
             ||||||||||||||||||||||||||||||||||||||||||||||||||||||||||||
Sbjct  1669  ACTTCAGAAATTCTGCGAAATATGAAGACTATTAAACTTCAGGCATGGGATAGTTATTAT  1728

Query  1441  CTCCAGAAGCTGGAAATCTTAAGAAAGGTGGAACATAATTGGCTATGGAAATCACTAAGA  1500
             ||||||||||||||||||||||||||||||||||||||||||||||||||||||||||||
Sbjct  1729  CTCCAGAAGCTGGAAATCTTAAGAAAGGTGGAACATAATTGGCTATGGAAATCACTAAGA  1788

Query  1501  TTGTCAGCCCTATCTGATTTTTTCTTCTGGGGATCACCTGCATTTATTTCTGTGGCAACT  1560
             ||||||||||||||||||||||||||||||||||||||||||||||||||||||||||||
Sbjct  1789  TTGTCAGCCCTATCTGATTTTTTCTTCTGGGGATCACCTGCATTTATTTCTGTGGCAACT  1848

Query  1561  TTCTCCGGATGTGTTATGATGGGCATTCCACTGACTGCAGGCCGAATCTTATCTGCATTG  1620
             ||||||||||||||||||||||||||||||||||||||||||||||||||||||||||||
Sbjct  1849  TTCTCCGGATGTGTTATGATGGGCATTCCACTGACTGCAGGCCGAATCTTATCTGCATTG  1908

Query  1621  GCCACATTTCGGATGCTTCAAGATCCTATATTCAATTTGCCAGATTTGCTAAACATTATA  1680
             ||||||||||||||||||||||||||||||||||||||||||||||||||||||||||||
Sbjct  1909  GCCACATTTCGGATGCTTCAAGATCCTATATTCAATTTGCCAGATTTGCTAAACATTATA  1968

Query  1681  GCACGAGGGAAAGTTTCTGCTGATAGAGTTGCTTCCTACCTGCAGGAAGATGAGATTCAA  1740
             ||||||||||||||||||||||||||||||||||||||||||||||||||||||||||||
Sbjct  1969  GCACGAGGGAAAGTTTCTGCTGATAGAGTTGCTTCCTACCTGCAGGAAGATGAGATTCAA  2028

Query  1741  CCCGATGCAGTTGAATTTGTTCCCAAAGCTGAAACGCAATATGGGGTTGAGATAAAGAGT  1800
             ||||||||||||||||||||||||||||||||||||||||||||||||||||||||||||
Sbjct  2029  CCCGATGCAGTTGAATTTGTTCCCAAAGCTGAAACGCAATATGGGGTTGAGATAAAGAGT  2088

Query  1801  GGGAGATTCAGCTGGGACACAGAATCAGGAACTCCCCCAACCCTTGATGGAATAGAATTA  1860
             ||||||||||||||||||||||||||||||||||||||||||||||||||||||||||||
Sbjct  2089  GGGAGATTCAGCTGGGACACAGAATCAGGAACTCCCCCAACCCTTGATGGAATAGAATTA  2148

Query  1861  CAAGCTAAGAGGGGAATGAAGGTGGCAATTTGTGGCACTGTTGGATCAGGGAAGTCAAGC  1920
             ||||||||||||||||||||||||||||||||||||||||||||||||||||||||||||
Sbjct  2149  CAAGCTAAGAGGGGAATGAAGGTGGCAATTTGTGGCACTGTTGGATCAGGGAAGTCAAGC  2208

Query  1921  TTGCTCTCTTGTGTACTAGGAGAGATGCCAAAATTGTCAGGGAACGTGAAGATCAGTGGT  1980
             ||||||||||||||||||||||||||||||||||||||||||||||||||||||||||||
Sbjct  2209  TTGCTCTCTTGTGTACTAGGAGAGATGCCAAAATTGTCAGGGAACGTGAAGATCAGTGGT  2268

Query  1981  GAAGTGGCATATGTTCCTCAGTCTCCTTGGATACTTAGTGGAAATATCAAGGAGAATATT  2040
             ||||||||||||||||||||||||||||||||||||||||||||||||||||||||||||
Sbjct  2269  GAAGTGGCATATGTTCCTCAGTCTCCTTGGATACTTAGTGGAAATATCAAGGAGAATATT  2328

Query  2041  CTATTTGGAAAAACCTTATGAGAGTGTC  2067
             ||||||||||||||||||||||||||||
Sbjct  2329  CTATTTGGAAAAACCTTATGAGAGTGTC  2355
```

> *NtMRP14-like* - XM_016641186

ATGGCAGCTGATTCATGGCTCACATCTCTTGAATGTTCAGCCTCTGAAATCCAATCTTCA
GATAATTCTTCTTTTGTCTCAGTTGCCTTAAAATGGCTTAAGTTCATTTTTCTTATACCA
TGTTCTCAGAGGATTCTGTTATCATCTGTTGATCTGCTTTTCTTGTTGATCTTAATAGTA
TTAGCAGTTAAAAAGTTAAGTTCAAGATTTTTGAAGAATGGAAATTCCACCTCTTCCCTT
AATAAACCTCTCTTAGTAGAAAGGCCTCAAGTTAGAGTTACCTTTTGGTTTTATGCATCT
TTAGCTGTGACAGCTGTTTTAGCCATAGCTTATAGTGTTCTTTGCATATTAGCCTTTACT
CAGGGTGTTCAATCAACTTGGGAAATGACAGAGGCTTTTTTCAGGTTGTTTCAAGCTCTA
ACTTATCTTGTAATTTTTGTGCTAATTGTACACGAGAAGAGATTTGTTGCTGTTTCTCAT
CCCATGCCACTTCGTGTCTATTGGGCGATGAGCTATGTTATCGTGCTTCTTTTCGCGATT
ACTGGCACTATTCGTCTAATTTTGTATGGAAAAAATGTGGATTTGAGCATGAGAATGGAT
GATATAGCTGTCTTGGTTAGTTTCCCTTTGTATTTGTATCTTCTCATTGTTGCCATAAAA
GGATCATCTGGAATTTGTACTAGTAGCCAACATGAAAACTCTAGGCTAGAGACAACAGAT
GAAATGATTCTGATGGATCCTAATGTGAGTGGATATGGCGCCGCTTCACTATTCTCTAAA
GCAGTATGGAATTGGATGAATCCATTGCTTAGTAAAGGATATCAATCCCCTTTAAAGTTA
GATGAAGTGCCTTCTCTCCCACCTGGTTTCCGAGCTGAAAGATTGGCGGATTTCTTCGAA
AAGAATTGGCCTAAGCCAGGTGAAAATGTGAAGTATCCTGTACTAATGACATTAATCAGA
TGTTTCTGGAGAGACATTGTTATAATTAGTGTCCTTGCAATAGTGCAGTTGGCTGTTATG

TATGTTGGACCAGTTCTTATCCAAAGTTTCATTAGTTTTGCTTCTGGGGATAGAACTACA
AATCCCTATGAGGGCTATTACCTAGTCTTGATCCTGTTTGTTTCGAAAGTAATAGAAGTT
CTTAGTGCACATCACTTCAATTTCAAATCTGAGTTACTCGGGATGAAGATTCGGTCATCT
CTTATCACTACTTTATACAAGAAAGGTCTAAGATTGACTTGTTCCTCTAGACAAGCTCAT
GGTGTAGGACAAATAGTGAATTACATGGCCGTTGATTCCCAACAGCTTTCCGATATGATG
CTACAGCTGCATTCGCTTTGGATGATGCCATTACAACTTGCAGCTTCATTACTTCTCTTG
TACTATTACCTGGGTGTTTCTATGTTTGCGGCGTTTGGTTTAATTGTTGGATCTATGATC
TGCACATTGTATATTACACGCAAGAACAATCAATTCCAATTTGAATTGATGATGAAACGC
GATTCAAGGATGAAGGCTATAAATGAAATGTTGGGAAACATGCGCGTCATCAAGTTTCAA
GCATGGGAAGAACACTTCAAAGAAAAGATTCAATCGTTACGTAATGAGGAATTCAGTTGG
CTTAGTAAGTTCACGTACTTGCTTTCTTGCAACTTGTCACTGCTATGGAGTTTGCCACCA
GTCATAGCAGCTCTTACATTTTTAGCTGCAATTCTTTGCAAAATCCCTCTAGATGCTGCC
ACAGTATTCACAGCAACAACAGTTTTCAGAATTTTACAGGATCCAATCAGAACCTTCCCC
CAATCCCTTATGTCAGTTTCACAAGCCATGGTATCGCTAGAAGGTTGTAGTGGAAGGATC
GCGGTTGAGGTAAAAGATGGGAATTTTTCATGGGAAGATGATGGTGATCAAATTGTTCTG
AAAGAAATAAATGTTGAAATCCGAAAGGGGGAACTTGCTGCAATCGTTGGAATGGTTGGA
TCAGGGAAGTCCTCTTTGCTGGCATCAATTCTTGGTGAACTTCATAAGTTATCCGGAGAG
GTCAGAGTGTGTGGAGCCACTGCTTATGTTGCGCAAACCTCATGGATACAGAATGCTACT
ATCCGAGAGAACATCTTGTTTGGTTCACCAATGAACAATGAAAGATATAGAGATGTAGTA
CGGGTTTGTTCCTTGGAGAAAGACTTGGAAATTCTGGAACATGGAGACCAAACTGAGATA
GGAGAACGAGGAATCAACCTCAGCGGAGGTCAGAAGCAGAGGATACAACTTGCAAGAGCA
GTATATCAGGACCGTGATGTCTATCTTCTTGATGATATATTTAGCGCCGTTGATGCTCAA
ACTGGATCAGAAATATTTAAGGTGATGAGAGATGGTAAGATTGTGCAGTCCGGGAAATAT
GAAGAGCTTCTAGAATTGGGAATGGATTTTGGTGATCTTGTCGCTGCACATGAGAACTCA
ATGGAGCTCGTGGAAAGTAGCACTGGTGAGAACCTCCCACAAACACCAAGATCACCTCAT
CAAGTAACCCCAAAGTCGCCGCAGAAATCTCAAGAGGAAACCAATGGTGAAAGCACTTCT
TTAGACCAACAACCAAAGAATAGTTTAAAGCTTATTGAAGAAGAGGAAAGAGAGACTGGT
CATGTCAGTTTTGACGTCTACAAGCAGTACTGCACTGAGGCATTTGGATGGTGGGGAGTA
ATAGTCGTATTAATCATTTCTGCACTATGGCAAGGGTCCACTATGTTGAGTGACTATTGG
CTGGCATATGAAACTTCAGAGGACCATATATTCAGTCCTTCTCTTTTCATAAATGTTTAC
TCAATCATAGCTGCCATTTCTTGCATCTTTGTGATCAGCAGATCATTTCTTGTCGCGTTT
TTGGGTCTCAAAACAGCTCAACATTTCTTTGATCAAATTCTTGACAGCATACTGCATGCT
CCCATGTCATTCTTTGACACTACCCCTTCAGGAAGAATATTAAGTCGAGCATCAACGGAT
CAGGCATATGTTGATTTTATGATTCCATTATTTCTAAGTATAGTGCTTCTGATGTACTTC
ACATTAATTGGCATGTTGTTCATTACATGCCAAAGTGCTTGGCCAACAATTTTCCTCATG
ATTCCCCTAGTTTGGCTTAACATCTGGTACCGGCATATCAACTTTCCTTTTTCATCTGAC
ATACAGAGATACTACATTGCATCTTCGCGCGAACTAACAAGACTTAGTTCAATCACCAAA
GCTCCAATCCTCCATCACTTCTCTGAAACCATATCAGGAATCATGACTGTACGCTGCTTC
AGGAAGGAAGATAACTTCTTTCAAGGAAATGTTGAGAGAGTCAATGCTAATCTGCAGATG
GATTTCCACAGCAACGCGTCAAATGAGTGGTTAGGTCTTCGCCTAGAGTTTATTGGTAGT
ATTTTGATCTGCATTGCCACCATTTTCATGGTCTTGCTCCCAAGCTTTCTTATCTCACCA
GAATATGTCGGCTTGGCACTGTCCTATGGACTGCCTCTAAATGGTGTGCTCTTCTGGACG
GTATACATGAGCTGTATGGTTGAGAACAGAATGGTTTCAGTAGAAAGGATTAAACAGTTC
ATAAGAATACCATCTGAGGCTTCATGGAGGAGACCGAATTGTCTTCCATCGTTAGATTGG
CCCTATCGTGGTGACATTGACATCAACAACTTGAAGGTTCGGTATAGGTCTAATACTCCA
CTTGTTCTGAAAGGAATCTCCCTTAGAATCAACGGAGGAGAAAAAATTGGCATTGTTGGG
CGCACTGGAAGTGGGAAGTCTACTCTGATACAAGTTTTCTTTAGGCTAGTTGAACCTTCA
GCTGGAACCATCATAATTGATGGTGTTGACATTTGCAAGTTAGGACTTCATGATCTTAGA
TCACGCTTCGGCATCATTCCTCAAGAACCAGTCCTATTTCAAGGGACAGTTAGAAGCAAT
ATTGATCCCCTAGGACAGTATTCAGATGATGAAATATGGAAGAGTCTTGAACGCTGCCAA
TTAAAAGATGTGGTAGCAGCCAAACCTGAGAAACTCGATGCGTCAGTGGTAGATAGTGGA
GAGAACTGGAGTGTTGGACAAAGACAGCTCCTTTGCTTAGGGAGAGTCATGCTCAAGAAT
AGTAAAATTCTTTTCATGGATGAAGCAACAGCTTCTGTTGATTCCCAAACTGATGCTGTC
ATCCAAAAGATCATTCGTGAGGACTTTGAAGCCTGCACTATTATCACCATTGCTCACCGA
ATACCAACTGTCATAGACTGTGATCATGTTCTTGTTATAGATGATGGATGGGCAAAGGAA
TATGACAGACCAGCTACCTTACTTGAAAGGCCATCAATATTTGCAGCATTGGTTCAAGAG
TATTCTATCAGATCAACAGAAGCAGAGCCAGGATTTCAAGCTTATGGGTTCGAGAGCCTA
ATCTTTTTAAGTTAA

PREDICTED: Nicotiana tabacum **ABC transporter C family member 14-like** (LOC107815575), transcript variant X3, mRNA

Sequence ID: XM_016641186.1Length: 4838Number of Matches: 3

Range 1: 2637 to 4642GenBankGraphics Next Match Previous Match First Match

Alignment statistics for match #1

Score	Expect	Identities	Gaps	Strand	Frame
3553 bits(3940)	0.0()	2006/2039(98%)	33/2039(1%)	Plus/Plus	

Features:

```
Query  2361  GGTGATGAGAGATGGTAAGATTGTGCAGTCCGGGAAATATGAAGAGCTTCTAGAATTGGG  2420
             ||||||||||||||||||||||||||||||||||||||||||||||||||||||||||||
Sbjct  2637  GGTGATGAGAGATGGTAAGATTGTGCAGTCCGGGAAATATGAAGAGCTTCTAGAATTGGG  2696

Query  2421  AATGGATTTTGGTGATCTTGTCGCTGCACATGAGAACTCAATGGAGCTCGTGGAAAGTAG  2480
             ||||||||||||||||||||||||||||||||||||||||||||||||||||||||||||
Sbjct  2697  AATGGATTTTGGTGATCTTGTCGCTGCACATGAGAACTCAATGGAGCTCGTGGAAAGTAG  2756

Query  2481  CACTGGTGAGAACCTCCCACAAACACCAAGATCACCTCATCAAGTAACCCCAAAGTCGCC  2540
             ||||||||||||||||||||||||||||||||||||||||||||||||||||||||||||
Sbjct  2757  CACTGGTGAGAACCTCCCACAAACACCAAGATCACCTCATCAAGTAACCCCAAAGTCGCC  2816

Query  2541  GCAGAAATCTCAAGAGGAAACCAATGGTGAAAGCACTTCTTTAGACCAACAACCAAAGAA  2600
             ||||||||||||||||||||||||||||||||||||||||||||||||||||||||||||
Sbjct  2817  GCAGAAATCTCAAGAGGAAACCAATGGTGAAAGCACTTCTTTAGACCAACAACCAAAGAA  2876

Query  2601  TAGTTTAAAGCTTATTGAAGAAGAGGAAAGAGAGACTGGTCATGTCAGTTTTGACGTCTA  2660
             ||||||||||||||||||||||||||||||||||||||||||||||||||||||||||||
Sbjct  2877  TAGTTTAAAGCTTATTGAAGAAGAGGAAAGAGAGACTGGTCATGTCAGTTTTGACGTCTA  2936

Query  2661  CAAGCAGTACTGCACTGAGGCATTTGGATGGTGGGGAGTAATAGTCGTATTAATCATTTC  2720
             ||||||||||||||||||||||||||||||||||||||||||||||||||||||||||||
Sbjct  2937  CAAGCAGTACTGCACTGAGGCATTTGGATGGTGGGGAGTAATAGTCGTATTAATCATTTC  2996

Query  2721  TGCACTATGGCAAGGGTCCACTATGTTGAGTGACTATTGGCTGGCATATGAAACTTCAGA  2780
             ||||||||||||||||||||||||||||||||||||||||||||||||||||||||||||
Sbjct  2997  TGCACTATGGCAAGGGTCCACTATGTTGAGTGACTATTGGCTGGCATATGAAACTTCAGA  3056

Query  2781  GGACCATATATTCAGTCCTTCTCTTTTCATAAATGTTTACTCAATCATAGCTGCCATTTC  2840
             ||||||||||||||||||||||||||||||||||||||||||||||||||||||||||||
Sbjct  3057  GGACCATATATTCAGTCCTTCTCTTTTCATAAATGTTTACTCAATCATAGCTGCCATTTC  3116

Query  2841  TTGCATCTTTGTGATCAGCAGATCATTTCTTGTCGCGTTTTTGGGTCTCAAAACAGCTCA  2900
             ||||||||||||||||||||||||||||||||||||||||||||||||||||||||||||
Sbjct  3117  TTGCATCTTTGTGATCAGCAGATCATTTCTTGTCGCGTTTTTGGGTCTCAAAACAGCTCA  3176

Query  2901  ACATTTCTTTGATCAAATTCTTGACAGCATACTGCATGCTCCCATGTCATTCTTTGACAC  2960
             ||||||||||||||||||||||||||||||||||||||||||||||||||||||||||||
Sbjct  3177  ACATTTCTTTGATCAAATTCTTGACAGCATACTGCATGCTCCCATGTCATTCTTTGACAC  3236

Query  2961  TACCCCTTCAGGAAGAATATTAAGTCGAGCATCAACGGATCAGGCATATGTTGATTTTAT  3020
             ||||||||||||||||||||||||||||||||||||||||||||||||||||||||||||
Sbjct  3237  TACCCCTTCAGGAAGAATATTAAGTCGAGCATCAACGGATCAGGCATATGTTGATTTTAT  3296

Query  3021  GATTCCATTATTTCTAAGTATAGTGCTTCTGATGTACTTCACATTAATTGGCATGTTGTT  3080
             ||||||||||||||||||||||||||||||||||||||||||||||||||||||||||||
Sbjct  3297  GATTCCATTATTTCTAAGTATAGTGCTTCTGATGTACTTCACATTAATTGGCATGTTGTT  3356

Query  3081  CATTACATGCCAAAGTGCTTGGCCAACAATTTTCCTCATGATTCCCCTAGTTTGGCTTAA  3140
             ||||||||||||||||||||||||||||||||||||||||||||||||||||||||||||
Sbjct  3357  CATTACATGCCAAAGTGCTTGGCCAACAATTTTCCTCATGATTCCCCTAGTTTGGCTTAA  3416

Query  3141  CATCTGGTACCGGCATATCAACTTTCCTTTTTCATCTGACATACAGAGATACTACATTGC  3200
             |||||||||||||                                 ||||||||||||||
Sbjct  3417  CATCTGGTACCGG---------------------------------AGATACTACATTGC  3443

Query  3201  ATCTTCGCGCGAACTAACAAGACTTAGTTCAATCACCAAAGCTCCAATCCTCCATCACTT  3260
             ||||||||||||||||||||||||||||||||||||||||||||||||||||||||||||
Sbjct  3444  ATCTTCGCGCGAACTAACAAGACTTAGTTCAATCACCAAAGCTCCAATCCTCCATCACTT  3503

Query  3261  CTCTGAAACCATATCAGGAATCATGACTGTACGCTGCTTCAGGAAGGAAGATAACTTCTT  3320
             ||||||||||||||||||||||||||||||||||||||||||||||||||||||||||||
Sbjct  3504  CTCTGAAACCATATCAGGAATCATGACTGTACGCTGCTTCAGGAAGGAAGATAACTTCTT  3563

Query  3321  TCAAGGAAATGTTGAGAGAGTCAATGCTAATCTGCAGATGGATTTCCACAGCAACGCGTC  3380
```

```
                ||||||||||||||||||||||||||||||||||||||||||||||||||||||||||||
Sbjct  3564   TCAAGGAAATGTTGAGAGAGTCAATGCTAATCTGCAGATGGATTTCCACAGCAACGCGTC  3623

Query  3381   AAATGAGTGGTTAGGTCTTCGCCTAGAGTTTATTGGTAGTATTTTGATCTGCATTGCCAC  3440
              ||||||||||||||||||||||||||||||||||||||||||||||||||||||||||||
Sbjct  3624   AAATGAGTGGTTAGGTCTTCGCCTAGAGTTTATTGGTAGTATTTTGATCTGCATTGCCAC  3683

Query  3441   CATTTTCATGGTCTTGCTCCCAAGCTTTCTTATCTCACCAGAATATGTCGGCTTGGCACT  3500
              ||||||||||||||||||||||||||||||||||||||||||||||||||||||||||||
Sbjct  3684   CATTTTCATGGTCTTGCTCCCAAGCTTTCTTATCTCACCAGAATATGTCGGCTTGGCACT  3743

Query  3501   GTCCTATGGACTGCCTCTAAATGGTGTGCTCTTCTGGACGGTATACATGAGCTGTATGGT  3560
              ||||||||||||||||||||||||||||||||||||||||||||||||||||||||||||
Sbjct  3744   GTCCTATGGACTGCCTCTAAATGGTGTGCTCTTCTGGACGGTATACATGAGCTGTATGGT  3803

Query  3561   TGAGAACAGAATGGTTTCAGTAGAAAGGATTAAACAGTTCATAAGAATACCATCTGAGGC  3620
              ||||||||||||||||||||||||||||||||||||||||||||||||||||||||||||
Sbjct  3804   TGAGAACAGAATGGTTTCAGTAGAAAGGATTAAACAGTTCATAAGAATACCATCTGAGGC  3863

Query  3621   TTCATGGAGGAGACCGAATTGTCTTCCATCGTTAGATTGGCCCTATCGTGGTGACATTGA  3680
              ||||||||||||||||||||||||||||||||||||||||||||||||||||||||||||
Sbjct  3864   TTCATGGAGGAGACCGAATTGTCTTCCATCGTTAGATTGGCCCTATCGTGGTGACATTGA  3923

Query  3681   CATCAACAACTTGAAGGTTCGGTATAGGTCTAATACTCCACTTGTTCTGAAAGGAATCTC  3740
              ||||||||||||||||||||||||||||||||||||||||||||||||||||||||||||
Sbjct  3924   CATCAACAACTTGAAGGTTCGGTATAGGTCTAATACTCCACTTGTTCTGAAAGGAATCTC  3983

Query  3741   CCTTAGAATCAACGGAGGAGAAAAAATTGGCATTGTTGGGCGCACTGGAAGTGGGAAGTC  3800
              ||||||||||||||||||||||||||||||||||||||||||||||||||||||||||||
Sbjct  3984   CCTTAGAATCAACGGAGGAGAAAAAATTGGCATTGTTGGGCGCACTGGAAGTGGGAAGTC  4043

Query  3801   TACTCTGATACAAGTTTTCTTTAGGCTAGTTGAACCTTCAGCTGGAACCATCATAATTGA  3860
              ||||||||||||||||||||||||||||||||||||||||||||||||||||||||||||
Sbjct  4044   TACTCTGATACAAGTTTTCTTTAGGCTAGTTGAACCTTCAGCTGGAACCATCATAATTGA  4103

Query  3861   TGGTGTTGACATTTGCAAGTTAGGACTTCATGATCTTAGATCACGCTTCGGCATCATTCC  3920
              ||||||||||||||||||||||||||||||||||||||||||||||||||||||||||||
Sbjct  4104   TGGTGTTGACATTTGCAAGTTAGGACTTCATGATCTTAGATCACGCTTCGGCATCATTCC  4163

Query  3921   TCAAGAACCAGTCCTATTTCAAGGGACAGTTAGAAGCAATATTGATCCCCTAGGACAGTA  3980
              ||||||||||||||||||||||||||||||||||||||||||||||||||||||||||||
Sbjct  4164   TCAAGAACCAGTCCTATTTCAAGGGACAGTTAGAAGCAATATTGATCCCCTAGGACAGTA  4223

Query  3981   TTCAGATGATGAAATATGGAAGAGTCTTGAACGCTGCCAATTAAAAGATGTGGTAGCAGC  4040
              ||||||||||||||||||||||||||||||||||||||||||||||||||||||||||||
Sbjct  4224   TTCAGATGATGAAATATGGAAGAGTCTTGAACGCTGCCAATTAAAAGATGTGGTAGCAGC  4283

Query  4041   CAAACCTGAGAAACTCGATGCGTCAGTGGTAGATAGTGGAGAGAACTGGAGTGTTGGACA  4100
              ||||||||||||||||||||||||||||||||||||||||||||||||||||||||||||
Sbjct  4284   CAAACCTGAGAAACTCGATGCGTCAGTGGTAGATAGTGGAGAGAACTGGAGTGTTGGACA  4343

Query  4101   AAGACAGCTCCTTTGCTTAGGGAGAGTCATGCTCAAGAATAGTAAAATTCTTTTCATGGA  4160
              ||||||||||||||||||||||||||||||||||||||||||||||||||||||||||||
Sbjct  4344   AAGACAGCTCCTTTGCTTAGGGAGAGTCATGCTCAAGAATAGTAAAATTCTTTTCATGGA  4403

Query  4161   TGAAGCAACAGCTTCTGTTGATTCCCAAACTGATGCTGTCATCCAAAAGATCATTCGTGA  4220
              ||||||||||||||||||||||||||||||||||||||||||||||||||||||||||||
Sbjct  4404   TGAAGCAACAGCTTCTGTTGATTCCCAAACTGATGCTGTCATCCAAAAGATCATTCGTGA  4463

Query  4221   GGACTTTGAAGCCTGCACTATTATCACCATTGCTCACCGAATACCAACTGTCATAGACTG  4280
              ||||||||||||||||||||||||||||||||||||||||||||||||||||||||||||
Sbjct  4464   GGACTTTGAAGCCTGCACTATTATCACCATTGCTCACCGAATACCAACTGTCATAGACTG  4523

Query  4281   TGATCATGTTCTTGTTATAGATGATGGATGGGCAAAGGAATATGACAGACCAGCTACCTT  4340
              ||||||||||||||||||||||||||||||||||||||||||||||||||||||||||||
Sbjct  4524   TGATCATGTTCTTGTTATAGATGATGGATGGGCAAAGGAATATGACAGACCAGCTACCTT  4583

Query  4341   ACTTGAAAGGCCATCAATATTTGCAGCATTGGTTCAAGAGTATTCTATCAGATCAACAG  4399
              |||||||||||||||||||||||||||||||||||||||||||||||||||||||||||
Sbjct  4584   ACTTGAAAGGCCATCAATATTTGCAGCATTGGTTCAAGAGTATTCTATCAGATCAACAG  4642
```

> *NtMRP15-like* - XM_016590897

ATGAAGTACTCGAAAGAGAAAGAGGAAGACTACGAGCATTATATCAGCAACAGCAACTGC
CACAGTCACAGCAGCAACAGCAATCATCTTCCAGCCGTCGGCGCAGCACTACTGAATTTT
TCCTGGCGAATGACAAAAAGCATGGTCAAGAAGAGCTCTTGTCGTCCTTGTGCTCTCTCT

CAGAAGCTTTGCCTCCACCTTCTGGCAGCGATTCTAATTTCTATCATACGATCACTGGAT
GTGGTTGCGACTGCGACGACAAGGCGTTGGCGGAACGAGTTGCTGAGTGAAGCCGGCGTA
GAACACTCTTCAATGGTCGACGAAATTTGCCGTCTTTCAAACAAAATTCCTTATAAGTCG
CACGTATTCAACAAATGGGATTGGTCCTACCCTTTCCGCACTTCTTCAAAAGGAGAACAA
CAAAAGTTTGGAATATATAATAACTTATGTGGCCCAATGTCTGAGAGAGACTGGGGGAAG
GAAACTGTGGTGTTAGAATCCATCTCCAAGCTCCCTAATACAGCAAATACAAATTTCCCC
GAATTGAAGAGTGCGTGGCTGCAACCAATGTCGCGCTGCTTGTGGGAGGATGCCAGCATC
ATTGTCTTTCTTGGATTCCTAGGAATCTTACTGCTGGATTCACTACTATGCAAATGCAGA
AAGAAGGTTATGACAGTTGATCAGAAGTACACTGTTGGAACAGAATTCCGTGTTTCCTAC
TCCTACATATTTAGCATTATTTGCACGACTGTGTTATCGAGCACTCATCTCATAATGCTC
TTGATATTGCAAAAGAGAAATGGTGCTCACTGCCAATTCAAACTTCCAGTTCTTTCCTCT
GAGATTCTGCAATCAACATCATGGGCAGTCTCATTTTTCGTGCTCTACAGAAGTCGGAGT
AGGAAAATAAACAACTTTCCTTGGGTCCTTAGAATCTGGTGGATTTCCAGCTTCTTTATA
TATTTTGCTCGTGCGATTTTGGATGCCCATTTTGCCATCACTAGTGATGAACATCTAGGA
CTTGCAGACTATGTGGACATCATTGGTCTTATTGCATCTGCCTGTCTTCTCGGTATCTCA
ATCAGAGGGAAGACAGGCATAATTCTTGACATCTCGGACAGTACAACTGAGCCACTTTTA
AATGGGAAGAATGAAAAGCATCCAGAAGACAAAAGGGACAGTACATATGGAAAAGCTAGT
CTTCTCCAACTGATCACCTTTTCTTGGCTCAATCCACTATTTGAAGTTGGAATCAAGAAA
CCCCTTGATCAGGATGAAATCCCCGATATTGACTTCAGGGACTCTGCAAAATTTCTATCT
GATTCCTTTGATGAAAGCCTGAAGTACGTAAAGGGAAGGAATGGAGCCACAAACCCATCT
ATATATAAGGCCATTTATGTATTTGCAAGGAAGAAAGCAGCAATCAACGCGCTCTTTGCA
GTCATTAGTGCAGGATCATCTTATGTTGGTCCATACCTTATGAATGACTTTGTAATTTTC
CTCAATGAAAAGGAACTTCGGGGGTTACAAAATGGCTATCTTTTAGCACTAGCTTTTTGT
TGTGCAAAAATGGTTGAGACAACAACACAAAGGCAGTGGATGTTTGGAGCTCGGCAACTA
AGCCTTCGGCTCAGAGCTGCTCTGATATCTCACATTTACCAAAAGGGCCTAGCTTTATCA
AGTCAATCACACCAAAGCTACACGAGTGGAGAGATAATCAACTACATGAGCGTAGATGTC
GAAAGGATTACGGATTTTATATGGCACCTTAACTCAATATGGATGTTACCCATCCAGATA
TCATTGGCAATCTATGTTTTACACATGAATCTAGGGAACGGGGCACTTGTGGCGCTAGGG
GCAACCCTGATAGTGATGACCACCAACGTACCCCTGACAAGGATCCAAAAGGGATATCAA
ACTAAGATAATGGAATCCAAGGATGAAAGAATGAAATCTACTTCAGAGATTCTGCGAAAT
ATGAAGACTATTAAACTTCAGGCATGGGATAGTTATTATCTCCATAAGTTGGAAATCTTA
AGAAAGGTGGAACATAATTGGCTGTGGAAATCACTAAGATTGTCAGCCTTATCTGATTTT
TTCTTCTGGGGATCACCTGCATTTATTTCTGTGGCAACCTTTTCCGGATGTGTTATGATG
GGCATACCACTGACTGCAGGCAGGGTCTTATCTACATTGGCCACATTTCGGATGCTTCAA
GATCCTATATTCAATTTGCCAGATTTGCTAAATGTCATAGCACGAGGAAAAGTTTCTGCT
GATAGAGTTGCTTCCTACCTGCAGGAAGATGAGATTCAACCCGATGCAGTTGAATTTGTT
CCTAAAGCTGAAACGCAATATGGGGTTGAGATAAAGAGTGGGAAATTCAGCTGGGACACA
GAATCAGGAACTCCCCCAACCCTTGATGGAATAGAATTACAAGTTAAGAGGGGAATGAAG
GTGGCAATTTGTGGCACTGTTGGATCAGGAAAGTCAAGCTTGCTCTCTTGTGTACTAGGA
GAGATGCCAAAATTGTCGGGGAATGTGAAGATCAGCGGTGAAGTGGCATATGTTCCTCAG
TCTCCTTGGATACTTACTGGAAATATCAAGGAGAATATTCTATTTGGAAAACCTTATGAG
AGTGTTAAGTATGACAGAACAGTTGAAGCATGCGCGCTGAAAAAAGATTTTGAACTATTC
CCTGCCGGTGATCTTACAGAAATTGGAGAAAGAGGGATAAATATGAGCGGAGGTCAGAAG
CAAAGAATACAAATCGCTCGTGCAGCTTACCAAGATGCTGATATATATCTGCTCGATGAC
CCTTTCAGTGCCGTTGATGCTCACACAGGCACGCAGCTCTTTCAGGAGTGCTTGAGGGGG
GTTCTCAAGGACAAGACCATACTTTATGTTACACACCAAGTTGAGTTTCTTCCTGCAGCA
GATCTCATTCTGGTGATGCAAAATGGAAGAATTGCACAAGCTGGAACTTTTGAAGAACTA
CTGAAACAAAATATTGGATTTGAAGTTCTAGTTGGAGCACACAACCAGGCTTTAGAATCA
ATATTAACAGTTGAAAGCTCAAGTAGAATATCTGAAAAAGCAATTACTGGTAGTGAGATG
GATACAGAGAGTAACATAATTACAGAAACTAAGCAGGATTCAGAACACAGTCTCTGTGTA
GAGATACCAGAAAAGGATGGAAGACTTGTGCAGGATGAGGAGAGAGTCAAAGGAAGCATT
GGAAAGGAAGTTTACTATTCTTACTTGACCTCTGTGAAAGGTGGTGCCTTTGTCCCAATA
ATTCTTATAGCGCAATCATCATTTCAAGTGCTTCAGATAGCCAGCAACTACTGGATGGCA
TCGGCATGTCCCACAGGTGATGATGTAGCACCAATAGCTGAGAAGATGAACTTCATACTT
TTTGTTTATGTGCTTCTCGCTGTTGGAAGTTCCCTTTGCGTGCTAGTGCGGGCATCATTT
GTGGCTATAACAGGCCTTCAAACAGCAGAAAAGCTCTTTAGCAACATGCTGCACAGCATC
TTTCACGCTCCTATGTCATTCTTTGACTCTACTCCTACTGGAAGAATCTTAAACCGCGCA
TCCACCGACCAAAGTGTTGTGGACTTGGAAATTGCACTCAAGTTGGGTTGGTGTGCCCTC
TCCATTATTCAGCTTCTTGGGACAATTGCTGTCATGTCGCAGGTCGCATGGGAAGTATTT
GTCCTCTTTATTCCAATAACAGCAGTTTATGTCTGGTACCAGCAATACTACATACCAACC
GCAAGGGAACTTGCTCGTTTATCTGGAGTTCAAAGAGCTCCAATCCTCCATCACTTTGCA

```
GAATCACTGTCAGGAGCAGCAACAATTCGTGCTTTCAACCAAAAAGATCGCTTTGCTCAT
GCAAACCTTAGTCTCATAGATGGTCATTCAAGGCCATGGTTCCACAATATATCAGCACAA
GAATGGCTATCTTTTAGACTGAATCAGCTTTCTACTTTTGTTTTTGCCTTCTTCCTTGTT
CTGCTAGTCACACTCCCCGAAGGAATTATAAATCCAAGCATTGCAGGATTAGCAGTAACA
TACGGCATCTATTTGAACTATTCACAAGCTGCAGTAATATGGAATATTTGCGGTACTGAA
AACAAAATGATATCAGTTGAAAGGATTCTCCAGTATTCAGACCTCGCCAGTGAAGCACCC
CTCGTGATTGAAAATTGCAGACTATCAAGCACCTGGCCAGAAACTGGAACAATTTCCTTC
CAAAATTTACAGATACGATATGCTGAACACCTCCCGTCTGTTTTGAAAAACATCACATGC
ACATTTCCAGGAAGTAAGAAAATTGGTGTTGTGGGCAGGACAGGAAGTGGTAAATCAACC
CTCACTCAAGCCCTTTTCCGGATCGTAGAACCCAGAGAAGGAAGCATTATCATTGACAAT
ATAGATATTTGCAAGATAGGTCTTCATGATTTGAGGTCAAGGCTTAGTATTATTCCTCAA
GATCCAACAATGTTCGACGGAACAGTTAGAGGAAACCTAGATCCACTAGCACAGCACTCT
GATACTGAAATCTGGGAGCTATGGCGTATTTCCTTATTGAAAGCTACTAATGTGGTCAGT
TTCACAGTGGCTGAAAACGGAGAAAACTGGAGTGTAGGTCAAAGGCAACTTTTCTGTCTC
GGACGAGCCTTGCTAAAGAAAAGCAGCATTCTCATTCTAGATGAAGCAACAGCATCAGTT
GATGCTGCAACTGATGCAGTGTTACAAAAGATCATCAGTCAAGAGTTCAGAAATCGAACA
GTTATCACAATAGCACACAGGATCCATACAGTCATTAATAGCGATCTTGTCTTAGTCTTG
AATGAAGGAAGAATAGCTGAATATGATTCACCAGCAAAGCTATTGGAAAGAGAGGATTCT
TTCTTCTCAAAACTGATAAAGGAGTATTTTATGAGATCCAAAAGTACGGCTTAGTAA
```

PREDICTED: Nicotiana tabacum **putative ABC transporter C family member 15** (LOC107771516), transcript variant X2, mRNA
Sequence ID: XM_016590897.1Length: 4994Number of Matches: 1
Range 1: 86 to 4611GenBankGraphics Next Match Previous Match First Match

Alignment statistics for match #1

Score	Expect	Identities	Gaps	Strand	Frame
7990 bits(8860)	0.0()	4490/4528(99%)	19/4528(0%)	Plus/Plus	

```
Query  464   AGAGAGACTGGGGGAAGGAAACTGTGGTGTTAGAATCCATCTCCAAGCTCCCTAATACAG  523
             ||||||||||||||||||||||||||||||||||||||||||||||||||||||||||||
Sbjct  86    AGAGAGACTGGGGGAAGGAAACTGTGGTGTTAGAATCCATCTCCAAGCTCCCTAATACAG  145

Query  524   CAAATACAAATTTCCCCGAATTGAAGAGTGCGTGGCTGCAACCAATGTCGCGCTGCTTGT  583
             ||||||||||||||||||||||||||||||||||||||||||||||||||||||||||||
Sbjct  146   CAAATACAAATTTCCCCGAATTGAAGAGTGCGTGGCTGCAACCAATGTCGCGCTGCTTGT  205

Query  584   GGGAGGATGCCAGCATCATTGTCTTTCTTGGATTCCTAGGAATCTTACTGCTGGATTCAC  643
             ||||||||||||||||||||||||||||||||||||||||||||||||||||||||||||
Sbjct  206   GGGAGGATGCCAGCATCATTGTCTTTCTTGGATTCCTAGGAATCTTACTGCTGGATTCAC  265

Query  644   TACTATGCAAATGCAGAAAGAAGGTTATGACAGTTGATCAGAAGTACACTGTTGGAACAG  703
             ||||||||||||||||||||||||||||||||||||||||||||||||||||||||||||
Sbjct  266   TACTATGCAAATGCAGAAAGAAGGTTATGACAGTTGATCAGAAGTACACTGTTGGAACAG  325

Query  704   AATTCCGTGTTTCCTACTCCTACATATTTAGCATTATTTGCACGACTGTGTTATCGAGCA  763
             ||||||||||||||||||||||||||||||||||||||||||||||||||||||||||||
Sbjct  326   AATTCCGTGTTTCCTACTCCTACATATTTAGCATTATTTGCACGACTGTGTTATCGAGCA  385

Query  764   CTCATCTCATAATGCTCTTGATATTGCAAAAGAGAAATGGTGCTCACTGCCAATTCAAAC  823
             ||||||||||||||||||||||||||||||||||||||||||||||||||||||||||||
Sbjct  386   CTCATCTCATAATGCTCTTGATATTGCAAAAGAGAAATGGTGCTCACTGCCAATTCAAAC  445

Query  824   TTCCAGTTCTTTCCTCTGAGATTCTGCAATCAACATCATGGGCAGTCTCATTTTTCGTGC  883
             ||||||||||||||||||||||||||||||||||||||||||||||||||||||||||||
Sbjct  446   TTCCAGTTCTTTCCTCTGAGATTCTGCAATCAACATCATGGGCAGTCTCATTTTTCGTGC  505

Query  884   TCTACAGAAGTCGGAGTAGGAAAATAAACAACTTTCCTTGGGTCCTTAGAATCTGGTGGA  943
             ||||||||||||||||||||||||||||||||||||||||||||||||||||||||||||
Sbjct  506   TCTACAGAAGTCGGAGTAGGAAAATAAACAACTTTCCTTGGGTCCTTAGAATCTGGTGGA  565

Query  944   TTTCCAGCTTCTTTATATATTTTGCTCGTGCGATTTTGGATGCCCATTTTGCCATCACTA  1003
             ||||||||||||||||||||||||||||||||||||||||||||||||||||||||||||
Sbjct  566   TTTCCAGCTTCTTTATATATTTTGCTCGTGCGATTTTGGATGCCCATTTTGCCATCACTA  625

Query  1004  GTGATGAACATCTAGGACTTGCAGACTATGTGGACATCATTGGTCTTATTGCATCTGCCT  1063
             ||||||||||||||||||||||||||||||||||||||||||||||||||||||||||||
Sbjct  626   GTGATGAACATCTAGGACTTGCAGACTATGTGGACATCATTGGTCTTATTGCATCTGCCT  685
```

```
Query  1064  GTCTTCTCGGTATCTCAATCAGAGGGAAGACAGGCATAATTCTTGACATCTCGGACAGTA  1123
             ||||||||||||||||||||||||||||||||||||||||||||||||||||||||||||
Sbjct  686   GTCTTCTCGGTATCTCAATCAGAGGGAAGACAGGCATAATTCTTGACATCTCGGACAGTA  745

Query  1124  CAACTGAGCCACTTTTAAATGGGAAGAATGAAAAGCATCCAGAAGACAAAAGGGACAGTA  1183
             ||||||||||||||||||||||||||||||||||||||||||||||||||||||||||||
Sbjct  746   CAACTGAGCCACTTTTAAATGGGAAGAATGAAAAGCATCCAGAAGACAAAAGGGACAGTA  805

Query  1184  CATATGGAAAAGCTAGTCTTCTCCAACTGATCACCTTTTCTTGGCTCAATCCACTATTTG  1243
             ||||||||||||||||||||||||||||||||||||||||||||||||||||||||||||
Sbjct  806   CATATGGAAAAGCTAGTCTTCTCCAACTGATCACCTTTTCTTGGCTCAATCCACTATTTG  865

Query  1244  AAGTTGGAATCAAGAAACCCCTTGATCAGGATGAAATCCCCGATATTGACTTCAGGGACT  1303
             ||||||||||||||||||||||||||||||||||||||||||||||||||||||||||||
Sbjct  866   AAGTTGGAATCAAGAAACCCCTTGATCAGGATGAAATCCCCGATATTGACTTCAGGGACT  925

Query  1304  CTGCAAAATTTCTATCTGATTCCTTTGATGAAAGCCTGAAGTACGTAAAGGGAAGGAATG  1363
             ||||||||||||||||||||||||||||||||||||||||||||||||||||||||||||
Sbjct  926   CTGCAAAATTTCTATCTGATTCCTTTGATGAAAGCCTGAAGTACGTAAAGGGAAGGAATG  985

Query  1364  GAGCCACAAACCCATCTATATATAAGGCCATTTATGTATTTGCAAGGAAGAAAGCAGCAA  1423
             ||||||||||||||||||||||||||||||||||||||||||||||||||||||||||||
Sbjct  986   GAGCCACAAACCCATCTATATATAAGGCCATTTATGTATTTGCAAGGAAGAAAGCAGCAA  1045

Query  1424  TCAACGCGCTCTTTGCAGTCATTAGTGCAGGATCATCTTATGTTGGTCCATACCTTATGA  1483
             ||||||||||||||||||||||||||||||||||||||||||||||||||||||||||||
Sbjct  1046  TCAACGCGCTCTTTGCAGTCATTAGTGCAGGATCATCTTATGTTGGTCCATACCTTATGA  1105

Query  1484  ATGACTTTGTAATTTTCCTCAATGAAAAGGAACTTCGGGGGTTACAAAATGGCTATCTTT  1543
             ||||||||||||||||||||||||||||||||||||||||||||||||||||||||||||
Sbjct  1106  ATGACTTTGTAATTTTCCTCAATGAAAAGGAACTTCGGGGGTTACAAAATGGCTATCTTT  1165

Query  1544  TAGCACTAGCTTTTTGTTGTGCAAAAATGGTTGAGACAACAACACAAAGGCAGTGGATGT  1603
             ||||||||||||||||||||||||||||||||||||||||||||||||||||||||||||
Sbjct  1166  TAGCACTAGCTTTTTGTTGTGCAAAAATGGTTGAGACAACAACACAAAGGCAGTGGATGT  1225

Query  1604  TTGGAGCTCGGCAACTAAGCCTTCGGCTCAGAGCTGCTCTGATATCTCACATTTACCAAA  1663
             ||||||||||||||||||||||||||||||||||||||||||||||||||||||||||||
Sbjct  1226  TTGGAGCTCGGCAACTAAGCCTTCGGCTCAGAGCTGCTCTGATATCTCACATTTACCAAA  1285

Query  1664  AGGGCCTAGCTTTATCAAGTCAATCACACCAAAGCTACACGAGTGGAGAGATAATCAACT  1723
             ||||||||||||||||||||||||||||||||||||||||||||||||||||||||||||
Sbjct  1286  AGGGCCTAGCTTTATCAAGTCAATCACACCAAAGCTACACGAGTGGAGAGATAATCAACT  1345

Query  1724  ACATGAGCGTAGATGTCGAAAGGATTACGGATTTTATATGGCACCTTAACTCAATATGGA  1783
             ||||||||||||||||||||||||||||||||||||||||||||||||||||||||||||
Sbjct  1346  ACATGAGCGTAGATGTCGAAAGGATTACGGATTTTATATGGCACCTTAACTCAATATGGA  1405

Query  1784  TGTTACCCATCCAGATATCATTGGCAATCTATGTTTTACACATGAATCTAGGGAACGGGG  1843
             ||||||||||||||||||||||||||||||||||||||||||||||||||||||||||||
Sbjct  1406  TGTTACCCATCCAGATATCATTGGCAATCTATGTTTTACACATGAATCTAGGGAACGGGG  1465

Query  1844  CACTTGTGGCGCTAGGGGCAACCCTGATAGTGATGACCACCAACGTACCCCTGACAAGGA  1903
             |||||||||||||||||| |||||||||||||||||||||||||||||||||||||||||
Sbjct  1466  CACTTGTGGCGCTAGGGGAAACCCTGATAGTGATGACCACCAACGTACCCCTGACAAGGA  1525

Query  1904  TCCAAAAGGGATATCAAACTAAGATAATGGAATCCAAGGATGAAAGAATGAAATCTACTT  1963
             ||||||||||||||||||||||||||||||||||||||||||||||||||||||||||||
Sbjct  1526  TCCAAAAGGGATATCAAACTAAGATAATGGAATCCAAGGATGAAAGAATGAAATCTACTT  1585

Query  1964  CAGAGATTCTGCGAAATATGAAGACTATTAAACTTCAGGCATGGGATAGTTATTATCTCC  2023
             ||||||||||||||||||||||||||||||||||||||||||||||||||||||||||||
Sbjct  1586  CAGAGATTCTGCGAAATATGAAGACTATTAAACTTCAGGCATGGGATAGTTATTATCTCC  1645

Query  2024  ATAAGTTGGAAATCTTAAGAAAGGTGGAACATAATTGGCTGTGGAAATCACTAAGATTGT  2083
             ||||||||||||||||||||||||||||||||||||||||||||||||||||||||||||
Sbjct  1646  ATAAGTTGGAAATCTTAAGAAAGGTGGAACATAATTGGCTGTGGAAATCACTAAGATTGT  1705

Query  2084  CAGCCTTATCTGATTTTTTCTTCTGGGGATCACCTGCATTTATTTCTGTGGCAACCTTTT  2143
             ||||||||||||||||||||||||||||||||||||||||||||||||||||||||||||
Sbjct  1706  CAGCCTTATCTGATTTTTTCTTCTGGGGATCACCTGCATTTATTTCTGTGGCAACCTTTT  1765

Query  2144  CCGGATGTGTTATGATGGGCATACCACTGACTGCAGGCAGGGTCTTATCTACATTGGCCA  2203
             ||||||||||||||||||||||||||||||||||||||||||||||||||||||||||||
Sbjct  1766  CCGGATGTGTTATGATGGGCATACCACTGACTGCAGGCAGGGTCTTATCTACATTGGCCA  1825
```

```
Query  2204  CATTTCGGATGCTTCAAGATCCTATATTCAATTTGCCAGATTTGCTAAATGTCATAGCAC  2263
             ||||||||||||||||||||||||||||||||||||||||||||||||||||||||||||
Sbjct  1826  CATTTCGGATGCTTCAAGATCCTATATTCAATTTGCCAGATTTGCTAAATGTCATAGCAC  1885

Query  2264  GAGGAAAAGTTTCTGCTGATAGAGTTGCTTCCTACCTGCAGGAAGATGAGATTCAACCCG  2323
             ||||||||||||||||||||||||||||||||||||||||||||||||||||||||||||
Sbjct  1886  GAGGAAAAGTTTCTGCTGATAGAGTTGCTTCCTACCTGCAGGAAGATGAGATTCAACCCG  1945

Query  2324  ATGCAGTTGAATTTGTTCCTAAAGCTGAAACGCAATATGGGGTTGAGATAAAGAGTGGGA  2383
             ||||||||||||||||||||||||||||||||||||||||||||||||||||||||||||
Sbjct  1946  ATGCAGTTGAATTTGTTCCTAAAGCTGAAACGCAATATGGGGTTGAGATAAAGAGTGGGA  2005

Query  2384  AATTCAGCTGGGACACAGAATCAGGAACTCCCCCAACCCTTGATGGAATAGAATTACAAG  2443
             ||||||||||||||||||||||||||||||||||||||||||||||||||||||||||||
Sbjct  2006  AATTCAGCTGGGACACAGAATCAGGAACTCCCCCAACCCTTGATGGAATAGAATTACAAG  2065

Query  2444  TTAAGAGGGGAATGAAGGTGGCAATTTGTGGCACTGTTGGATCAGGAAAGTCAAGCTTGC  2503
             ||||||||||||||||||||||||||||||||||||||||||||||||||||||||||||
Sbjct  2066  TTAAGAGGGGAATGAAGGTGGCAATTTGTGGCACTGTTGGATCAGGAAAGTCAAGCTTGC  2125

Query  2504  TCTCTTGTGTACTAGGAGAGATGCCAAAATTGTCGGGGAATGTGAAGATCAGCGGTGAAG  2563
             ||||||||||||||||||||||||||||||||||||||||||||||||||||||||||||
Sbjct  2126  TCTCTTGTGTACTAGGAGAGATGCCAAAATTGTCGGGGAATGTGAAGATCAGCGGTGAAG  2185

Query  2564  TGGCATATGTTCCTCAGTCTCCTTGGATACTTACTGGAAATATCAAGGAGAATATTCTAT  2623
             ||||||||||||||||||||||||||||||||||||||||||||||||||||||||||||
Sbjct  2186  TGGCATATGTTCCTCAGTCTCCTTGGATACTTACTGGAAATATCAAGGAGAATATTCTAT  2245

Query  2624  TTGGAAAACCTTATGAGAGTGTTAAGTATGACAGAACAGTTGAAGCATGCGCGCTGAAAA  2683
             ||||||||||||||||||||||||||||||||||||||||||||||||||||||||||||
Sbjct  2246  TTGGAAAACCTTATGAGAGTGTTAAGTATGACAGAACAGTTGAAGCATGCGCGCTGAAAA  2305

Query  2684  AAGATTTTGAACTATTCCCTGCCGGTGATCTTACAGAAATTGGAGAAAGAGGGATAAATA  2743
             ||||||||||||||||||||||||||||||||||||||||||||||||||||||||||||
Sbjct  2306  AAGATTTTGAACTATTCCCTGCCGGTGATCTTACAGAAATTGGAGAAAGAGGGATAAATA  2365

Query  2744  TGAGCGGAGGTCAGAAGCAAAGAATACAAATCGCTCGTGCAGCTTACCAAGATGCTGATA  2803
             ||||||||||||||||||||||||||||||||||||||||||||||||||||||||||||
Sbjct  2366  TGAGCGGAGGTCAGAAGCAAAGAATACAAATCGCTCGTGCAGCTTACCAAGATGCTGATA  2425

Query  2804  TATATCTGCTCGATGACCCTTTCAGTGCCGTTGATGCTCACACAGGCACGCAGCTCTTTC  2863
             ||||||||||||||||||||||||||||||||||||||||||||||||||||||||||||
Sbjct  2426  TATATCTGCTCGATGACCCTTTCAGTGCCGTTGATGCTCACACAGGCACGCAGCTCTTTC  2485

Query  2864  AGGAGTGCTTGAGGGGGGTTCTCAAGGACAAGACCATACTTTATGTTACACACCAAGTTG  2923
             ||||||||||||||||||||||||||||||||||||||||||||||||||||||||||||
Sbjct  2486  AGGAGTGCTTGAGGGGGGTTCTCAAGGACAAGACCATACTTTATGTTACACACCAAGTTG  2545

Query  2924  AGTTTCTTCCTGCAGCAGATCTCATTCTGGTGATGCAAAATGGAAGAATTGCACAAGCTG  2983
             ||||||||||||||||||||||||||||||||||||||||||||||||||||||||||||
Sbjct  2546  AGTTTCTTCCTGCAGCAGATCTCATTCTGGTGATGCAAAATGGAAGAATTGCACAAGCTG  2605

Query  2984  GAACTTTTGAAGAACTACTGAAACAAAATATTGGATTTGAAGTTCTAGTTGGAGCACACA  3043
             ||||||||||||||||||||||||||||||||||||||||||||||||||||||||||||
Sbjct  2606  GAACTTTTGAAGAACTACTGAAACAAAATATTGGATTTGAAGTTCTAGTTGGAGCACACA  2665

Query  3044  ACCAGGCTTTAGAATCAATATTAACAGTTGAAAGCTCAAGTAGAATATCTGAAAAAGCAA  3103
             ||||||||||||||||||||||||||||||||||||||||||||||||||||||||||||
Sbjct  2666  ACCAGGCTTTAGAATCAATATTAACAGTTGAAAGCTCAAGTAGAATATCTGAAAAAGCAA  2725

Query  3104  TTACTGGTAGTGAGATGGATACAGAGAGTAACATAATTACAGAAACTAAGCAGGATTCAG  3163
             ||||||||||||||||||||||||||||||||||||||||||||||||||||||||||||
Sbjct  2726  TTACTGGTAGTGAGATGGATACAGAGAGTAACATAATTACAGAAACTAAGCAGGATTCAG  2785

Query  3164  AACACAGTCTCTGTGTAGAGATACCAGAAAAGGATGGAAGACTTGTGCAGGATGAGGAGA  3223
             ||||||||||||||||||||||||||||||||||||||||||||||||||||||||||||
Sbjct  2786  AACACAGTCTCTGTGTAGAGATACCAGAAAAGGATGGAAGACTTGTGCAGGATGAGGAGA  2845

Query  3224  GAGTCAAAGGAAGCATTGGAAAGGAAGTTTACTATTCTTACTTGACCTCTGTGAAAGGTG  3283
             ||||||||||||||||||||||||||||||||||||||||||||||||||||||||||||
Sbjct  2846  GAGTCAAAGGAAGCATTGGAAAGGAAGTTTACTATTCTTACTTGACCTCTGTGAAAGGTG  2905

Query  3284  GTGCCTTTGTCCCAATAATTCTTATAGCGCAATCATCATTTCAAGTGCTTCAGATAGCCA  3343
             ||||||||||||||||||||||||||||||||||||||||||||||||||||||||||||
Sbjct  2906  GTGCCTTTGTCCCAATAATTCTTATAGCGCAATCATCATTTCAAGTGCTTCAGATAGCCA  2965

Query  3344  GCAACTACTGGATGGCATCGGCATGTCCCACAGGTGATGATGTAGCACCAATAGCTGAGA  3403
```

```
                ||||||||||||||||||||||||||||||||||||||||||||||||||||||||||||
Sbjct  2966   GCAACTACTGGATGGCATCGGCATGTCCCACAGGTGATGATGTAGCACCAATAGCTGAGA  3025

Query  3404   AGATGAACTTCATACTTTTTGTTTATGTGCTTCTCGCTGTTGGAAGTTCCCTTTGCGTGC  3463
              ||||||||||||||||||||||||||||||||||||||||||||||||||||||||||||
Sbjct  3026   AGATGAACTTCATACTTTTTGTTTATGTGCTTCTCGCTGTTGGAAGTTCCCTTTGCGTGC  3085

Query  3464   TAGTGCGGGCATCATTTGTGGCTATAACAGGCCTTCAAACAGCAGAAAAGCTCTTTAGCA  3523
              ||||||||||||||||||||||||||||||||||||||||||||||||||||||||||||
Sbjct  3086   TAGTGCGGGCATCATTTGTGGCTATAACAGGCCTTCAAACAGCAGAAAAGCTCTTTAGCA  3145

Query  3524   ACATGCTGCACAGCATCTTTCACGCTCCTATGTCATTCTTTGACTCTACTCCTACTGGAA  3583
              ||||||||||||||||||||||||||||||||||||||||||||||||||||||||||||
Sbjct  3146   ACATGCTGCACAGCATCTTTCACGCTCCTATGTCATTCTTTGACTCTACTCCTACTGGAA  3205

Query  3584   GAATCTTAAACCGCGCATCCACCGACCAAAGTGTTGTGGACTTGGAAATTGCACTCAAGT  3643
              ||||||||||||||||||||||||||||||||||||||||||||||||||||||||||||
Sbjct  3206   GAATCTTAAACCGCGCATCCACCGACCAAAGTGTTGTGGACTTGGAAATTGCACTCAAGT  3265

Query  3644   TGGGTTGGTGTGCCCTCTCCATTATTCAGCTTCTTGGGACAATTGCTGTCATGTCGCAGG  3703
              ||||||||||||||||||||||||||||||||||||||||||||||||||||||||||||
Sbjct  3266   TGGGTTGGTGTGCCCTCTCCATTATTCAGCTTCTTGGGACAATTGCTGTCATGTCGCAGG  3325

Query  3704   TCGCATGGGAAGTATTTGTCCTCTTTATTCCAATAACAGCAGTTTATGTCTGGTACCAGC  3763
              ||||||||||||||||||||||||||||||||||||||||||||||||||||||||||||
Sbjct  3326   TCGCATGGGAAGTATTTGTCCTCTTTATTCCAATAACAGCAGTTTATGTCTGGTACCAGC  3385

Query  3764   AATACTACATACCAACCGCAAGGGAACTTGCTCGTTTATCTGGAGTTCAAAGAGCTCCAA  3823
              ||||||||||||||||||||||||||||||||||||||||||||||||||||||||||||
Sbjct  3386   AATACTACATACCAACCGCAAGGGAACTTGCTCGTTTATCTGGAGTTCAAAGAGCTCCAA  3445

Query  3824   TCCTCCATCACTTTGCAGAATCACTGTCAGGAGCAGCAACAATTCGTGCTTTCAACCAAA  3883
              ||||||||||||||||||||||||||||||||||||||||||||||||||||||||||||
Sbjct  3446   TCCTCCATCACTTTGCAGAATCACTGTCAGGAGCAGCAACAATTCGTGCTTTCAACCAAA  3505

Query  3884   AAGATCGCTTTGCTCATGCAAACCTTAGTCTCATAGATGGTCATTCAAGGCCATGGTTCC  3943
              ||||||||||||||||||||||||||||||||||||||||||||||||||||||||||||
Sbjct  3506   AAGATCGCTTTGCTCATGCAAACCTTAGTCTCATAGATGGTCATTCAAGGCCATGGTTCC  3565

Query  3944   ACAATATATCAGCACAAGAATGGCTATCTTTTAGACTGAATCAGCTTTCTACTTTTGTTT  4003
              ||||||||||||||||||||||||||||||||||||||||||||||||||||||||||||
Sbjct  3566   ACAATATATCAGCACAAGAATGGCTATCTTTTAGACTGAATCAGCTTTCTACTTTTGTTT  3625

Query  4004   TTGCCTTCTTCCTTGTTCTGCTAGTCACACTCCCCGAAGGAATTATAAATCCAAGCATTG  4063
              ||||||||||||||||||||||||||||||||||||||||||||||||||||||||||||
Sbjct  3626   TTGCCTTCTTCCTTGTTCTGCTAGTCACACTCCCCGAAGGAATTATAAATCCAAGCATTG  3685

Query  4064   CAGGATTAGCAGTAACATACGGCATCTATTTGAACTATTCACAAGCTGCAGTAATATGGA  4123
              ||||||||||||||||||||||||||||||||||||||||||||||||||||||||||||
Sbjct  3686   CAGGATTAGCAGTAACATACGGCATCTATTTGAACTATTCACAAGCTGCAGTAATATGGA  3745

Query  4124   ATATTTGCGGTACTGAAAACAAAATGATATCAGTTGAAAGGATTCTCCAGTATTCAGACC  4183
              ||||||||||||||||||||||||||||||||||||||||||||||||||||||||||||
Sbjct  3746   ATATTTGCGGTACTGAAAACAAAATGATATCAGTTGAAAGGATTCTCCAGTATTCAGACC  3805

Query  4184   TCGCCAGTGAAGCACCCCTCGTGATTGAAAATTGCAGACTATCAAGCACCTGGCCAGAAA  4243
              ||||||||||||||||||||||||||||||||||||||||||||||||||||||||||||
Sbjct  3806   TCGCCAGTGAAGCACCCCTCGTGATTGAAAATTGCAGACTATCAAGCACCTGGCCAGAAA  3865

Query  4244   CTGGAACAATTTCCTTCCAAAATTTACAGATACGATATGCTGAACACCTCCCGTCTGTTT  4303
              ||||||||||||||||||||||||||||||||||||||||||||||||||||||||||||
Sbjct  3866   CTGGAACAATTTCCTTCCAAAATTTACAGATACGATATGCTGAACACCTCCCGTCTGTTT  3925

Query  4304   TGAAAAACATCACATGCACATTTCCAGGAAGTAAGAAAATTGGTGTTGTGGGCAGGACAG  4363
              ||||||||||||||||||||||||||||||||||||||||||||||||||||||||||||
Sbjct  3926   TGAAAAACATCACATGCACATTTCCAGGAAGTAAGAAAATTGGTGTTGTGGGCAGGACAG  3985

Query  4364   GAAGTGGTAAATCAACCCTCACTCAAGCCCTTTTCCGGATCGTAGAACCCAGAGAAGGAA  4423
              ||||||||||||||||||||||||||||||||||||||||||||||||||||||||||||
Sbjct  3986   GAAGTGGTAAATCAACCCTCACTCAAGCCCTTTTCCGGATCGTAGAACCCAGAGAAGGAA  4045

Query  4424   GCATTATCATTGACAATATAGATATTTGCAAGATAGGTCTTCATGATTTGAGGTCAAGGC  4483
              ||||||||||||||||||||||||||||||||||||||||||||||||||||||||||||
Sbjct  4046   GCATTATCATTGACAATATAGATATTTGCAAGATAGGTCTTCATGATTTGAGGTCAAGGC  4105

Query  4484   TTAGTATTATTCCTCAAGATCCAACAATGTTCGACGGAACAGTTAGAGGAAACCTAGATC  4543
              ||||||||||||||||||||||||||||||||||||||||||||||||||||||||||||
```

```
Sbjct  4106  TTAGTATTATTCCTCAAGATCCAACAATGTTCGACGGAACAGTTAGAGGAAACCTAGATC  4165

Query  4544  CACTAGCACAGCACTCTGATACTGAAATCTGGGA-GCTATGGCGTATTTCCTTATTGAAA  4602
             |||||||||||||||||||||||||||||||||| ||| |||   | | ||   |||
Sbjct  4166  CACTAGCACAGCACTCTGATACTGAAATCTGGGAGGCTCTGGACAAATGCCAACTTG-GT  4224

Query  4603  GCTACTAATGTG------GTCAG----------TTTCACAGTGGCTGAAAACGGAGAAAA  4646
             | || ||||| |      | |||           |   ||||||||||||||||||||||
Sbjct  4225  GATA-TAATGCGTGCAAAGCCAGAAAAGCTAGAATCTTCAGTGGCTGAAAACGGAGAAAA  4283

Query  4647  CTGGAGTGTAGGTCAAAGGCAACTTTTCTGTCTCGGACGAGCCTTGCTAAAGAAAAGCAG  4706
             ||||||||||||||||||||||||||||||||||||||||||||||||||||||||||||
Sbjct  4284  CTGGAGTGTAGGTCAAAGGCAACTTTTCTGTCTCGGACGAGCCTTGCTAAAGAAAAGCAG  4343

Query  4707  CATTCTCATTCTAGATGAAGCAACAGCATCAGTTGATGCTGCAACTGATGCAGTGTTACA  4766
             ||||||||||||||||||||||||||||||||||||||||||||||||||||||||||||
Sbjct  4344  CATTCTCATTCTAGATGAAGCAACAGCATCAGTTGATGCTGCAACTGATGCAGTGTTACA  4403

Query  4767  AAAGATCATCAGTCAAGAGTTCAGAAATCGAACAGTTATCACAATAGCACACAGGATCCA  4826
             ||||||||||||||||||||||||||||||||||||||||||||||||||||||||||||
Sbjct  4404  AAAGATCATCAGTCAAGAGTTCAGAAATCGAACAGTTATCACAATAGCACACAGGATCCA  4463

Query  4827  TACAGTCATTAATAGCGATCTTGTCTTAGTCTTGAATGAAGGAAGAATAGCTGAATATGA  4886
             ||||||||||||||||||||||||||||||||||||||||||||||||||||||||||||
Sbjct  4464  TACAGTCATTAATAGCGATCTTGTCTTAGTCTTGAATGAAGGAAGAATAGCTGAATATGA  4523

Query  4887  TTCACCAGCAAAGCTATTGGAAAGAGAGGATTCTTTCTTCTCAAAACTGATAAAGGAGTA  4946
             ||||||||||||||||||||||||||||||||||||||||||||||||||||||||||||
Sbjct  4524  TTCACCAGCAAAGCTATTGGAAAGAGAGGATTCTTTCTTCTCAAAACTGATAAAGGAGTA  4583

Query  4947  TTTTATGAGATCCAAAAGTACGGCTTAG  4974
             ||||||||||||||||||||||||||||
Sbjct  4584  TTTTATGAGATCCAAAAGTACGGCTTAG  4611
```
